# Antimicrobial Activities of Aztreonam-Avibactam and Comparator Agents against Enterobacterales Analyzed by ICU and Non-ICU Wards, Infection Sources, and Geographic Regions: ATLAS Program 2016–2020

**DOI:** 10.3390/antibiotics12111591

**Published:** 2023-11-03

**Authors:** Denis Piérard, Elizabeth D. Hermsen, Michal Kantecki, Francis F. Arhin

**Affiliations:** 1 Department of Microbiology and Infection Control, Universitair Ziekenhuis Brussel, Vrije Universiteit Brussel, B-1090 Brussels, Belgium; denis.pierard@uzbrussel.be; 2 Pfizer Inc., New York, NY 10001, USA; elizabeth.hermsen@pfizer.com; 3 Pfizer International Operations, F-75668 Paris, France; michal.kantecki@pfizer.com; 4 Pfizer Inc., Kirkland, QC H9J 2M5, Canada

**Keywords:** antimicrobial activity, aztreonam-avibactam, aztreonam, avibactam, beta-lactams, beta-lactamase inhibitors, Enterobacterales, intensive care unit, wards, infection sources

## Abstract

Increasing antimicrobial resistance among multidrug-resistant (MDR), extended-spectrum β-lactamase (ESBL)- and carbapenemase-producing Enterobacterales (CPE), in particular metallo-β-lactamase (MBL)-positive strains, has led to limited treatment options in these isolates. This study evaluated the activity of aztreonam-avibactam (ATM-AVI) and comparator antimicrobials against Enterobacterales isolates and key resistance phenotypes stratified by wards, infection sources and geographic regions as part of the ATLAS program between 2016 and 2020. Minimum inhibitory concentrations (MICs) were determined per Clinical and Laboratory Standards Institute (CLSI) guidelines. The susceptibility of antimicrobials were interpreted using CLSI and European Committee on Antimicrobial Susceptibility Testing (EUCAST) breakpoints. A tentative pharmacokinetic/pharmacodynamic breakpoint of 8 µg/mL was considered for ATM-AVI activity. ATM-AVI inhibited ≥99.2% of Enterobacterales isolates across wards and ≥99.7% isolates across infection sources globally and in all regions at ≤8 µg/mL. For resistance phenotypes, ATM-AVI demonstrated sustained activity across wards and infection sources by inhibiting ≥98.5% and ≥99.1% of multidrug-resistant (MDR) isolates, ≥98.6% and ≥99.1% of ESBL-positive isolates, ≥96.8% and ≥90.9% of carbapenem-resistant (CR) isolates, and ≥96.8% and ≥97.4% of MBL-positive isolates, respectively, at ≤8 µg/mL globally and across regions. Overall, our study demonstrated that ATM-AVI represents an important therapeutic option for infections caused by Enterobacterales, including key resistance phenotypes across different wards and infection sources.

## 1. Introduction

Over the past few decades, the emergence of antimicrobial resistance in Enterobacterales (e.g., *Escherichia coli*, *Klebsiella* spp., *Enterobacter* spp., *Proteus* spp., *Serratia marcescens*, and *Citrobacter* spp.) has been a major global threat [1]. Antimicrobial resistance in Enterobacterales occurs through a wide range of mechanisms, and the treatment of infections caused by multidrug-resistant (MDR) Enterobacterales, including extended-spectrum β-lactamases (ESBL)-positive and carbapenem-resistant Enterobacterales (CRE), is highly challenging [2,3,4,5]. ESBL production in Enterobacterales is associated with resistance to third-generation cephalosporins, leading to increased mortality, length of stay, and costs [6]. CRE infections are associated with substantial healthcare burdens across both nosocomial and community settings [7,8]. World Health Organization (WHO) has categorized third-generation cephalosporin-resistant Enterobacterales and CRE as critical pathogens (‘priority 1’) with urgent need for the development of new and effective drugs [9].

The global dissemination of CRE has been reported in the last two decades [10,11]. A recent systematic review and meta-analysis revealed that the prevalence of carbapenem-resistant *K. pneumoniae* (CRKP) ranged from 0.13% to 22%, with a pooled prevalence of 5.43% (95% confidence interval (CI) 3.73–7.42), whereas the incidence of colonization ranged from 2% to 73%, with a pooled prevalence of 22.3% (95% CI 12.74–31.87). Additionally, the incidence of CRKP in patients from intensive care unit (ICU) wards was reported to be 24.6% (95% CI 12.37–36.83, I^2^ = 99.36) [12]. A systematic review and meta-analysis reported the death rate associated with CRE infections (26–44%) to be higher than that of infections caused by carbapenem-susceptible (CS) isolates (RR 2.05, 95% CI 1.56–2.69) [13]. Infection sources associated with CRE may vary, and the most common are bloodstream infections (BSI), respiratory tract infections (RTI), pneumonia and urinary tract infections (UTIs) [14,15,16]. A recent systematic review of mortality-related risk factors associated with CRE infections revealed hospital-related factors, including ICU stay (33.3%, 7/21 studies) and the sources of infection (e.g., BSI; 75.0%, 6/8 studies), as significant risk factors associated with CRE mortality [14]. Another recent systematic review and meta-analysis reported a significantly higher mortality in patients infected with CRKP compared to carbapenem-susceptible *K. pneumoniae* (CSKP) (crude OR, 2.8), with a pooled mortality of 54.3% (95% CI 47.51–61.02), 13.5% (95% CI 7.50–20.92), and 48.9% (95% CI 44.47–53.46) for BSIs, UTIs, and ICU admission associated with CRKP [17].

Carbapenem resistance in Enterobacterales is attributed to multiple resistance mechanisms, including carbapenemase production, plasmid-encoded ESBLs, and/or chromosomally encoded AmpCs, combined with porin mutations or the overexpression of efflux pumps [18,19,20,21]. Of these, the major resistance mechanism for carbapenem resistance is the production of carbapenemases, including Ambler class A serine-β-lactamases (e.g., *Klebsiella pneumoniae* carbapenamses (KPC)), class B metallo-β-lactamases (MBLs, e.g., New Delhi MBL (NDM), Verona integron-encoded MBL (VIM), and imipenemase (IMP)), and class D oxacillinases (e.g., OXA-48-like, OXA-232) [19,21]. MBL-positive Enterobacterales are disseminated globally with a substantial burden being contributed by Asia [22]. This was reported in a recent systematic review and meta-analysis in South Asia from 2010 to 2019, in which a pooled prevalence of 17% (95% CI 12–24) was observed for MBL-producing *E. coli* isolates [23]. MBL infections are associated with a substantial risk of mortality [24,25,26]. A case–control study from South Africa reported a significantly higher mortality rate in ICU patients infected with NDM-1-positive Enterobacterales compared to controls (55.3% vs. 14.7%; adjusted OR, 11.29; *p* < 0.001). Additionally, patients with BSIs had a significantly higher likelihood of in-hospital mortality (adjusted OR 8.84; 95% CI 1.09–71.55, *p* = 0.041) [25]. Another retrospective case–control study from India (2010–2014) reported a mortality rate of 44% (34/77) in patients with NDM-positive *K. pneumoniae* BSIs [26]. 

MBLs can hydrolyze penicillins, cephalosporins, and carbapenems, and hence have become a public health concern due to increasing antimicrobial resistance and limited treatment options. Aztreonam is unique compared to other β-lactams in that it is stable to hydrolysis by MBLs [27]. However, aztreonam is inactive against Enterobacterales producing ESBLs, KPC carbapenemases, or plasmid-encoded or chromosomally encoded AmpC β-lactamases [27,28,29,30,31]. Thus, MBL-positive isolates coproducing these other β-lactamases represent a major therapeutic challenge [22,32,33,34].

Avibactam, a non-β-lactam β-lactamase inhibitor, is capable of inhibiting Ambler class A and class C β-lactamases, and some class D carbapenemases [35]. Phase 3 trials of avibactam, in combination with aztreonam, to treat infections caused by MDR Gram-negative isolates including those expressing MBLs along with one or more additional β-lactamases have been recently completed (NCT03580044 and NCT03329092, results not published yet) [36,37]. The majority of previous surveillance studies on in vitro activity of aztreonam-avibactam (ATM-AVI) and comparator antimicrobial agents across regions in isolates from patients with respiratory tract infections (RTI), urinary tract infections (UTI), skin and soft tissue infections (SSTI), bloodstream infections (BSI), and intra-abdominal infections (IAI) have monitored their activity without the stratification of the infection sources [38,39,40,41]. Only two studies have assessed the resistance profile of these antimicrobial agents stratified by infection sources [3,42]. Importantly, these studies were either focused on specific geographic regions or pathogens or conducted over shorter study periods [3,42]. In the current study, we performed a detailed analysis of a large contemporary collection of clinical Enterobacterales isolates collected between 2016 and 2020 from the Antimicrobial Testing Leadership and Surveillance (ATLAS) program, a global surveillance database [43]. These isolates were collected from patients with RTI, UTI, SSTI, BSI, and IAI across Africa–Middle East (AfME), Asia–Pacific (APAC), Europe, Latin America (LATAM), and North America. This study characterized the activity of ATM-AVI and comparator antimicrobials against these isolates, including key resistant phenotypes prevalent among Enterobacterales stratified by wards (ICU and non-ICU), infection sources, and geographic regions.

## 2. Results

### 2.1. Distribution of Isolates

A total of 116,602 isolates of Enterobacterales from 351 sites in 63 countries were collected between 2016 and 2020. Among the wards, the highest number of Enterobacterales isolates were from non-ICU wards globally (64.2%) and across regions (58.5–69.3%). This pattern was observed in all resistant phenotypes across regions for MDR (CLSI/EUCAST, 58.0–67.4%/58.1–67.7%), ESBL (59.4–66.6%), CRE (CLSI/EUCAST, 50.2–68.4%/48.6–69.1%), and MBL-positive (47.5–70.0%) isolates (Table 1). For the infection sources, the highest number of Enterobacterales isolates globally were from UTI sources (23.4%), a trend that is consistent for all resistant phenotypes, including MDR (CLSI/EUCAST, 24.7%/24.8%), ESBL-positive (25.1%), and MBL-positive (26.0%) isolates, except CRE, for which the highest proportion were from RTI sources (CLSI/EUCAST, 26.5%/27.2%). Across the regions, however, there was variability observed in the highest number of isolates collected for Enterobacterales and resistant isolates from the different infection sources (Table 1).

The species distribution of Enterobacterales across wards demonstrated that the highest numbers were collected from non-ICU wards (*E. coli* (66.8%) and *K. pneumoniae* (60.4%)), a pattern that was observed for all the other species of Enterobacterales as well (Table S1). For infection sources, the highest number of *E. coli* and *K. pneumoniae* isolates were collected from UTI (25.5%) and RTI (29.3%) sources, respectively. Variability was observed in the highest number of isolates collected for other species of Enterobacterales from different infection sources (Table S1).

### 2.2. Activity of ATM-AVI and Other Antimicrobials against Enterobacterales across Wards

**Figure 1:**
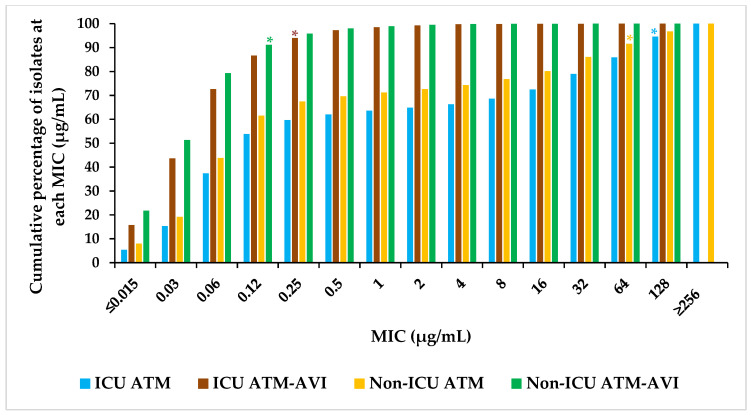
Percentage frequency distribution at aztreonam-avibactam and aztreonam MICs (µg/mL) for all Enterobacterales collected globally across wards, 2016–2020. ATM, aztreonam; ATM-AVI, aztreonam-avibactam; ICU, intensive care unit; MIC, minimum inhibitory concentration. * Denotes the MIC at which 90% of isolates are inhibited by ATM-AVI (ICU: MIC_90_ 0.25 µg/mL; non-ICU: MIC_90_ 0.12 µg/mL) and ATM (ICU: MIC_90_ 128 µg/mL; non-ICU: MIC_90_ 64 µg/mL).

ATM-AVI demonstrated potent antimicrobial activity (MIC_90_ 0.12–0.5 µg/mL), with ≥99.2% of isolates inhibited by ATM-AVI at ≤8 µg/mL (tentative PK/PD breakpoint) [35,41,44] across both ICU and non-ICU wards in all regions (Table 2). Analysis of the MIC frequency distributions revealed that while the MIC_90_ values for ATM-AVI against isolates from ICU and non-ICU wards were 0.25 µg/mL and 0.12 µg/mL, respectively, those for aztreonam were 128 µg/mL and 64 µg/mL, respectively (Figure 1). Of note, 0.15% (30/20,200) of isolates from ICU and 0.07% (41/56,533) from non-ICU wards were observed to have an MIC >8 µg/mL for ATM-AVI (Table S2). Among the comparator agents, applying both CLSI and EUCAST breakpoints, amikacin, ceftazidime–avibactam, colistin (available only per EUCAST), imipenem (available per CLSI; except in ICU isolates from APAC), meropenem, and tigecycline showed consistently high susceptibility across both ICU and non-ICU wards globally and in all regions (79.9–99.7%) (Table 2).

### 2.3. Activity of ATM-AVI and Other Antimicrobials against Enterobacterales across Infection Sources

**Figure 2:**
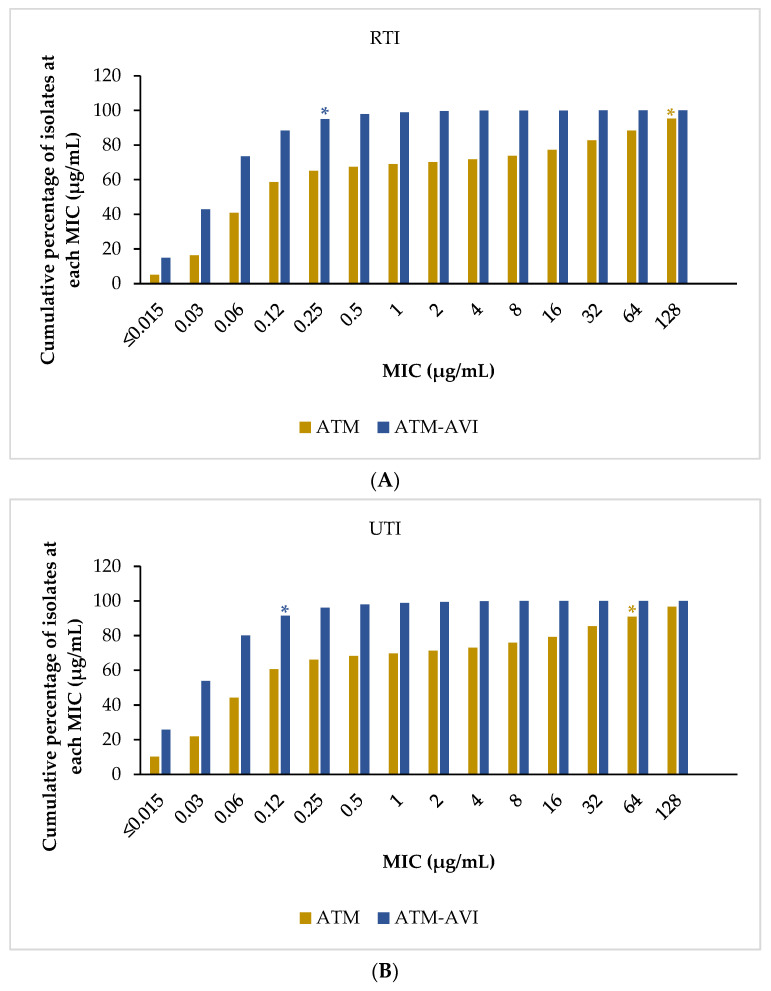

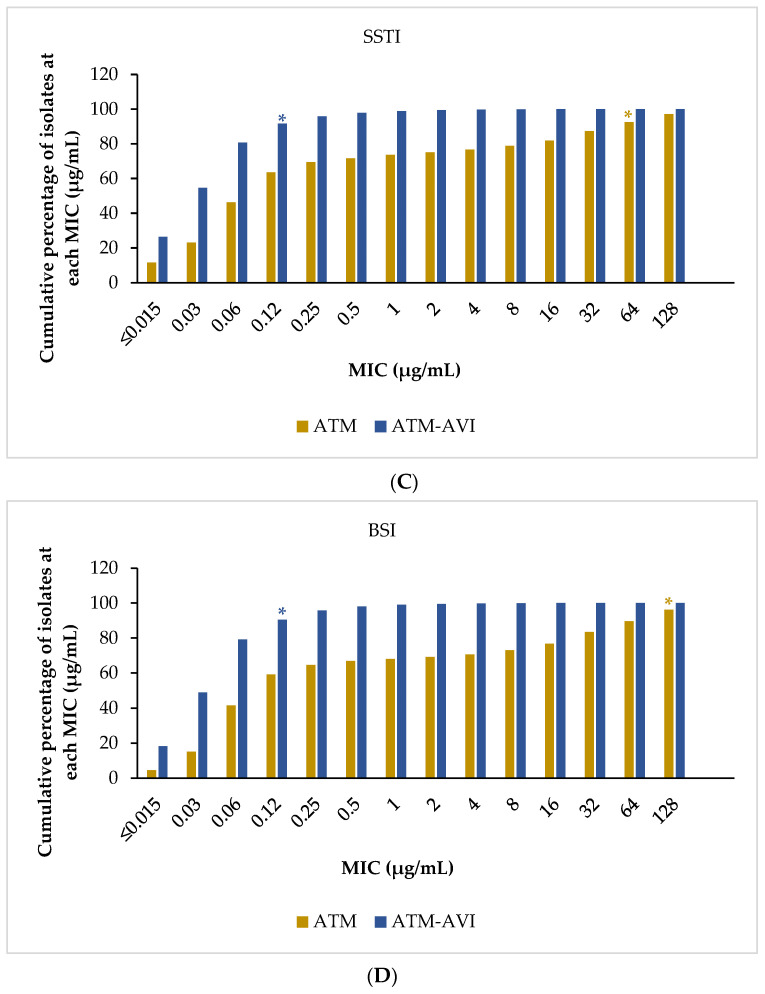

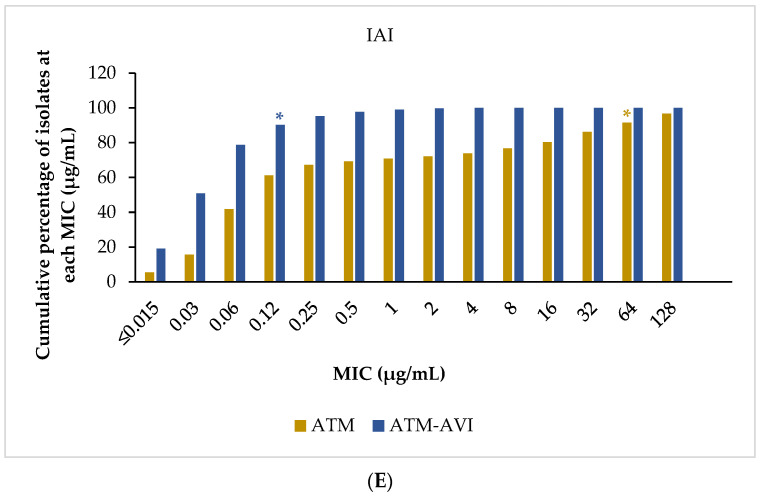
Percentage frequency distribution at aztreonam-avibactam and aztreonam MICs (µg/mL) for all Enterobacterales collected globally across infection sources, 2016–2020. (**A**) Respiratory tract infection sources. (**B**) Urinary tract infection sources. (**C**) Skin and soft tissue infection sources. (**D**) Bloodstream infection sources. (**E**) Intra-abdominal infection sources. ATM, aztreonam; ATM-AVI, aztreonam-avibactam; BSI, bloodstream infections; IAI, intra-abdominal infections; MIC, minimum inhibitory concentration; RTI, respiratory tract infection; SSTI, skin and soft tissue infections; UTI, urinary tract infection. * Denotes the MIC at which 90% of isolates are inhibited by ATM-AVI (RTI: MIC_90_ 0.25 µg/mL; UTI: MIC_90_ 0.12 µg/mL; SSTI: MIC_90_ 0.12 µg/mL; BSI: MIC_90_ 0.12 µg/mL; IAI: MIC_90_ 0.12 µg/mL) and ATM (RTI: MIC_90_ 128 µg/mL; UTI: MIC_90_ 64 µg/mL; SSTI: MIC_90_ 64 µg/mL; BSI: MIC_90_ 128 µg/mL; IAI: MIC_90_ 64 µg/mL).

Overall, ≥99.7% of isolates were inhibited by ATM-AVI at ≤8 µg/mL across all infection sources (RTI, UTI, SSTI, BSI, and IAI) globally and in all regions (Table 3). Globally, MIC frequency distribution revealed that the MIC_90_ values for ATM-AVI against isolates from the infection sources ranged from 0.12 to 0.25 µg/mL, and those for aztreonam ranged from 64 to 128 µg/mL (Figure 2 and Table S3). Importantly, 0.11% (22/20,197) of isolates from RTI sources, 0.09% (20/22,199) from UTI sources, 0.09% (17/18,117) from SSTI sources, 0.08% (14/18,159) from BSI sources, and 0.05% (6/13,341) from IAI sources were found to have MIC > 8 µg/mL for ATM-AVI (Table S3). Among comparator agents, amikacin, ceftazidime–avibactam, colistin (available only per EUCAST), imipenem (available per CLSI; except in SSTI isolates from APAC and LATAM), meropenem, and tigecycline demonstrated consistently high susceptibility across all infection sources globally and in all regions using both CLSI and EUCAST breakpoints (79.6–99.9%). Additionally, gentamicin showed high susceptibility in Europe and North America across all infection sources (82.1–93.8%) (Table 3).

### 2.4. Activity of ATM-AVI and Other Antimicrobials against Specific Resistance Phenotypes: MDR, ESBL-Positive, CRE, and MBL-Positive

ATM-AVI demonstrated potent antimicrobial activity (MIC_90_ 0.25–1 µg/mL) against MDR isolates, with ≥98.5% of isolates inhibited by ATM-AVI at ≤8 µg/mL across both ICU and non-ICU wards globally and in all regions (Table 4 and Table S4). A similar trend was observed against isolates from all infection sources, with ≥99.1% of isolates inhibited by ATM-AVI at ≤8 µg/mL globally and across all regions (Table 5 and Table S5). Applying both CLSI and EUCAST breakpoints, the comparator agents amikacin (except against ICU isolates from APAC (per CLSI/EUCAST) and LATAM (per EUCAST), and RTI isolates from APAC (per EUCAST)), ceftazidime–avibactam, colistin (available only per EUCAST), meropenem (except against ICU isolates collected globally, and from Europe, LATAM (per CLSI), and APAC (per CLSI/EUCAST); RTI isolates collected globally and from LATAM (per CLSI) and APAC (per CLSI/EUCAST); and BSI isolates from APAC and LATAM (per CLSI)), and tigecycline showed high susceptibility against MDR isolates across wards (80.0–99.0%, Table 4 and Table S4) and infection sources (80.0–99.9%, Table 5 and Table S5) globally and in all regions.

Among the ESBL-positive isolates, ≥98.6% of isolates across wards (Table 4 and Table S6) and ≥99.1% of isolates across infection sources (Table 5 and Table S7) were inhibited by ATM-AVI at ≤8 µg/mL globally and across regions. Using the CLSI and EUCAST breakpoints, the comparator agents amikacin (except against ICU sources collected globally and from LATAM (per EUCAST) and APAC (per CLSI/EUCAST); RTI and BSI (per CLSI/EUCAST); and UTI and SSTI sources (per EUCAST) from APAC), ceftazidime–avibactam (except against ICU and BSI sources from APAC (per CLSI/EUCAST)), colistin (available only per EUCAST), meropenem (except against ICU sources globally and from APAC (per CLSI/EUCAST), and from Europe and LATAM (per CLSI); RTI sources collected globally, from Europe and LATAM (per CLSI); RTI and BSI (per CLSI/EUCAST); UTI and SSTI (per CLSI) from APAC; and BSI sources (per CLSI) from LATAM), and tigecycline consistently showed high susceptibility across wards (79.5–99.5%, Table 4 and Table S6) and infection sources (79.5–100%, Table 5 and Table S7) globally and in all regions against the ESBL-positive isolates.

Against CRE isolates collected from wards, ATM-AVI demonstrated potent antimicrobial activity (MIC_90_ 0.5–1 µg/mL), with ≥96.8% isolates inhibited by ATM-AVI at ≤8 µg/mL globally and across regions (data limited to small number of isolates in North America [n ≤ 15]) (Table 4 and Table S8). Similarly, ATM-AVI was active against isolates from infection sources (MIC_90_ 0.5–4 µg/mL) with ≥90.9% of isolates inhibited by ATM-AVI at ≤8 µg/mL globally and across regions (data limited to small number of isolates in North America (n ≤ 18)) (Table 5 and Table S9). Among comparator agents, using both CLSI and EUCAST breakpoints, only colistin (available only per EUCAST; except in Europe and LATAM) and tigecycline (except RTI (per EUCAST) collected globally, from APAC, Europe, and LATAM; SSTI (per EUCAST) from Europe and North America; UTI (per EUCAST) from LATAM; and IAI (per EUCAST) from North America) demonstrated high susceptibility across wards (81.0–100%, Table 4 and Table S8) and infection sources (82.0–100%, Table 5 and Table S9) globally and across regions. 

Among MBL-positive isolates, ≥96.8% of isolates collected from wards (Table 4 and Table S10) and ≥97.4% of isolates collected from infection sources (Table 5 and Table S11) were inhibited by ATM-AVI at ≤8 µg/mL globally and across regions (data limited to a small number of isolates in AfME from IAI sources (n = 15) and North America from both wards and infection sources (n ≤ 140). Among comparator agents, applying the CLSI and EUCAST breakpoints, only colistin (available only per EUCAST) and tigecycline (except from RTI from Europe (per EUCAST)) demonstrated high susceptibility across wards (80.0–100%, Table 4 and Table S10) and infection sources (80.0–100%, Table 5 and Table S11) globally and in all regions (data limited to small number of isolates in AfME from IAI sources (n = 12) and North America from both wards and infection sources (n ≤ 7)).

## 3. Discussion

This study evaluated the in vitro antimicrobial susceptibilities of ATM-AVI and a panel of comparator agents against Enterobacterales isolates collected globally, across AfME, APAC, Europe, LATAM, and North America, from ICU and non-ICU wards and RTI, UTI, SSTI, BSI, and IAI infection sources between 2016 and 2020. The highest number of Enterobacterales (58.5–69.3%) and resistant isolates, including MDR, ESBL-positive, CRE, and MBL-positive isolates (47.5–70.0%), were collected from non-ICU wards globally and across regions. Among infection sources, the highest number of Enterobacterales (23.4%) and resistant phenotypes, including MDR, ESBL-positive, and MBL-positive isolates (25.1–26.0%), were collected from UTI sources globally, except CRE, for which the majority of isolates were from RTI sources (27.2%). Across regions, variability was observed for the highest number of isolates collected from different infection sources. Overall, ATM-AVI exhibited potent activity against Enterobacterales (MIC_90_ 0.12–0.5 µg/mL, ≥99.2 inhibited at ≤8 µg/mL) and all resistant phenotypes (MIC_90_ 0.25–4 mg/L, ≥98.5 inhibited at ≤8 µg/mL) from all wards and infection sources globally and across regions. Among comparator agents, amikacin, colistin, ceftazidime–avibactam, meropenem, and tigecycline were mostly active against all Enterobacterales (83.4–99.9%) and resistant phenotypes (79.5–99.9%), except for CRE and MBL-positive isolates, for which only colistin (except for MBL-positive isolates from Europe and LATAM) and tigecycline (80.0–100.0%) were notably active across wards and infection sources globally and in all regions. Altogether, these findings highlight the role of avibactam in potentiating the activity of aztreonam against Enterobacterales overall, including resistant phenotypes such as MDR, ESBL-positive, CRE, and MBL-positive isolates. 

In our study, ATM-AVI inhibited ≥99.9% of Enterobacterales isolates at ≤8 µg/mL (MIC_90_ 0.12–0.5 µg/mL) across both ICU (MIC_90_ 0.25 µg/mL) and non-ICU (MIC_90_ 0.12 µg/mL) wards globally. ATM-AVI also sustained potent in vitro activity (MIC_90_ 0.12–0.5 µg/mL) across all regions (AfME, APAC, Europe, LATAM, and North America). There is a lack of published studies on ATM-AVI activity stratified by wards. However, previous surveillance studies have observed potent ATM-AVI activity against overall Enterobacterales isolates across regions. A study by Karlowsky et al. conducted in 40 countries between 2012 and 2015 reported potent activity for ATM-AVI (MIC_90_ 0.12–0.25 µg/mL) against clinical isolates of Enterobacterales collected from AfME, APAC, Europe, LATAM, and North America [38]. A previous global surveillance study (2012–2013) also reported potent in vitro activity for ATM-AVI across regions including AfME, APAC, Europe, LATAM, and North America (MIC_90_ 0.12–0.25 µg/mL) [29]. Another surveillance study based on the SENTRY database (2019) reported that ≥99.8% Enterobacterales isolates were inhibited by ATM-AVI across all regions (Europe, APAC, and LATAM) [40]. Together, these results demonstrate the consistent in vitro activity of ATM-AVI across regions from 2012 to 2020. Among the panel of comparator agents, amikacin, ceftazidime–avibactam, colistin, imipenem, meropenem, and tigecycline displayed high rates of susceptibility against Enterobacterales isolates (79.9–99.7%) collected from ICU and non-ICU wards globally and in all regions. These findings are in agreement with previously published studies in which high susceptibility was observed for most of these antimicrobials against clinical isolates of Enterobacterales globally and across regions (79.6–98.8%; except tigecycline in Europe (76.9%)) [29,38,40].

ATM-AVI inhibited ≥99.9% Enterobacterales isolates at ≤8 µg/mL across all infection sources globally, with MIC_90_ value of 0.12 µg/mL observed for UTI, SSTI, BSI, and IAI sources and 0.25 µg/mL for RTI sources. Similar in vitro ATM-AVI activity was also observed irrespective of region, in which ≥99.7% Enterobacterales isolates from these infection sources were inhibited by ATM-AVI at ≤8 µg/mL (MIC_90_ of 0.12–0.25 µg/mL) in AfME, APAC, Europe, LATAM, and North America. This is in agreement with a study from the SENTRY database between 2019 and 2020 on Enterobacterales isolates collected from Europe, which showed that ≥99.6% isolates were inhibited by ATM-AVI irrespective of infection sources [3]. Overall, these findings suggest that ATM-AVI is potent against Enterobacterales isolates from various infection sources and its activity has been maintained over the years. Among the panel of comparator agents, amikacin, ceftazidime–avibactam, colistin, imipenem, meropenem, and tigecycline demonstrated high rates of susceptibility against Enterobacterales isolates irrespective of infection (79.6–99.9%) sources globally and across most of the regions, a pattern similar to that of isolates collected from wards. In contrast, the SENTRY study from Europe reported lower susceptibility rates for colistin (73.4–88.3%) and tigecycline (53.2–72.7%) compared to our study across infection sources [3].

Of note, our study identified 0.15% isolates from ICU wards and 0.07% from non-ICU wards with MIC > 8 µg/mL for ATM-AVI. Similarly, 0.11% isolates from RTI sources, 0.09% from UTI sources, 0.09% from SSTI sources, 0.08% from BSI sources, and 0.05% from IAI sources were also observed to have ATM-AVI MIC > 8 µg/mL. Potential resistance mechanisms responsible for such elevated MICs for ATM-AVI have been evaluated previously; a study from the INFORM database (2012–2017) in clinical isolates of Enterobacterales and a recent study assessing the reduced activity in clinical *E. coli* isolates suggested specific amino acid insertions in the penicillin-binding protein 3 (PBP3) sequence and an elevated expression of PER-type, VEB-type, and CMY-42 β-lactamases as potential resistance mechanisms to ATM-AVI [45,46], while other resistance mechanisms contributing to the reduction in ATM-AVI activity remain undefined and warrant further investigation [46]. These emerging resistance mechanisms could affect the therapeutic potential of ATM-AVI and thus require continuous surveillance efforts.

Our study demonstrated high potency of ATM-AVI (MIC_90_ 0.25–1 µg/mL) against MDR Enterobacterales, with ≥98.5% and ≥99.1% isolates, from wards and infection sources, respectively, inhibited at the tentative breakpoint of ≤8 µg/mL globally and in all regions. Our results are consistent with those of previous studies, which reported that 99.9% MDR isolates collected from United States (INFORM study, 2019–2021; MIC_90_ 0.25 µg/mL) and ≥99.3% MDR isolates from APAC, Europe, and LATAM (SENTRY study, 2019; MIC_90_ 0.5 µg/mL) were inhibited by ≤8 µg/mL ATM-AVI [40,47]. Our study also identified potent activity of ATM-AVI (MIC_90_ 0.25–1 µg/mL) against ESBL-positive isolates across wards and infection sources, with ≥98.6% and ≥99.1% isolates, respectively, inhibited at ≤8 µg/mL globally and in all regions. These findings are in line with results from previous studies reporting that addition of avibactam reduced the MICs of aztreonam against ESBL-positive Enterobacterales isolates (ATM-AVI, MIC_90_ ≤ 0.06–2 µg/mL) [48]. In our study, the potent activity of ATM-AVI (MIC_90_ 0.25–2 µg/mL) was also observed against CRE isolates with ≥96.8% and ≥90.9% of isolates, respectively, inhibited at ≤8 µg/mL. These findings are further corroborated by previous studies—INFORM study from US between 2019 and 2021 (99.6% CRE isolates inhibited, MIC_90_ 0.5 mg/L) [47]; SENTRY study from APAC, Europe, and LATAM in 2019 (≥98.7% CRE isolates inhibited, MIC_90_ 0.5 mg/L) [40]; and a global surveillance study by Kazmierczak et al. conducted in 40 countries between 2012 and 2014 (MIC_90_ 0.5 mg/L against KPC-positive isolates) [49]. Among the MBL-positive isolates, our study demonstrated potent ATM-AVI activity (MIC_90_ 0.25–4 mg/L), with ≥96.8% and ≥97.4% isolates from wards and infection sources, respectively, inhibited at ≤8 µg/mL globally and across regions (isolate count limited to ≤14 in North America). Likewise, previous studies for MBL-positive isolates have observed potent ATM-AVI activity globally and across regions—a multinational survey by Kazmierczak et al. conducted in 40 countries (2012–2014: MIC_90_ 0.5–1 µg/mL) [50]; a surveillance study by Karlowsky et al. across 208 medical centers in 40 countries (2012–2015: MIC_90_ 0.25–1 µg/mL) [38]; and two SENTRY studies, one in Europe, and the other in APAC, LATAM, and Europe (2019: MIC_90_ 0.5 µg/mL) [3,40]. Taken together, these findings emphasize the inhibitory effect of ATM-AVI against the resistant Enterobacterales phenotypes. Among the comparator agents, amikacin, ceftazidime–avibactam, colistin, meropenem, and tigecycline mostly showed high susceptibility in our study against MDR and ESBL-positive isolates (80.0–100.0%), whereas only colistin and tigecycline (80.0–100%) demonstrated mostly high susceptibility against CRE and MBL-positive isolates across wards and infection sources globally and in all regions. In contrast, previous SENTRY surveillance study (2019) has reported high susceptibility in few regions for meropenem (Western Europe and LATAM), amikacin (Western Europe and APAC), and colistin (APAC and LATAM) against MDR isolates (81.1–90.3%) and for only colistin (Western Europe and APAC) against CRE isolates (≥83.6%) [40]. For MBL-positive isolates, similar to the findings of this study, Karlowsky et al. (2012–2015) reported high susceptibility for colistin (except in LATAM, 68.8%) and tigecycline (82.4–100%) across regions [38]. 

Our study has a few limitations. A predefined number of isolates are collected for each species as part of the ATLAS program, hence the results of this study cannot be interpreted as prevalence or used for epidemiological data. The low number of samples for some resistant phenotypes and regions in this study should be taken into consideration while interpreting the findings. Agents such as meropenem–vaborbactam (approved in 2017) and ceftolozane–tazobactam (approved in 2014) were included in the antimicrobial panel of ATLAS in 2020 and hence have not been included in this study. Additionally, cefiderocol (approved in 2019) has not been added to the antimicrobial panel of ATLAS and could not be included in this study. Moreover, the current study does not ascertain the potential mechanisms of resistance in isolates with MIC > 8 µg/mL for ATM-AVI due to a lack of whole genome sequencing for isolates in the ATLAS program. Due to the unavailability of relevant data and the lack of granular information for ATM-AVI post 2020 in the ATLAS platform, we are unable to assess the potential impact of COVID-19 on the isolate distribution and susceptibility data included in this study. 

In conclusion, the results from this study demonstrated potent antimicrobial activity of ATM-AVI against Enterobacterales from all wards and infection sources globally and across regions. Furthermore, sustained activity was observed against resistant phenotypes, including MDR, ESBL-positive, CRE, and MBL-positive isolates, from all wards and infection sources globally and in all regions. The results of this large and comprehensive surveillance analysis further support the clinical development of ATM-AVI for treatment of Enterobacterales infections, including those caused by resistant phenotypes, such as CRE and MBL-positive strains. Additionally, the results from this study emphasize that ATM-AVI may be an important addition to the limited therapeutic options available against such isolates.

## 4. Materials and Methods

### 4.1. Bacterial Isolates

Non-duplicate clinical isolates of Enterobacterales were collected from the participating centers globally between 2016 and 2020 across different regions (AfME, APAC, Europe, LATAM, and North America) as part of the ATLAS surveillance program [43]. This program collects a predefined set of isolates of selected bacterial species from patients with specific infections from each participating medical center laboratory. Isolates are limited to one patient per year and accepted independent of patient hospital location [51]. The following species were included: *Enterobacter cloacae*, *Enterobacter hormaechi*, *Enterobacter kobei*, *Enterobacter ludwigii*, *Enterobacter asburiae*, *Escherichia coli*, *Klebsiella pneumoniae*, *Klebsiella oxytoca*, *Klebsiella aerogenes*, *Citrobacter koseri*, *Citrobacter freundii*, *Morganella morganii*, *Serratia marcescens*, *Proteus mirabilis*, *Citrobacter amalonaticus*, *Citrobacter braakii*, *Citrobacter farmer*, *Citrobacter* spp., *Enterobacter bugandensis*, *Enterobacter xiangfangensis*, *Klebsiella variicola*, *Proteus hauseri*, *Proteus vulgaris*, *Providencia alcalifaciens*, *Providencia rettgeri*, *Providencia* spp., *Providencia stuartii*, and *Raoultella ornithinolytica*. Bacterial isolates were collected from various wards (ICU and non-ICU—Medicine general, Pediatric general, and Surgery general) and infection sources (RTI, UTI, SSTI, BSI, and IAI) across patients of all ages (adult and pediatric). Isolates were shipped to the central laboratory, International Health Management Associates, Inc. (IHMA, Schaumburg, IL, USA) and confirmed using matrix-assisted laser desorption ionization–time-of-flight mass spectrometry (MALDI-TOF MS).

### 4.2. Antimicrobial Susceptibility Testing

Minimum inhibitory concentrations (MICs) were determined by IHMA using the reference broth microdilution methodology per the Clinical and Laboratory Standards Institute (CLSI) guidelines [52]. ATM-AVI and a panel of comparator antimicrobial agents including amikacin, aztreonam, cefepime, ceftazidime, ceftazidime–avibactam, ceftriaxone, ciprofloxacin, colistin, gentamicin, imipenem, levofloxacin, meropenem, piperacillin–tazobactam, and tigecycline were used for antimicrobial susceptibility testing. The susceptibility of antimicrobial agents were interpreted using CLSI M100 (33rd ed.) and European Committee on Antimicrobial Susceptibility Testing (EUCAST, version 13.0) breakpoints, wherever applicable [52,53]. ATM-AVI was tested with avibactam at a fixed concentration of 4 mg/L. A provisional pharmacokinetic/pharmacodynamic susceptible breakpoint of ≤8 mg/L was used for ATM-AVI for comparison owing to lack of approved clinical breakpoints [35,41,44]. For colistin, since the susceptible breakpoints per CLSI are not available, only EUCAST data were reported. Isolates of *Morganella morganii*, *Proteus* spp., *Providencia* spp., and *Serratia marcescens* were excluded from the analysis of colistin data because of their intrinsic resistance [54]. For tigecycline, MICs were interpreted using FDA-approved breakpoints due to lack of MIC interpretative criteria per CLSI. For the analysis of tigecycline data, isolates of *Morganella morganii*, *Proteus* spp., and *Providencia* spp. were excluded due to intrinsic resistance [55]. All antimicrobials were not tested in each year of the surveillance, hence varying numbers of isolates were recorded against the different antimicrobials. 

All the data were collected and presented as percentage of susceptible (%S) isolates and MIC_90_ based on CLSI and EUCAST guidelines for all identified organisms. No statistical analysis was performed as part of this study. In this study, susceptibility ≥80.0% was categorized as high.

### 4.3. Resistance Phenotypes Definitions

In this study, the CRE (CLSI/EUCAST) phenotype was defined as isolates of any Enterobacterales species resistant to meropenem per the ATLAS program.

The MDR (CLSI) phenotype was defined as resistance to any three of the following drug classes per the ATLAS program: aminoglycosides (amikacin or gentamicin), antipseudomonal penicillins (piperacillin–tazobactam), carbapenems (ertapenem, doripenem, imipenem, or meropenem), cephalosporins (ceftaroline, ceftazidime, ceftriaxone, or cefepime), quinolones (ciprofloxacin), sulfonamides (trimethoprim sulfa), glycylcyclines (tigecycline), monobactams (aztreonam), penicillins (ampicillin), penicillins plus beta lactamase inhibitor (ampicillin–sulbactam or amoxicillin–clavulanate), tetracyclines (minocycline), and polymyxins (colistin). The MDR (EUCAST) phenotype was defined as resistance to any three of the above drug classes except doripenem, tigecycline, and minocycline.

Isolates of Enterobacterales with MICs to meropenem of ≥2 mg/L and/or a ceftazidime–avibactam MIC ≥ 16 mg/L and/or ATM-AVI MIC ≥ 16 mg/L were screened for the presence of extended-spectrum β-lactamases (ESBL) genes—*bla*_SHV_, *bla*_TEM_, *bla*_CTX-M_, *bla*_VEB_, *bla*_PER_, and *bla*_GES_—using multiplex PCR assays followed by full-gene DNA sequencing as previously described. Additionally, isolates of *E. coli*, *K. pneumoniae*, *K. oxytoca*, and *P. mirabilis* with a ceftazidime or aztreonam MIC ≥ 2 mg/L qualified for the above screening.

In this study, isolates carrying class B MBL genes—*bla*_NDM_, *bla*_IMP_, and *bla*_VIM_—(as data were available only for these three subsets on the ATLAS database) were identified by PCR and sequencing.

## Figures and Tables

**Table 1:** Distribution of Enterobacterales isolates collected globally and across different regions stratified by wards and infection sources, 2016–2020.

		Wards n (%)			Infection Sources n (%)					
	N	n ^a,b^	ICU	Non-ICU	n ^a,c^	RTI	UTI	SSTI	BSI	IAI
**Global**										
Enterobacterales ^d^	116,602	109,197	25,093	70,054	116,247	25,575	27,254	22,040	23,450	16,873
			(23.0)	(64.2)		(22.0)	(23.4)	(19.0)	(20.2)	(14.5)
MDR CLSI	36,305	34,330	9202	21,691	36,258	7772	8955	6381	7836	5212
			(26.8)	(63.2)		(21.4)	(24.7)	(17.6)	(21.6)	(14.4)
MDR EUCAST	39,700	37,488	9857	23,771	39,643	8393	9815	6927	8651	5728
			(26.3)	(63.4)		(21.2)	(24.8)	(17.5)	(21.8)	(14.4)
ESBL	20,303	19,149	5312	11,837	20,266	4463	5093	3508	4227	2855
			(27.7)	(61.8)		(22)	(25.1)	(17.3)	(20.9)	(14.1)
CRE CLSI	5576	5206	2058	2771	5564	1475	1142	870	1328	706
			(39.5)	(53.2)		(26.5)	(20.5)	(15.6)	(23.9)	(12.7)
CRE EUCAST	4388	4101	1681	2120	4379	1190	892	620	1096	551
			(41.0)	(51.7)		(27.2)	(20.4)	(14.2)	(25.0)	(12.6)
MBL-positive	1877	1765	728	909	1874	436	487	327	430	193
			(41.3)	(51.5)		(23.3)	(26.0)	(17.4)	(22.9)	(10.3)
**Africa–Middle East**										
Enterobacterales	8990	8531	1839	5812	8964	1555	2268	2390	1565	1151
			(21.6)	(68.1)		(17.3)	(25.3)	(26.7)	(17.5)	(12.8)
MDR CLSI	3627	3414	805	2298	3616	576	1010	852	762	413
			(23.9)	(67.3)		(15.9)	(27.9)	(23. 6)	(21.1)	(11.4)
MDR EUCAST	3935	3709	869	2501	3923	626	1072	946	820	454
			(23.4)	(67.4)		(16.0)	(27.3)	(24.1)	(20.9)	(11.6)
ESBL	2176	2055	503	1369	2175	390	565	510	467	237
			(24.5)	(66.6)		(17.9)	(26.0)	(23.4)	(21.5)	(10.9)
CRE CLSI	337	312	115	176	337	68	76	63	88	41
			(36.9)	(56.4)		(20.2)	(22.6)	(18.7)	(26.1)	(12.2)
CRE EUCAST	252	229	89	121	252	54	51	44	73	30
			(38.9)	(52.8)		(21.4)	(20.2)	(17.5)	(29.0)	(11.9)
MBL-positive	190	177	58	104	190	24	59	38	54	15
			(32.8)	(58.8)		(12.6)	(31.1)	(20.0)	(28.4)	(7.9)
**Asia–Pacific**										
Enterobacterales	21,653	20,577	4052	14,268	21,618	5525	5339	3239	4247	3182
			(19.7)	(69.3)		(25.6)	(24.7)	(15.0)	(19.6)	(14.7)
MDR CLSI	8781	8404	2029	5605	8775	2127	2354	1239	1766	1277
			(24.1)	(66.7)		(24.2)	(26.8)	(14.1)	(20.1)	(14.6)
MDR EUCAST	9441	9018	2135	6044	9435	2264	2540	1336	1908	1375
			(23.7)	(67.0)		(24.0)	(26.9)	(14.2)	(20.2)	(14.6)
ESBL	4106	3881	1025	2472	4098	1023	1105	619	819	526
			(26.4)	(63.7)		(25.0)	(27.0)	(15.1)	(20.0)	(12.8)
CRE CLSI	1691	1588	653	831	1686	536	398	206	365	172
			(41.1)	(52.3)		(31.8)	(23.6)	(12.2)	(21.6)	(10.2)
CRE EUCAST	1531	1438	602	744	1526	479	356	184	346	153
			(41.9)	(51.7)		(31.4)	(23.3)	(12.1)	(22.7)	(10.0)
MBL-positive	735	684	317	325	733	185	207	109	176	56
			(46.3)	(47.5)		(25.2)	(28.2)	(14.9)	(24.0)	(7.6)
**Europe**										
Enterobacterales	55,919	52,177	12,919	32,797	55,688	12,770	12,086	11,008	10,938	8116
			(24.8)	(62.9)		(22.9)	(21.7)	(19.8)	(19.6)	(14.6)
MDR CLSI	14,785	13,944	4126	8606	14,764	3473	3297	2731	3140	2056
			(29.6)	(61.7)		(23.5)	(22.3)	(18.5)	(21.3)	(13.9)
MDR EUCAST	16,435	15,474	4473	9583	16,408	3809	3692	2971	3540	2308
			(28.9)	(61.9)		(23.2)	(22.5)	(18.1)	(21.6)	(14.1)
ESBL	8807	8246	2453	5014	8785	2148	2154	1491	1735	1174
			(29.7)	(60.8)		(24.5)	(24.5)	(17.0)	(19.7)	(13.4)
CRE CLSI	2241	2078	824	1120	2238	607	394	381	533	302
			(39.7)	(53.9)		(27.1)	(17.6)	(17.0)	(23.8)	(13.5)
CRE EUCAST	1649	1534	637	801	1648	456	286	244	417	231
			(41.5)	(52.2)		(27.7)	(17.4)	(14.8)	(25.3)	(14.0)
MBL-positive	604	571	235	311	604	179	139	97	111	78
			(41.2)	(54.5)		(29.6)	(23.0)	(16.1)	(18.4)	(12.9)
**Latin America**										
Enterobacterales	16,501	15,608	3752	9133	16,465	2714	4371	2926	3556	2814
			(24.0)	(58.5)		(16.5)	(26.6)	(17.8)	(21.6)	(17.1)
MDR CLSI	6640	6333	1736	3675	6633	1005	1706	1188	1548	1170
			(27.4)	(58.0)		(15.2)	(25.7)	(17.9)	(23.3)	(17.6)
MDR EUCAST	7083	6747	1825	3923	7074	1047	1844	1258	1656	1251
			(27.0)	(58.1)		(14.8)	(26.1)	(17.8)	(23.4)	(17.7)
ESBL	4115	3950	1077	2346	4110	637	999	751	906	798
			(27.3)	(59.4)		(15.5)	(24.3)	(18.3)	(22.0)	(19.4)
CRE CLSI	1133	1076	425	540	1130	207	241	187	321	164
			(39.5)	(50.2)		(18.3)	(21.3)	(16.5)	(28.4)	(14.5)
CRE EUCAST	864	819	333	398	862	168	181	136	248	122
			(40.7)	(48.6)		(19.5)	(21.0)	(15.8)	(28.8)	(14.2)
MBL-positive	326	313	113	155	326	41	76	78	89	41
			(36.1)	(49.5)		(12.6)	(23.3)	(23.9)	(27.3)	(12.6)
**North America**										
Enterobacterales	13,539	12,304	2531	8044	13,512	3011	3190	2477	3144	1610
			(20.6)	(65.4)		(22.3)	(23.6)	(18.3)	(23.3)	(11.9)
MDR CLSI	2472	2235	506	1507	2470	591	588	371	620	296
			(22.6)	(67.4)		(23.9)	(23.8)	(15.0)	(25.1)	(12.0)
MDR EUCAST	2806	2540	555	1720	2803	647	667	416	727	340
			(21.9)	(67.7)		(23.1)	(23.8)	(14.8)	(25.9)	(12.1)
ESBL	1099	1017	254	636	1098	265	270	137	300	120
			(25.0)	(62.5)		(24.1)	(24.6)	(12.5)	(27.3)	(10.9)
CRE CLSI	174	152	41	104	173	57	33	33	21	27
			(27.0)	(68.4)		(32.9)	(19.1)	(19.1)	(12.1)	(15.6)
CRE EUCAST	92	81	20	56	91	33	18	12	12	15
			(24.7)	(69.1)		(36.3)	(19.8)	(13.2)	(13.2)	(16.5)
MBL-positive	22	20	5	14	21	7	6	5	NA	3
			(25.0)	(70.0)		(33.3)	(28.6)	(23.8)		(14.3)

^a^ Does not include isolates from wards/infection sources for which information was not specified or available. ^b^ The number of isolates mentioned correspond to ICU, non-ICU, and other sources (clinic/office, emergency room, nursing home/rehab). ^c^ The number of isolates mentioned correspond to RTI, UTI, SSTI, BSI, IAI, and other infection sources (nervous system; the head, ears, eyes, nose, and throat (HEENT), and instruments). ^d^ Includes *Enterobacter cloacae*, *Enterobacter hormaechi*, *Enterobacter kobei*, *Enterobacter ludwigii*, *Enterobacter asburiae*, *Escherichia coli*, *Klebsiella pneumoniae*, *Klebsiella oxytoca*, *Klebsiella aerogenes*, *Citrobacter koseri*, *Citrobacter freundii*, *Morganella morganii*, *Serratia marcescens*, *Proteus mirabilis*, *Citrobacter amalonaticus*, *Citrobacter braakii*, *Citrobacter farmer*, *Citrobacter* spp., *Enterobacter bugandensis*, *Enterobacter xiangfangensis*, *Klebsiella variicola*, *Proteus hauseri*, *Proteus vulgaris*, *Providencia alcalifaciens*, *Providencia rettgeri*, *Providencia* spp., *Providencia stuartii*, *Raoultella ornithinolytica*. % indicates n (from specific ward or infection source)/total n from wards or infection sources. BSI, blood stream infections; CRE, carbapenem-resistant Enterobacterales; ESBL, extended-spectrum beta-lactamase; IAI, intra-abdominal infections; ICU, intensive care unit; MBL, metallo-β-lactamase; N, total number of isolates; n, number of isolates from wards/infection sources; NA, not available; RTI, respiratory tract infections; SSTI, skin and soft tissue infection; UTI, urinary tract infections.

**Table 2:** In vitro activity of ATM-AVI and comparator agents tested against Enterobacterales isolates across regions stratified by wards from 2016 to 2020.

	MIC_90_ (µg/mL) (% S, CLSI/%S, EUCAST ^a^)			
	ICU		Non-ICU	
Global (N = 116,602) ^b^	n ^c^		n ^c^	
Aztreonam-avibactam ^d^	20,200	0.25 (99.9)	56,533	0.12 (99.9)
Aztreonam	20,799	128 (66.3/66.3)	58,531	64 (74.3/74.3)
Amikacin	25,093	8 (94.3/91.9)	70,054	8 (97.3/95.5)
Cefepime	25,093	64 (70.5/73.1)	70,054	32 (77.2/79.5)
Ceftazidime	25,093	128 (67.7/67.7)	70,054	64 (75.4/75.4)
Ceftazidime–avibactam	20,799	1 (96.1/96.1)	58,532	0.5 (98.2/98.2)
Ceftriaxone	10,736	32 (66.3/67.7)	31,851	32 (71.0/72.1)
Ciprofloxacin	14,357	8 (61.6/65.7)	38,203	8 (64.5/68.8)
Colistin ^e,f^	17,848	1 (NA/95.9)	48,942	1 (NA/97.4)
Gentamicin	14,357	32 (77.6/76.7)	38,203	32 (83.0/81.9)
Imipenem ^g^	20,799	4 (81.6/NA)	58,532	2 (84.3/NA)
Levofloxacin	25,093	16 (68.3/73.5)	70,054	16 (69.1/74.0)
Meropenem	25,093	0.5 (91.0/93.3)	70,054	0.12 (95.5/97.0)
Piperacillin–tazobactam	25,093	128 (73.0/73.0)	70,054	64 (81.0/81.0)
Tigecycline ^h,i,j^	23,374	1 (97.8/98.2)	62,681	1 (98.3/98.0)
**Africa–Middle East (N = 8990)**				
Aztreonam-avibactam ^d^	1686	0.25 (100)	5361	0.12 (99.9)
Aztreonam	1686	128 (63.0/63.0)	5361	64 (68.8/68.8)
Amikacin	1839	8 (96.1/93.1)	5812	8 (97.6/95.5)
Cefepime	1839	64 (64.1/66.9)	5812	32 (68.9/71.7)
Ceftazidime	1839	64 (62.6/62.6)	5812	64 (69.2/69.2)
Ceftazidime–avibactam	1686	0.5 (96.5/96.5)	5361	0.5 (97.8/97.8)
Ceftriaxone	584	32 (60.6/61.8)	2365	32 (69.6/70.6)
Ciprofloxacin	1255	8 (56.7/64.1)	3447	8 (52.8/58.9)
Colistin ^e,f^	1443	1 (NA/97.1)	4475	1 (NA/98.1)
Gentamicin	1255	32 (74.4/73.7)	3447	32 (74.4/73.0)
Imipenem ^g^	1686	2 (83.4/NA)	5361	2 (84.7/NA)
Levofloxacin	1839	16 (66.8/75.1)	5812	16 (62.7/69.8)
Meropenem	1839	0.5 (92.7/95.2)	5812	0.12 (96.4/97.9)
Piperacillin–tazobactam	1839	128 (74.2/74.2)	5812	64 (79.8/79.8)
Tigecycline ^h,i,j^	1714	1 (97.9/97.7)	5107	1 (98.4/97.9)
**Asia–Pacific (N = 21,653)**				
Aztreonam-avibactam ^d^	3184	0.5 (99.2)	10,913	0.25 (99.8)
Aztreonam	3783	128 (56.0/56.0)	12,911	128 (68.7/68.7)
Amikacin	4052	128 (86.3/84.2)	14,268	8 (95.6/93.8)
Cefepime	4052	64 (58.5/61.5)	14,268	64 (71.6/74.4)
Ceftazidime	4052	256 (55.6/55.6)	14,268	128 (70.0/70.0)
Ceftazidime–avibactam	3783	16 (90.0/90.0)	12,911	0.5 (96.5/96.5)
Ceftriaxone	1190	32 (56.8/58.4)	5682	32 (63.0/64.3)
Ciprofloxacin	2862	8 (48.1/53.4)	8586	8 (56.4/62.2)
Colistin ^e,f^	3291	1 (NA/94.5)	10,944	1 (NA/96.3)
Gentamicin	2862	32 (69.0/67.9)	8586	32 (78.9/77.6)
Imipenem ^g^	3783	16 (74.3/NA)	12,911	2 (82.9/NA)
Levofloxacin	4052	16 (55.1/61.0)	14,268	16 (61.2/67.2)
Meropenem	4052	32 (83.4/85.1)	14,268	0.25 (93.9/94.8)
Piperacillin–tazobactam	4052	128 (66.9/66.9)	14,268	128 (80.0/80.0)
Tigecycline ^h,i,j^	3738	2 (96.7/95.8)	12,776	1 (98.0/95.9)
**Europe (N = 55,919)**				
Aztreonam-avibactam ^d^	10,124	0.25 (100)	26,062	0.12 (100)
Aztreonam	10,124	128 (70.4/70.4)	26,062	64 (77.8/77.8)
Amikacin	12,919	8 (95.9/93.6)	32,797	4 (97.6/96.0)
Cefepime	12,919	32 (74.9/77.2)	32,797	32 (80.8/82.8)
Ceftazidime	12,919	128 (71.7/71.7)	32,797	32 (78.2/78.2)
Ceftazidime–avibactam	10,124	1 (97.6/97.6)	26,062	0.5 (98.8/98.8)
Ceftriaxone	6264	32 (68.5/69.9)	16,521	32 (74.1/75.2)
Ciprofloxacin	6655	8 (67.9/71.0)	16,276	8 (70.3/73.5)
Colistin ^e,f^	8617	1 (NA/96.1)	21,651	0.5 (NA/98.0)
Gentamicin	6655	32 (81.4/80.6)	16,276	32 (86.4/85.3)
Imipenem ^g^	10,124	4 (83.1/NA)	26,062	2 (84.5/NA)
Levofloxacin	12,919	16 (72.3/76.9)	32,797	16 (73.8/77.8)
Meropenem	12,919	0.25 (92.7/95.1)	32,797	0.12 (96.0/97.6)
Piperacillin–tazobactam	12,919	128 (73.6/73.6)	32,797	64 (80.9/80.9)
Tigecycline ^h,i,j^	12,013	1 (98.1/98.8)	29,332	1 (98.4/98.8)
**Latin America (N = 16,501)**				
Aztreonam-avibactam ^d^	3302	0.25 (100)	7810	0.12 (100)
Aztreonam	3302	128 (58.5/58.5)	7810	128 (66.1/66.1)
Amikacin	3752	16 (93.2/89.6)	9133	8 (96.3/93.6)
Cefepime	3752	64 (60.5/63.9)	9133	64 (67/69.6)
Ceftazidime	3752	128 (59.6/59.6)	9133	64 (67.6/67.6)
Ceftazidime–avibactam	3302	1 (96.5/96.5)	7810	0.5 (98.0/98.0)
Ceftriaxone	1630	64 (57.1/58.0)	4392	32 (61.5/62.4)
Ciprofloxacin	2122	8 (51.9/56.6)	4741	8 (53.8/58.6)
Colistin ^e,f^	2828	1 (NA/94.9)	6571	1 (NA/96.4)
Gentamicin	2122	32 (69.9/68.6)	4741	32 (75.5/74.2)
Imipenem ^g^	3302	8 (79.9/NA)	7810	2 (82.8/NA)
Levofloxacin	3752	16 (60.4/66.9)	9133	16 (59.1/64.9)
Meropenem	3752	8 (87.6/91.1)	9133	0.25 (93.2/95.6)
Piperacillin–tazobactam	3752	128 (69.1/69.1)	9133	128 (77.1/77.1)
Tigecycline ^h,i,j^	3496	1 (97.8/98.8)	8204	1 (98.5/98.2)
**North America (N = 13,539)**				
Aztreonam-avibactam ^d^	1904	0.12 (100)	6387	0.12 (99.9)
Aztreonam	1904	32 (81.2/81.2)	6387	16 (85.9/85.9)
Amikacin	2531	4 (99.1/97.6)	8044	4 (99.5/98.6)
Cefepime	2531	8 (86.8/89.3)	8044	4 (90.0/91.7)
Ceftazidime	2531	32 (82.9/82.9)	8044	16 (86.4/86.4)
Ceftazidime–avibactam	1904	0.5 (99.7/99.7)	6388	0.5 (99.7/99.7)
Ceftriaxone	1068	32 (81.6/83.3)	2891	32 (84.7/86.1)
Ciprofloxacin	1463	8 (77.1/80.5)	5153	8 (77.6/80.7)
Colistin ^e,f^	1669	0.5 (NA/98.1)	5301	0.5 (NA/97.9)
Gentamicin	1463	2 (91.5/91.0)	5153	2 (92.0/91.0)
Imipenem ^g^	1904	2 (89.8/NA)	6388	2 (87.4/NA)
Levofloxacin	2531	8 (81.6/84.7)	8044	8 (80.4/83.5)
Meropenem	2531	0.12 (98.1/99.2)	8044	0.12 (98.6/99.3)
Piperacillin–tazobactam	2531	32 (84.6/84.6)	8044	16 (88.3/88.3)
Tigecycline ^h,i,j^	2413	1 (98.1/99.1)	7262	1 (98.4/98.9)

^a^ Data includes percentage isolates susceptible at increased exposure. ^b^ Includes *Enterobacter cloacae*, *Enterobacter hormaechi*, *Enterobacter kobei*, *Enterobacter ludwigii*, *Enterobacter asburiae*, *Escherichia coli*, *Klebsiella pneumoniae*, *Klebsiella oxytoca*, *Klebsiella aerogenes*, *Citrobacter koseri*, *Citrobacter freundii*, *Morganella morganii*, *Serratia marcescens*, *Proteus mirabilis*, *Citrobacter amalonaticus*, *Citrobacter braakii*, *Citrobacter farmer*, *Citrobacter* spp., *Enterobacter bugandensis*, *Enterobacter xiangfangensis*, *Klebsiella variicola*, *Proteus hauseri*, *Proteus vulgaris*, *Providencia alcalifaciens*, *Providencia rettgeri*, *Providencia* spp., *Providencia stuartii*, *Raoultella ornithinolytica*. ^c^ Not all drugs in the panel were tested every year. ^d^ No breakpoints available from CLSI and EUCAST. Values expressed are indicative of the cumulative percentage of isolates inhibited at ≤8 mg/L for comparison purposes. ^e^ Susceptible category for colistin not available for CLSI breakpoints (only intermediate and resistant isolates are available). ^f^ Data for colistin do not include isolates of *Morganella morganii*, *Proteus hauseri*, *Proteus mirabilis*, *Proteus vulgaris*, *Providencia alcalifaciens*, *Providencia rettgeri*, *Providencia* spp., *Providencia stuartii*, and *Serratia marcescens* because of their intrinsic resistance. ^g^ Data for imipenem not available per EUCAST. ^h^ Data for tigecycline do not include isolates of *Morganella morganii*, *Proteus hauseri*, *Proteus mirabilis*, *Proteus vulgaris*, *Providencia alcalifaciens*, *Providencia rettgeri*, *Providencia* spp., and *Providencia stuartii* due to their intrinsic resistance. ^i^ Data for tigecycline were calculated based on FDA approved breakpoints for CLSI. ^j^ EUCAST data for susceptibility to tigecycline are limited to *E. coli* and *C. koseri*; denominator (n): Global: ICU = 6410, non-ICU = 24,016; AfME: ICU = 433, non-ICU = 2032; APAC: ICU = 1037, non-ICU = 5159; Europe: ICU = 3382, non-ICU = 10,890; LATAM: ICU = 901, non-ICU = 3307; North America: ICU = 657, non-ICU = 2628. ICU, intensive care unit; MIC, minimum inhibitory concentration; MIC_90_, minimum inhibitory concentration required to inhibit 90% of the organisms; N, total number of isolates; n, number of isolates from wards; NA, not available.

**Table 3:** In vitro activity of ATM-AVI and comparator agents tested against Enterobacterales isolates across regions stratified by infection sources from 2016 to 2020.

	MIC_90_ (µg/mL) (% S, CLSI/%S, EUCAST ^a^)									
	RTI		UTI		SSTI		BSI		IAI	
**Global (N = 116,602) ^b^**	**n ^c^**		**n ^c^**		**n ^c^**		**n ^c^**		**n ^c^**	
Aztreonam-avibactam ^d^	20,197	0.25 (99.9)	22,199	0.12 (99.9)	18,117	0.12 (99.9)	18,159	0.12 (99.9)	13,341	0.12 (100)
Aztreonam	20,979	128 (71.7/71.7)	22,875	64 (73.0/73.0)	18,504	64 (76.7/76.7)	18,754	128 (70.6/70.6)	13,842	64 (73.8/73.8)
Amikacin	25,575	8 (95.9/94.1)	27,254	8 (96.7/94.7)	22,040	8 (97.4/95.6)	23,450	8 (96.3/94.2)	16,873	8 (97.5/95.7)
Cefepime	25,574	32 (76.0/78.3)	27,254	32 (75.1/77.6)	22,040	32 (79.3/81.6)	23,450	64 (73.6/75.8)	16,873	32 (77.8/80.1)
Ceftazidime	25,575	64 (73.1/73.1)	27,254	64 (74.0/74.0)	22,040	64 (77.2/77.2)	23,450	64 (72.8/72.8)	16,873	64 (75.4/75.4)
Ceftazidime–avibactam	20,979	0.5 (97.6/97.6)	22,876	0.5 (97.6/97.6)	18,504	0.5 (98.1/98.1)	18,754	0.5 (97.4/97.4)	13,842	0.5 (98.3/98.3)
Ceftriaxone	11,953	32 (69.5/70.9)	12,972	32 (70.7/71.8)	10,880	32 (73.3/74.7)	7935	64 (69.6/70.7)	8257	32 (71.1/72.1)
Ciprofloxacin	13,621	8 (67.1/71.2)	14,281	8 (58.9/62.9)	11,160	8 (66.6/71.0)	15,515	8 (62.9/67.3)	8616	8 (67.3/71.1)
Colistin ^e,f^	17,835	1 (NA/96.4)	18,726	1 (NA/97.7)	14,116	1 (NA/97.0)	16,592	1 (NA/96.9)	12,544	1 (NA/97.6)
Gentamicin	13,622	32 (82.7/81.8)	14,282	32 (79.3/77.9)	11,160	32 (83.0/81.8)	15,515	32 (80.9/79.9)	8616	32 (85.1/84)
Imipenem ^g^	20,979	2 (84.4/NA)	22,876	2 (82.6/NA)	18,504	4 (79.6/NA)	18,754	2 (86.2/NA)	13,842	2 (87.4/NA)
Levofloxacin	25,574	16 (71.9/77.1)	27,254	16 (65.3/70.0)	22,040	16 (70.7/75.7)	23,450	16 (68.0/73.0)	16,873	16 (71.0/75.1)
Meropenem	25,574	0.25 (93.6/95.4)	27,254	0.12 (95.3/96.7)	22,040	0.12 (95.5/97.2)	23,450	0.12 (93.8/95.3)	16,873	0.12 (95.2/96.7)
Piperacillin–tazobactam	25,575	128 (76.5/76.5)	27,254	64 (80.8/80.8)	22,040	64 (82.3/82.3)	23,450	128 (79.4/79.4)	16,873	128 (80.4/80.4)
Tigecycline ^h,i,j^	24,093	1 (97.8/98.0)	23,534	1 (98.6/98.1)	18,467	1 (98.1/98.1)	22,139	1 (98.5/98.3)	15,808	1 (98.3/98.1)
**Africa–Middle East (N = 8990)**										
Aztreonam-avibactam ^d^	1433	0.12 (100)	2118	0.12 (99.9)	2202	0.12 (100)	1458	0.12 (99.9)	1010	0.12 (99.9)
Aztreonam	1433	64 (68.5/68.5)	2118	128 (66.2/66.2)	2202	64 (71.9/71.9)	1458	64 (58.9/58.9)	1010	64 (71.9/71.9)
Amikacin	1555	8 (96.9/95.1)	2268	8 (98.0/95.5)	2390	8 (97.5/95.1)	1565	8 (96.4/93.7)	1151	8 (98.0/96.1)
Cefepime	1555	32 (68.2/71.0)	2268	64 (65.8/68.8)	2390	32 (72.2/74.7)	1565	64 (60.3/63.1)	1151	32 (73.5/76.2)
Ceftazidime	1555	64 (69.8/69.8)	2268	64 (66.2/66.2)	2390	64 (72.0/72.0)	1565	64 (58.7/58.7)	1151	64 (72.6/72.6)
Ceftazidime–avibactam	1433	0.5 (98.3/98.3)	2118	0.5 (96.9/96.9)	2202	0.5 (98.1/98.1)	1458	0.5 (95.9/95.9)	1010	0.5 (98.4/98.4)
Ceftriaxone	645	32 (65.6/66.2)	917	32 (69.9/71.1)	1059	32 (71.4/72.1)	346	64 (58.1/59.5)	475	32 (72.0/73.3)
Ciprofloxacin	910	8 (60.1/66.5)	1351	8 (46.4/51.9)	1331	8 (55.2/62.2)	1219	8 (52.5/59.2)	676	8 (58.7/64.9)
Colistin ^e,f^	1209	1 (NA/97.8)	1820	0.5 (NA/98.1)	1705	1 (NA/97.5)	1284	1 (NA/97.8)	904	1 (NA/98.3)
Gentamicin	910	32 (78.5/76.8)	1351	32 (69.3/68)	1331	32 (75.5/74.2)	1219	32 (72.4/71.3)	676	32 (79/77.7)
Imipenem ^g^	1433	2 (85.8/NA)	2118	2 (85.3/NA)	2202	2 (81.5/NA)	1458	2 (85.5/NA)	1010	2 (87.2/NA)
Levofloxacin	1555	16 (69.6/77.9)	2268	16 (59.1/64.8)	2390	16 (63.5/71.3)	1565	16 (62.6/70.9)	1151	16 (66.5/72.9)
Meropenem	1555	0.12 (94.9/96.5)	2268	0.12 (96.1/97.8)	2390	0.12 (96.9/98.2)	1565	0.25 (93.6/95.3)	1151	0.12 (95.9/97.4)
Piperacillin–tazobactam	1555	128 (77.8/77.8)	2268	64 (78.0/78.0)	2390	32 (82.4/82.4)	1565	128 (74.4/74.4)	1151	64 (81.2/81.2)
Tigecycline ^h,i,j^	1447	1 (97.5/97.1)	2005	1 (98.9/98.3)	1967	1 (98.2/97.2)	1472	1 (98.5/98.0)	1064	1 (98.4/98.4)
**Asia–Pacific (N = 21,653)**										
Aztreonam-avibactam ^d^	4243	0.25 (99.7)	4297	0.25 (99.8)	2666	0.25 (99.7)	2995	0.25 (99.8)	2336	0.25 (99.8)
Aztreonam	5025	128 (66.0/66.0)	4973	128 (66.9/66.9)	3053	128 (71.8/71.8)	3590	128 (62.8/62.8)	2837	128 (67.3/67.3)
Amikacin	5525	8 (92.4/90.7)	5339	8 (93.3/91.1)	3239	8 (95.1/93.2)	4247	8 (93.0/91.2)	3182	8 (96.0/94.3)
Cefepime	5524	64 (69.1/71.9)	5339	64 (68.2/71.3)	3239	32 (73.0/75.6)	4247	64 (65.6/68.3)	3182	32 (72.0/74.8)
Ceftazidime	5525	128 (65.8/65.8)	5339	128 (67.9/67.9)	3239	128 (70.4/70.4)	4247	128 (66.7/66.7)	3182	128 (69.3/69.3)
Ceftazidime–avibactam	5025	1 (95.2/95.2)	4973	1 (94.7/94.7)	3053	1 (95.8/95.8)	3590	1 (93.9/93.9)	2837	0.5 (96.8/96.8)
Ceftriaxone	2200	32 (61.8/63.2)	2064	32 (63.6/65.1)	1426	32 (63.2/64.8)	1315	64 (65.2/66.1)	1370	32 (61.4/62.9)
Ciprofloxacin	3324	8 (57.9/63.8)	3274	8 (48.4/53.4)	1813	8 (60.0/65.8)	2932	8 (50.9/56.8)	1812	8 (58.6/64)
Colistin ^e,f^	4375	1 (NA/95.3)	4105	1 (NA/97.3)	2324	1 (NA/95.7)	3207	1 (NA/95.6)	2563	1 (NA/95.7)
Gentamicin	3325	32 (78.7/77.7)	3275	32 (72.6/71)	1813	32 (78.5/77.1)	2932	32 (74.3/73.1)	1812	32 (80.7/79.5)
Imipenem ^g^	5025	4 (81.7/NA)	4973	4 (79.7/NA)	3053	4 (76.3/NA)	3590	4 (83.2/NA)	2837	2 (86.0/NA)
Levofloxacin	5524	16 (62.6/69.2)	5339	16 (55.4/60.5)	3239	16 (63.0/69.1)	4247	16 (58.3/64.0)	3182	16 (63.0/69.0)
Meropenem	5524	2 (89.9/91.3)	5339	0.25 (92.2/93.3)	3239	0.25 (93/94.3)	4247	0.5 (91.2/91.9)	3182	0.25 (94.3/95.2)
Piperacillin–tazobactam	5525	128 (72.7/72.7)	5339	128 (78.9/78.9)	3239	128 (81/81)	4247	128 (79.1/79.1)	3182	128 (80.2/80.2)
Tigecycline ^h,i,j^	5231	2 (96.8/95.3)	4556	1 (98.2/95.8)	2632	1 (97.5/96.5)	4001	1 (98.3/96.3)	2957	1 (98.2/96.3)
**Europe (N = 55,919)**										
Aztreonam-avibactam ^d^	9864	0.25 (99.9)	9238	0.12 (100)	8802	0.12 (100)	8206	0.12 (100)	6411	0.12 (100)
Aztreonam	9864	128 (74.1/74.1)	9238	64 (75.4/75.4)	8802	64 (79.7/79.7)	8206	64 (75.5/75.5)	6411	64 (78.2/78.2)
Amikacin	12,770	4 (96.7/94.9)	12,086	4 (97.3/95.6)	11,008	4 (97.8/96.3)	10,938	8 (97.3/95.2)	8116	4 (97.9/96.2)
Cefepime	12,770	32 (78.7/80.8)	12,086	32 (77.9/80.2)	11,008	32 (82.9/85.0)	10,938	32 (77.8/79.8)	8116	32 (82.1/84.2)
Ceftazidime	12,770	64 (75.6/75.6)	12,086	32 (75.9/75.9)	11,008	32 (80.3/80.3)	10,938	32 (76.4/76.4)	8116	32 (78.9/78.9)
Ceftazidime–avibactam	9864	0.5 (98.1/98.1)	9238	0.5 (98.5/98.5)	8802	0.5 (99.0/99.0)	8206	0.5 (98.6/98.6)	6411	0.5 (98.8/98.8)
Ceftriaxone	6733	32 (71.0/72.5)	6790	32 (71.6/72.6)	5886	32 (76.4/77.7)	4099	64 (72/73.2)	4242	32 (75.7/76.6)
Ciprofloxacin	6037	8 (71.9/75.0)	5296	8 (65.3/68.5)	5122	8 (71.7/74.8)	6839	8 (69.0/72.3)	3874	8 (73.4/76.3)
Colistin ^e,f^	8335	1 (NA/96.7)	7398	0.5 (NA/97.9)	6738	0.5 (NA/97.6)	7273	0.5 (NA/97.6)	5797	0.5 (NA/98.2)
Gentamicin	6037	32 (85.0/84.3)	5296	32 (83.6/82.1)	5122	32 (86.5/85.4)	6839	32 (83.9/83.0)	3874	16 (88.7/87.8)
Imipenem ^g^	9864	2 (84.9/NA)	9238	2 (82.7/NA)	8802	2 (80.6/NA)	8206	2 (87.1/NA)	6411	2 (87.8/NA)
Levofloxacin	12,770	16 (75.1/79.6)	12,086	16 (70.1/74.5)	11,008	16 (74.5/78.7)	10,938	16 (71.4/75.6)	8116	16 (77.0/80.0)
Meropenem	12,770	0.25 (94.5/96.4)	12,086	0.12 (96.2/97.6)	11,008	0.12 (95.8/97.8)	10,938	0.12 (94.5/96.2)	8116	0.12 (95.6/97.2)
Piperacillin–tazobactam	12,770	128 (76.4/76.4)	12,086	64 (80.7/80.7)	11,008	64 (82.2/82.2)	10,938	128 (79.0/79.0)	8116	128 (80.6/80.6)
Tigecycline ^h,i,j^	11,994	1 (98.2/98.6)	10,422	1 (98.5/99.0)	9333	1 (98.3/98.8)	10,331	1 (98.3/98.9)	7602	1 (98.3/98.7)
**Latin America** **(N = 16,501)**										
Aztreonam-avibactam ^d^	2369	0.25 (100)	3973	0.12 (100)	2538	0.12 (100)	3021	0.25 (100)	2358	0.12 (100)
Aztreonam	2369	128 (66.1/66.1)	3973	128 (70.4/70.4)	2538	128 (66.7/66.7)	3021	128 (60.3/60.3)	2358	128 (64.3/64.3)
Amikacin	2714	8 (95.8/93.3)	4371	8 (96.2/93.6)	2926	8 (95.9/92.9)	3556	8 (94.3/91.1)	2814	8 (96.8/93.8)
Cefepime	2714	64 (68.1/70.5)	4371	32 (69.9/72.6)	2926	32 (67.6/70.4)	3556	64 (62.9/65.8)	2814	64 (66/68.9)
Ceftazidime	2714	64 (67.1/67.1)	4371	64 (71.4/71.4)	2926	64 (67.1/67.1)	3556	128 (63.4/63.4)	2814	64 (66.9/66.9)
Ceftazidime–avibactam	2369	1 (98.2/98.2)	3973	0.5 (98.1/98.1)	2538	0.5 (96.7/96.7)	3021	1 (97.0/97.0)	2358	0.5 (98.2/98.2)
Ceftriaxone	1190	32 (64.6/65.3)	1996	32 (66.8/67.8)	1500	32 (62.4/64.3)	1327	64 (60.2/60.7)	1472	64 (60.0/60.3)
Ciprofloxacin	1524	8 (58.3/62.3)	2375	8 (51.5/55.4)	1426	8 (52.7/58.4)	2229	8 (53.1/58.3)	1342	8 (56.3/60.4)
Colistin ^e,f^	2003	1 (NA/95.6)	3316	0.5 (NA/97.4)	1950	1 (NA/95.5)	2614	1 (NA/95.0)	2148	0.5 (NA/97.4)
Gentamicin	1524	32 (75.5/74.6)	2375	32 (74.7/73.2)	1426	32 (71.9/70.6)	2229	32 (74.3/73.1)	1342	32 (77.8/76.5)
Imipenem ^g^	2369	4 (84.0/NA)	3973	2 (82.7/NA)	2538	4 (77.0/NA)	3021	4 (82.8/NA)	2358	2 (86.3/NA)
Levofloxacin	2714	16 (65.7/71.8)	4371	16 (57.2/62.2)	2926	16 (58.7/65.0)	3556	16 (61.8/68.4)	2814	16 (58.9/63.9)
Meropenem	2714	0.25 (91.6/93.8)	4371	0.12 (93.8/95.9)	2926	0.25 (92.8/95.4)	3556	2 (89.8/93.0)	2814	0.25 (93.3/95.7)
Piperacillin–tazobactam	2714	128 (75.8/75.8)	4371	128 (78.6/78.6)	2926	128 (76.7/76.7)	3556	128 (73.7/73.7)	2814	128 (76.1/76.1)
Tigecycline ^h,i,j^	2550	1 (98.0/98.5)	3797	1 (99.0/98.8)	2447	1 (98.3/97.8)	3373	1 (98.5/98.4)	2644	1 (98.5/98.2)
**North America (N = 13,539)**										
Aztreonam-avibactam ^d^	2288	0.25 (99.9)	2573	0.12 (99.9)	1909	0.12 (99.8)	2479	0.12 (100)	1226	0.12 (100)
Aztreonam	2288	32 (81.9/81.9)	2573	16 (86.2/86.2)	1909	8 (88.9/88.9)	2479	32 (85.2/85.2)	1226	16 (85.9/85.9)
Amikacin	3011	4 (98.9/97.3)	3190	4 (99.4/98.6)	2477	4 (99.8/98.9)	3144	4 (99.4/98.4)	1610	4 (99.4/98.9)
Cefepime	3011	4 (88.2/90.3)	3190	4 (89.7/91.3)	2477	1 (91.8/93.9)	3144	8 (88.4/89.8)	1610	2 (90.8/92.7)
Ceftazidime	3011	32 (83.3/83.3)	3190	16 (86.6/86.6)	2477	8 (89.5/89.5)	3144	16 (86.0/86.0)	1610	16 (86.8/86.8)
Ceftazidime–avibactam	2288	0.5 (99.7/99.7)	2574	0.5 (99.7/99.7)	1909	0.5 (99.5/99.5)	2479	0.25 (99.9/99.9)	1226	0.5 (99.8/99.8)
Ceftriaxone	1185	32 (81.9/83.6)	1205	32 (84.6/86.1)	1009	8 (87.0/88.8)	848	32 (84.4/85.9)	698	32 (85.1/86.5)
Ciprofloxacin	1826	8 (78.5/81.5)	1985	8 (76.6/80.0)	1468	4 (81.3/84.7)	2296	8 (75.2/79.1)	912	8 (81.4/83.7)
Colistin ^e,f^	1913	0.5 (NA/98.0)	2087	0.5 (NA/98.2)	1399	0.5 (NA/97.8)	2214	0.5 (NA/98.3)	1132	0.5 (NA/98.5)
Gentamicin	1826	4 (90.5/89.5)	1985	2 (91.2/90.2)	1468	2 (93.6/92.7)	2296	2 (91.4/90.6)	912	2 (93.8/93.1)
Imipenem ^g^	2288	2 (87.6/NA)	2574	2 (85.6/NA)	1909	2 (81.5/NA)	2479	1 (91.9/NA)	1226	1 (91.0/NA)
Levofloxacin	3011	8 (82.0/85.4)	3190	16 (79.4/82.9)	2477	4 (84.7/87.7)	3144	16 (78.8/82.3)	1610	16 (81.2/83.5)
Meropenem	3011	0.12 (97.9/98.9)	3190	0.12 (98.8/99.4)	2477	0.12 (98.6/99.5)	3144	0.12 (99.2/99.6)	1610	0.12 (97.8/99.1)
Piperacillin–tazobactam	3011	32 (84.4/84.4)	3190	16 (89.8/89.8)	2477	8 (90.8/90.8)	3144	16 (89.9/89.9)	1610	16 (86.8/86.8)
Tigecycline ^h,i,j^	2871	1 (97.9/99.1)	2754	1 (98.6/98.1)	2088	1 (98.0/98.9)	2962	1 (99.0/99.4)	1541	1 (98.7/98.6)

^a^ Data includes percentage isolates susceptible at increased exposure. ^b^ Includes *Enterobacter cloacae*, *Enterobacter hormaechi*, *Enterobacter kobei*, *Enterobacter ludwigii*, *Enterobacter asburiae*, *Escherichia coli*, *Klebsiella pneumoniae*, *Klebsiella oxytoca*, *Klebsiella aerogenes*, *Citrobacter koseri*, *Citrobacter freundii*, *Morganella morganii*, *Serratia marcescens*, *Proteus mirabilis*, *Citrobacter amalonaticus*, *Citrobacter braakii*, *Citrobacter farmer*, *Citrobacter* spp., *Enterobacter bugandensis*, *Enterobacter xiangfangensis*, *Klebsiella variicola*, *Proteus hauseri*, *Proteus vulgaris*, *Providencia alcalifaciens*, *Providencia rettgeri*, *Providencia* spp., *Providencia stuartii*, *Raoultella ornithinolytica*. ^c^ Not all drugs in the panel were tested every year. ^d^ No breakpoints available from CLSI and EUCAST. Values expressed are indicative of the cumulative percentage of isolates inhibited at ≤8 mg/L for comparison purposes. ^e^ Susceptible category for colistin not available for CLSI breakpoints (only intermediate and resistant isolates are available). ^f^ Data for colistin do not include isolates of *Morganella morganii*, *Proteus hauseri*, *Proteus mirabilis*, *Proteus vulgaris*, *Providencia alcalifaciens*, *Providencia rettgeri*, *Providencia* spp., *Providencia stuartii*, and *Serratia marcescens* because of their intrinsic resistance. ^g^ Data for imipenem not available per EUCAST. ^h^ Data for tigecycline do not include isolates of *Morganella morganii*, *Proteus hauseri*, *Proteus mirabilis*, *Proteus vulgaris*, *Providencia alcalifaciens*, *Providencia rettgeri*, *Providencia* spp., and *Providencia stuartii* due to their intrinsic resistance. ^i^ Data for tigecycline were calculated based on FDA-approved breakpoints for CLSI. ^j^ EUCAST data for susceptibility to tigecycline are limited to *E. coli* and *C. koseri*; denominator (n): Global: RTI = 4773, UTI = 10,079, SSTI = 6602, BSI = 9213, IAI = 7414; AfME: RTI = 241, UTI = 942, SSTI = 778, BSI = 511, IAI = 544; APAC: RTI = 899, UTI = 2329, SSTI = 997, BSI = 1837, IAI = 1384; Europe: RTI = 2649, UTI = 4055, SSTI = 3236, BSI = 4370, IAI = 3474; LATAM: RTI = 406, UTI = 1784, SSTI = 935, BSI = 1150, IAI = 1350; North America: RTI = 578, UTI = 969, SSTI = 656, BSI = 1345, IAI = 662. BSI, bloodstream infections; CAZ-AVI, ceftazidime–avibactam; IAI, intra-abdominal infection; MIC, minimum inhibitory concentration; N, total number of isolates; n, number of isolates from infection sources; NA, not available; RTI, respiratory tract infection; SSTI, skin and soft tissue infection; UTI, urinary tract infection.

**Table 4:** In vitro activity of ATM-AVI and comparator agents tested against resistance phenotypes of Enterobacterales isolates collected globally stratified by wards from 2016 to 2020.

	ICU				Non-ICU			
	CLSI		EUCAST		CLSI		EUCAST	
All Enterobacterales (N = 116,602) ^a^	n ^b^	MIC_90_ (µg/mL) (% S)	n ^b^	MIC_90_ (mg/L) (% S ^c^)	n ^b^	MIC_90_ (µg/mL) (% S)	n ^b^	MIC_90_ (mg/L) (% S ^c^)
**MDR (CLSI/EUCAST, N = 36,305/39,700)**								
Aztreonam-avibactam ^d^	8289	0.5 (99.7)	8828	0.5 (99.7)	19,564	0.5 (99.8)	21,258	0.5 (99.8)
Aztreonam	8673	256 (20.3)	9232	256 (24.2)	20,599	128 (28.2)	22,380	128 (32.9)
Amikacin	9202	128 (84.6)	9857	64 (80)	21,691	16 (91.6)	23,771	16 (87.7)
Cefepime	9202	64 (27.4)	9857	64 (36.1)	21,691	64 (34.7)	23,771	64 (44.2)
Ceftazidime	9202	256 (21.2)	9857	256 (24.6)	21,691	256 (29.6)	23,771	128 (33.9)
Ceftazidime–avibactam	8673	4 (90.8)	9232	4 (91.3)	20,600	2 (94.9)	22,381	1 (95.3)
Ceftriaxone	2730	64 (7.8)	3005	64 (13.2)	7054	64 (8.7)	7950	64 (14.8)
Ciprofloxacin	6472	8 (24)	6852	8 (32.4)	14,637	8 (24.2)	15,821	8 (32.7)
Colistin ^e,f^	7947	1 (NA)	8462	1 (93)	18,505	1 (NA)	20,142	1 (95.4)
Gentamicin	6472	32 (51.3)	6852	32 (52.3)	14,637	32 (57.5)	15,821	32 (58.2)
Imipenem ^g^	8673	16 (70.0)	9232	16 (NA)	20,600	8 (77.5)	22,381	8 (NA)
Levofloxacin	9202	16 (31.6)	9857	16 (43.1)	21,691	16 (30.5)	23,771	16 (41.1)
Meropenem	9202	32 (75.6)	9857	32 (83)	21,691	8 (85.8)	23,771	8 (91.1)
Piperacillin–tazobactam	9202	256 (35.5)	9857	256 (37.6)	21,691	128 (48.3)	23,771	128 (50.1)
Tigecycline ^h,i,j^	8647	2 (96.1)	9270	2 (96.5)	19,821	1 (96.8)	21,774	1 (95.8)
**ESBL (N = 20,303)**								
Aztreonam-avibactam ^d^	4616	0.25 (99.7)	4616	0.25 (99.7)	10,130	0.25 (99.9)	10,130	0.25 (99.9)
Aztreonam	4769	256 (6.7)	4769	256 (6.7)	10,416	256 (9.6)	10,416	256 (9.6)
Amikacin	5312	128 (82.6)	5312	128 (76.8)	11,837	16 (91.1)	11,837	16 (86.0)
Cefepime	5312	64 (9.3)	5312	64 (13.7)	11,837	64 (11.7)	11,837	64 (17.7)
Ceftazidime	5312	256 (11.7)	5312	256 (11.7)	11,837	256 (16.9)	11,837	256 (16.9)
Ceftazidime–avibactam	4769	128 (87.7)	4769	128 (87.7)	10,416	2 (93.2)	10,416	2 (93.2)
Ceftriaxone	2322	64 (3.6)	2322	64 (4.7)	6308	64 (3.8)	6308	64 (5.3)
Ciprofloxacin	2990	8 (9.7)	2990	8 (15.3)	5529	8 (10.7)	5529	8 (16.1)
Colistin ^e,f^	4622	1 (NA)	4622	1 (93.3)	10,054	1 (NA)	10,054	1 (96.2)
Gentamicin	2990	32 (39.8)	2990	32 (38.9)	5529	32 (47.6)	5529	32 (46.8)
Imipenem ^g^	4769	16 (68.0)	4769	16 (NA)	10,416	8 (80.4)	10,416	8 (NA)
Levofloxacin	5312	16 (22.2)	5312	16 (32.6)	11,837	16 (22.8)	11,837	16 (31.3)
Meropenem	5312	32 (72.2)	5312	32 (79.2)	11,837	16 (84.4)	11,837	16 (89.6)
Piperacillin–tazobactam	5312	256 (38.2)	5312	256 (38.2)	11,837	256 (51.1)	11,837	256 (51.1)
Tigecycline ^h,i,j^	5187	2 (96.5)	5187	2 (97.9)	11,507	1 (97.1)	11,507	1 (97.3)
**CRE (CLSI/EUCAST, N = 5576/4388)**								
Aztreonam-avibactam ^d^	1715	1 (99.0)	1401	1 (99.0)	2288	1 (99.4)	1720	1 (99.4)
Aztreonam	1842	256 (8.6)	1517	256 (6.9)	2479	256 (10.0)	1899	256 (8.5)
Amikacin	2058	128 (50.5)	1681	128 (33.4)	2771	128 (60.7)	2120	128 (43.2)
Cefepime	2058	64 (2.2)	1681	64 (2.1)	2771	64 (2.6)	2120	64 (1.9)
Ceftazidime	2058	256 (3.5)	1681	256 (2.6)	2771	256 (4.2)	2120	256 (2.7)
Ceftazidime–avibactam	1842	256 (59.3)	1517	256 (58.1)	2479	256 (61.9)	1899	256 (59.2)
Ceftriaxone	580	64 (0.7)	446	64 (0.2)	904	64 (0.8)	651	64 (0.8)
Ciprofloxacin	1478	8 (5.8)	1235	8 (5.4)	1867	8 (5.9)	1469	8 (5.5)
Colistin ^e,f^	1701	16 (NA)	1414	16 (81.5)	2317	16 (NA)	1796	16 (83.9)
Gentamicin	1478	32 (27.9)	1235	32 (24)	1867	32 (35.7)	1469	32 (31.4)
Imipenem ^g^	1842	16 (1.3)	1517	16 (NA)	2479	16 (3.1)	1899	16 (5.3)
Levofloxacin	2058	16 (8.7)	1681	16 (8.4)	2771	16 (9)	2120	16 (9.3)
Meropenem	2058	32 (0)	1681	32 (0)	2771	32 (0)	2120	32 (0)
Piperacillin–tazobactam	2058	256 (0.8)	1681	256 (0.5)	2771	256 (1.1)	2120	256 (0.5)
Tigecycline ^h,i,j^	1973	2 (93.7)	1621	2 (89.9)	2668	2 (93.7)	2062	2 (81.2)
**MBL-positive (N = 1877)**								
Aztreonam-avibactam ^d^	693	1 (98.7)	693	1 (98.7)	878	1 (99.7)	878	1 (99.7)
Aztreonam	725	256 (15.3)	725	256 (15.3)	900	256 (19.3)	900	256 (19.3)
Amikacin	728	128 (38.6)	728	128 (28.9)	909	128 (55.1)	909	128 (44.0)
Cefepime	728	64 (0.4)	728	64 (0.7)	909	64 (1.4)	909	64 (3.3)
Ceftazidime	728	256 (0.1)	728	256 (0.1)	909	256 (0)	909	256 (0)
Ceftazidime–avibactam	725	256 (2.1)	725	256 (2.1)	900	256 (2.7)	900	256 (2.7)
Ceftriaxone	89	32 (0)	89	32 (0)	195	32 (0)	195	32 (0)
Ciprofloxacin	639	8 (7.7)	639	8 (9.9)	714	8 (6)	714	8 (10.5)
Colistin ^e,f^	631	2 (NA)	631	2 (90.3)	802	1 (NA)	802	1 (91.8)
Gentamicin	639	32 (23.5)	639	32 (21.8)	714	32 (33.1)	714	32 (31.5)
Imipenem ^g^	725	16 (1.4)	725	16 (NA)	900	16 (1.2)	900	16 (NA)
Levofloxacin	728	16 (12.9)	728	16 (18.8)	909	16 (11.2)	909	16 (18.5)
Meropenem	728	32 (3.2)	728	32 (20.5)	909	32 (4.5)	909	32 (26.2)
Piperacillin–tazobactam	728	256 (1.2)	728	256 (1.2)	909	256 (1.1)	909	256 (1.1)
Tigecycline ^h,i,j^	657	2 (94.8)	657	2 (92.0)	824	2 (93.0)	824	2 (92.4)

^a^ Includes *Enterobacter cloacae*, *Enterobacter hormaechi*, *Enterobacter kobei*, *Enterobacter ludwigii*, *Enterobacter asburiae*, *Escherichia coli*, *Klebsiella pneumoniae*, *Klebsiella oxytoca*, *Klebsiella aerogenes*, *Citrobacter koseri*, *Citrobacter freundii*, *Morganella morganii*, *Serratia marcescens*, *Proteus mirabilis*, *Citrobacter amalonaticus*, *Citrobacter braakii*, *Citrobacter farmer*, *Citrobacter* spp., *Enterobacter bugandensis*, *Enterobacter xiangfangensis*, *Klebsiella variicola*, *Proteus hauseri*, *Proteus vulgaris*, *Providencia alcalifaciens*, *Providencia rettgeri*, *Providencia* spp., *Providencia stuartii*, *Raoultella ornithinolytica*. ^b^ Not all drugs in the panel were tested every year. ^c^ Data include percentage isolates susceptible at increased exposure. ^d^ No breakpoints available from CLSI and EUCAST. Values expressed are indicative of the cumulative percentage of isolates inhibited at ≤8 mg/L for comparison purposes. ^e^ Susceptible category for colistin not available for CLSI breakpoints (only intermediate and resistant isolates are available). ^f^ Data for colistin do not include isolates of *Morganella morganii*, *Proteus hauseri*, *Proteus mirabilis*, *Proteus vulgaris*, *Providencia alcalifaciens*, *Providencia rettgeri*, *Providencia* spp., *Providencia stuartii*, and *Serratia marcescens* because of their intrinsic resistance. ^g^ Data for imipenem not available per EUCAST. ^h^ Data for tigecycline do not include isolates of *Morganella morganii*, *Proteus hauseri*, *Proteus mirabilis*, *Proteus vulgaris*, *Providencia alcalifaciens*, *Providencia rettgeri*, *Providencia* spp., and *Providencia stuartii* due to their intrinsic resistance. ^i^ Data for tigecycline were calculated based on FDA-approved breakpoints for CLSI. ^j^ EUCAST data for susceptibility to tigecycline are limited to *E. coli* and *C. koseri*; denominator (n): MDR: ICU = 2713, non-ICU = 9624; ESBL: ICU = 1463, non-ICU = 4923; CRE: ICU = 99, non-ICU = 181; MBL-positive: ICU = 87, non-ICU = 118. ICU, intensive care unit; MIC, minimum inhibitory concentration; N, total number of isolates; n, number of isolates from wards; NA, not available.

**Table 5:** In vitro activity of ATM-AVI and comparator agents tested against resistance phenotypes of Enterobacterales isolates collected globally stratified by infection sources from 2016 to 2020.

	RTI				UTI				SSTI			
	CLSI		EUCAST		CLSI		EUCAST		CLSI		EUCAST	
All Enterobacterales (N = 116,602) ^a^	n ^b^	MIC_90_ (µg/mL) (% S)	n ^b^	MIC_90_ (mg/L) (% S ^c^)	n ^b^	MIC_90_ (µg/mL) (% S)	n ^b^	MIC_90_ (mg/L) (% S^c^)	n ^b^	MIC_90_ (µg/mL) (% S)	n ^b^	MIC_90_ (mg/L) (% S ^c^)
**MDR (CLSI/EUCAST, N = 36,305/39,700)**												
Aztreonam-avibactam ^d^	6968	0.5 (99.7)	7469	0.5 (99.7)	8127	0.5 (99.8)	8810	0.25 (99.8)	5888	0.5 (99.7)	6364	0.5 (99.8)
Aztreonam	7359	256 (20.9)	7884	256 (25.0)	8547	128 (29.2)	9262	128 (33.5)	6070	128 (30.0)	6562	128 (34.3)
Amikacin	7772	64 (86.9)	8393	32 (82.8)	8955	16 (90.3)	9815	16 (86.4)	6381	16 (91.4)	6927	16 (87.1)
Cefepime	7772	64 (29.9)	8393	64 (38.8)	8955	64 (32.8)	9815	64 (42.5)	6381	64 (35.5)	6927	64 (45.2)
Ceftazidime	7772	256 (22.1)	8393	256 (25.8)	8955	256 (30.3)	9815	256 (34.4)	6381	256 (30.1)	6927	256 (34)
Ceftazidime–avibactam	7359	2 (93.3)	7884	2 (93.7)	8548	2 (93.6)	9263	2 (94.1)	6070	2 (94.2)	6562	2 (94.6)
Ceftriaxone	2709	64 (7.2)	3010	64 (13.0)	2853	64 (7.2)	3257	64 (13.8)	2363	32 (9.8)	2626	32 (17.6)
Ciprofloxacin	5063	8 (24.6)	5383	8 (32.7)	6102	8 (20.6)	6558	8 (28.2)	4018	8 (25.8)	4301	8 (34.4)
Colistin ^e,f^	6734	1 (NA)	7212	1 (93.2)	7530	1 (NA)	8173	1 (95.8)	5195	1 (NA)	5634	1 (94.9)
Gentamicin	5063	32 (54.7)	5383	32 (55.2)	6102	32 (53.6)	6558	32 (54.3)	4018	32 (54.8)	4301	32 (55.2)
Imipenem ^g^	7359	16 (72.8)	7884	16 (NA)	8548	8 (77.1)	9263	8 (NA)	6070	8 (73.2)	6562	8 (NA)
Levofloxacin	7772	16 (31.2)	8393	16 (43.3)	8955	16 (27)	9815	16 (36.8)	6381	16 (31.7)	6927	16 (43.2)
Meropenem	7772	32 (79.3)	8393	16 (85.9)	8955	8 (85.9)	9815	8 (91.0)	6381	8 (84.5)	6927	8 (91.1)
Piperacillin–tazobactam	7772	256 (35.9)	8393	256 (37.3)	8955	128 (50.5)	9815	128 (52.4)	6381	128 (48.1)	6927	128 (49.7)
Tigecycline ^h,i,j^	7337	2 (95.6)	7927	2 (95.7)	7995	1 (97.2)	8790	1 (96.0)	5566	1 (96.4)	6060	1 (96.0)
**ESBL (N = 20,303)**												
Aztreonam-avibactam ^d^	3875	0.25 (99.8)	3875	0.25 (99.8)	4309	0.25 (99.8)	4309	0.25 (99.8)	3118	0.25 (99.8)	3118	0.25 (99.8)
Aztreonam	3967	256 (7.8)	3967	256 (7.8)	4486	256 (9.9)	4486	256 (9.9)	3186	256 (11.2)	3186	256 (11.2)
Amikacin	4463	64 (86.2)	4463	64 (80.6)	5093	32 (89.8)	5093	32 (84.7)	3508	16 (90.5)	3508	16 (85.2)
Cefepime	4463	64 (11.2)	4463	64 (16.3)	5093	64 (11.0)	5093	64 (17.6)	3508	64 (12.9)	3508	64 (19.0)
Ceftazidime	4463	256 (12.8)	4463	256 (12.8)	5093	256 (17.6)	5093	256 (17.6)	3508	256 (17.2)	3508	256 (17.2)
Ceftazidime–avibactam	3967	4 (90.9)	3967	4 (90.9)	4486	2 (92.3)	4486	2 (92.3)	3186	2 (91.9)	3186	2 (91.9)
Ceftriaxone	2390	64 (4.1)	2390	64 (5.4)	2621	64 (3.4)	2621	64 (5.0)	2028	64 (4.5)	2028	64 (6.9)
Ciprofloxacin	2073	8 (8.6)	2073	8 (14)	2472	8 (10.3)	2472	8 (14.9)	1480	8 (10.7)	1480	8 (17.2)
Colistin ^e,f^	3852	1 (NA)	3852	1 (93.8)	4320	1 (NA)	4320	1 (96.1)	3024	1 (NA)	3024	1 (95.9)
Gentamicin	2073	32 (42.6)	2073	32 (41.6)	2472	32 (46.1)	2472	32 (45.2)	1480	32 (42.0)	1480	32 (41.3)
Imipenem ^g^	3967	16 (72.7)	3967	16 (NA)	4486	16 (80.0)	4486	16 (NA)	3186	8 (78)	3186	8 (NA)
Levofloxacin	4463	16 (22.7)	4463	16 (33.6)	5093	16 (20.9)	5093	16 (27.9)	3508	16 (23.1)	3508	16 (33.3)
Meropenem	4463	16 (77.5)	4463	16 (83.6)	5093	16 (84.4)	5093	16 (88.9)	3508	16 (82.3)	3508	16 (89.1)
Piperacillin–tazobactam	4463	256 (39.3)	4463	256 (39.3)	5093	128 (52.6)	5093	128 (52.6)	3508	256 (50.2)	3508	256 (50.2)
Tigecycline ^h,i,j^	4368	2 (96.2)	4368	2 (98.0)	4937	1 (97.6)	4937	1 (97.2)	3356	1 (96.6)	3356	1 (97.5)
**CRE (CLSI/EUCAST, N = 5576/4388)**												
Aztreonam-avibactam ^d^	1200	1 (99.2)	955	1 (99.1)	967	1 (99.2)	749	2 (98.9)	747	1 (99.1)	528	0.5 (99.2)
Aztreonam	1328	256 (7.6)	1071	256 (5.4)	1044	256 (14.6)	820	256 (12.7)	779	256 (11.9)	560	256 (10.2)
Amikacin	1475	128 (53.9)	1190	128 (39.5)	1142	128 (53.7)	892	128 (37.3)	870	128 (61.4)	620	128 (41.5)
Cefepime	1475	64 (3.0)	1190	64 (2.2)	1142	64 (2.5)	892	64 (1.7)	870	64 (2.9)	620	64 (2.4)
Ceftazidime	1475	256 (4.2)	1190	256 (2.9)	1142	256 (3.5)	892	256 (2.4)	870	256 (5.8)	620	256 (3.4)
Ceftazidime–avibactam	1328	256 (66.0)	1071	256 (64.3)	1044	256 (51.8)	820	256 (48.8)	779	256 (59.1)	560	256 (54.6)
Ceftriaxone	465	64 (0.9)	357	64 (0.6)	331	64 (1.2)	234	64 (0.9)	303	64 (1.0)	198	64 (1.0)
Ciprofloxacin	1010	8 (4.2)	833	8 (3.8)	811	8 (4.3)	658	8 (3.3)	567	8 (7.2)	422	8 (7.8)
Colistin ^e,f^	1248	16 (NA)	1015	16 (82.0)	931	16 (NA)	743	16 (83.2)	722	8 (NA)	519	16 (83.2)
Gentamicin	1010	32 (31.5)	833	32 (27.1)	811	32 (26.4)	658	32 (21.7)	567	32 (36.0)	422	32 (30.3)
Imipenem ^g^	1328	16 (1.9)	1071	16 (4.8)	1044	16 (3.3)	820	16 (6.5)	779	16 (3.2)	560	16 (NA)
Levofloxacin	1475	16 (6.9)	1190	16 (7.0)	1142	16 (7.5)	892	16 (8.7)	870	16 (11.3)	620	16 (12.6)
Meropenem	1475	32 (0)	1190	32 (0)	1142	32 (0)	892	32 (0)	870	32 (0)	620	32 (0)
Piperacillin–tazobactam	1475	256 (1.3)	1190	256 (0.7)	1142	256 (1.3)	892	256 (0.7)	870	256 (1.2)	620	256 (0.3)
Tigecycline ^h,i,j^	1448	2 (93.1)	1177	2 (76.4)	1049	2 (94.7)	827	2 (87.3)	825	2 (92.6)	589	2 (83.7)
**MBL-positive (N = 1877)**												
Aztreonam-avibactam ^d^	423	0.5 (99.5)	423	0.5 (99.5)	468	2 (98.7)	468	2 (98.7)	316	0.5 (99.7)	316	0.5 (99.7)
Aztreonam	436	256 (15.1)	436	256 (15.1)	483	256 (24.4)	483	256 (24.4)	325	256 (19.7)	325	256 (19.7)
Amikacin	436	128 (45.9)	436	128 (35.3)	487	128 (45.2)	487	128 (37.0)	327	128 (53.8)	327	128 (39.5)
Cefepime	436	64 (0)	436	64 (0.7)	487	64 (1.2)	487	64 (3.5)	327	64 (0.9)	327	64 (2.1)
Ceftazidime	436	256 (0)	436	256 (0)	487	256 (0)	487	256 (0)	327	256 (0)	327	256 (0)
Ceftazidime–avibactam	436	256 (2.1)	436	256 (2.1)	483	256 (1.5)	483	256 (1.5)	325	256 (4.9)	325	256 (4.9)
Ceftriaxone	78	32 (0)	78	32 (0)	74	32 (0)	74	32 (0)	78	32 (0)	78	32 (0)
Ciprofloxacin	358	8 (5.6)	358	8 (8.7)	413	8 (5.1)	413	8 (8.0)	249	8 (6.4)	249	8 (10.0)
Colistin ^e,f^	398	4 (NA)	398	4 (89.7)	403	4 (NA)	403	4 (89.8)	284	1 (NA)	284	1 (94.7)
Gentamicin	358	32 (27.7)	358	32 (26.5)	413	32 (22.5)	413	32 (21.1)	249	32 (32.5)	249	32 (31.3)
Imipenem ^g^	436	16 (1.6)	436	16 (10.1)	483	16 (0.8)	483	16 (NA)	325	16 (1.5)	325	16 (NA)
Levofloxacin	436	16 (11.0)	436	16 (16.7)	487	16 (9.5)	487	16 (17.0)	327	16 (12.5)	327	16 (21.4)
Meropenem	436	32 (4.6)	436	32 (22.3)	487	32 (3.9)	487	32 (22.0)	327	32 (3.7)	327	32 (28.4)
Piperacillin–tazobactam	436	256 (1.8)	436	256 (1.8)	487	256 (1.4)	487	256 (1.4)	327	256 (0.9)	327	256 (0.9)
Tigecycline ^h,i,j^	410	2 (94.2)	410	2 (91.7)	416	2 (94.7)	416	2 (91.8)	289	2 (93.1)	289	2 (95.5)
^a^ Includes *Enterobacter cloacae*, *Enterobacter hormaechi*, *Enterobacter kobei*, *Enterobacter ludwigii*, *Enterobacter asburiae*, *Escherichia coli*, *Klebsiella pneumoniae*, *Klebsiella oxytoca*, *Klebsiella aerogenes*, *Citrobacter koseri*, *Citrobacter freundii*, *Morganella morganii*, *Serratia marcescens*, *Proteus mirabilis, Citrobacter amalonaticus*, *Citrobacter braakii*, *Citrobacter farmer*, *Citrobacter* spp., *Enterobacter bugandensis*, *Enterobacter xiangfangensis*, *Klebsiella variicola*, *Proteus hauseri*, *Proteus vulgaris*, *Providencia alcalifaciens*, *Providencia rettgeri*, *Providencia* spp., *Providencia stuartii*, *Raoultella ornithinolytica*. ^b^ Not all drugs in the panel were tested every year. ^c^ Data include percentage isolates susceptible at increased exposure. ^d^ No breakpoints available from CLSI and EUCAST. Values expressed are indicative of the cumulative percentage of isolates inhibited at ≤8 mg/L for comparison purposes. ^e^ Susceptible category for colistin not available for CLSI breakpoints (only intermediate and resistant isolates are available). ^f^ Data for colistin do not include isolates of *Morganella morganii*, *Proteus hauseri*, *Proteus mirabilis*, *Proteus vulgaris*, *Providencia alcalifaciens*, *Providencia rettgeri*, *Providencia* spp., *Providencia stuartii*, and *Serratia marcescens* because of their intrinsic resistance. ^g^ Data for imipenem not available per EUCAST. ^h^ Data for tigecycline do not include isolates of *Morganella morganii*, *Proteus hauseri*, *Proteus mirabilis*, *Proteus vulgaris*, *Providencia alcalifaciens*, *Providencia rettgeri*, *Providencia* spp., and *Providencia stuartii* due to their intrinsic resistance. ^i^ Data for tigecycline were calculated based on FDA-approved breakpoints for CLSI. ^j^ EUCAST data for susceptibility to tigecycline are limited to *E. coli* and *C. Koseri*; denominator (n): MDR: RTI = 1940, UTI = 4113, SSTI = 2498; ESBL: RTI = 1023, UTI = 2179, SSTI = 1432; CRE: RTI = 55, UTI = 102, SSTI = 49; MBL-positive: RTI = 36, UTI = 85, SSTI = 44. MIC, minimum inhibitory concentration; N, total number of isolates; n, number of isolates from infection sources; NA, not available; RTI, respiratory tract infection; SSTI, skin and soft tissue infection; UTI, urinary tract infection.												
	**BSI**						**IAI**					
	**CLSI**			**EUCAST**			**CLSI**			**EUCAST**		
**All Enterobacterales** **(N = 116,602) ^a^**	**n ^b^**	**MIC_90_ (µg/mL)** **(% S)**		**n ^b^**	**MIC_90_ (mg/L)** **(% S ^c^)**		**n ^b^**	**MIC_90_ (µg/mL)** **(% S)**		**n ^b^**	**MIC_90_ (mg/L)** **(% S ^c^)**	
**MDR (CLSI/EUCAST, N = 36,305/39,700)**												
Aztreonam-avibactam ^d^	7004	0.5 (99.8)		7600	0.25 (99.8)		4574	0.5 (99.9)		4994	0.5 (99.9)	
Aztreonam	7364	128 (25.9)		7990	128 (31.1)		4837	128 (26.6)		5279	128 (31.5)	
Amikacin	7836	32 (89.2)		8651	16 (85.0)		5212	16 (92.3)		5728	16 (88.1)	
Cefepime	7836	64 (30.4)		8651	64 (39.6)		5212	64 (36.4)		5728	64 (46.3)	
Ceftazidime	7836	256 (27.5)		8651	128 (32.1)		5212	256 (29.9)		5728	256 (34.5)	
Ceftazidime–avibactam	7364	2 (93.4)		7990	2 (93.9)		4837	2 (95.2)		5279	2 (95.6)	
Ceftriaxone	1529	64 (8.6)		1812	64 (13.5)		1907	64 (10.2)		2131	64 (16.2)	
Ciprofloxacin	6307	8 (22.7)		6839	8 (31.9)		3305	8 (29.3)		3597	8 (38.2)	
Colistin ^e,f^	6821	1 (NA)		7403	1 (94.8)		4536	1 (NA)		4976	1 (95.4)	
Gentamicin	6307	32 (54.5)		6839	32 (56.0)		3305	32 (62.5)		3597	32 (63.3)	
Imipenem ^g^	7364	16 (76.2)		7990	16 (NA)		4837	8 (79.6)		5279	8 (NA)	
Levofloxacin	7836	16 (29.8)		8651	16 (40.9)		5212	16 (34.3)		5728	16 (44)	
Meropenem	7836	32 (81.4)		8651	16 (87.3)		5212	16 (84.6)		5728	8 (90.4)	
Piperacillin–tazobactam	7836	128 (46.4)		8651	128 (48.8)		5212	256 (46)		5728	256 (48.2)	
Tigecycline ^h,i,j^	7414	2 (96.9)		8196	1 (96.5)		4940	1 (96.7)		5453	1 (95.8)	
**ESBL (N = 20,303)**												
Aztreonam-avibactam ^b^	3433	0.25 (99.9)		3433	0.25 (99.9)		2397	0.25 (99.9)		2397	0.25 (99.9)	
Aztreonam	3560	256 (6.0)		3560	256 (6.0)		2458	256 (10.0)		2458	256 (10.0)	
Amikacin	4227	32 (88.5)		4227	32 (83.1)		2855	16 (91.2)		2855	16 (85.9)	
Cefepime	4227	64 (7.1)		4227	64 (12.2)		2855	64 (13.7)		2855	64 (19.4)	
Ceftazidime	4227	256 (14.5)		4227	256 (14.5)		2855	256 (18.1)		2855	256 (18.1)	
Ceftazidime–avibactam	3560	4 (90.4)		3560	4 (90.4)		2458	2 (93.7)		2458	2 (93.7)	
Ceftriaxone	1530	64 (2.4)		1530	64 (3.5)		1581	64 (3.9)		1581	64 (4.9)	
Ciprofloxacin	2697	8 (10.8)		2697	8 (15.9)		1274	8 (12.6)		1274	8 (18.3)	
Colistin ^e,f^	3469	1 (NA)		3469	1 (95.2)		2411	1 (NA)		2411	1 (95.8)	
Gentamicin	2697	32 (44.4)		2697	32 (43.5)		1274	32 (51.3)		1274	32 (50.2)	
Imipenem ^g^	3560	16 (75.8)		3560	16 (NA)		2458	8 (80.2)		2458	8 (NA)	
Levofloxacin	4227	16 (21.2)		4227	16 (30.6)		2855	16 (24.6)		2855	16 (32)	
Meropenem	4227	32 (79.4)		4227	32 (84.8)		2855	16 (83.2)		2855	16 (88.8)	
Piperacillin–tazobactam	4227	128 (48.7)		4227	128 (48.7)		2855	256 (51.5)		2855	256 (51.5)	
Tigecycline ^h,i,j^	4150	2 (97.1)		4150	2 (98.1)		2812	1 (97.0)		2812	1 (97.1)	
**CRE (CLSI/EUCAST, N = 5576/4388)**												
Aztreonam-avibactam ^b^	1080	0.5 (99.3)		882	0.5 (99.4)		549	1 (99.8)		425	1 (99.8)	
Aztreonam	1161	256 (8.4)		959	256 (7.3)		591	256 (6.9)		463	256 (6.1)	
Amikacin	1328	128 (56.3)		1096	128 (37.9)		706	128 (63.2)		551	128 (41.7)	
Cefepime	1328	64 (1.8)		1096	64 (2.0)		706	64 (2.0)		551	64 (1.6)	
Ceftazidime	1328	256 (3.8)		1096	256 (3.2)		706	256 (2.3)		551	256 (1.6)	
Ceftazidime–avibactam	1161	256 (60.6)		959	256 (59.7)		591	256 (66.3)		463	256 (65)	
Ceftriaxone	320	64 (0)		254	64 (0.4)		286	64 (0)		227	64 (0.4)	
Ciprofloxacin	1008	8 (7.2)		842	8 (7.4)		420	8 (7.4)		324	8 (5.9)	
Colistin ^e,f^	1076	16 (NA)		899	16 (83.0)		566	16 (NA)		451	16 (82.0)	
Gentamicin	1008	32 (34.0)		842	32 (30.3)		420	32 (36.7)		324	32 (32.7)	
Imipenem ^g^	1161	16 (1.7)		959	16 (5.2)		591	16 (2.9)		463	16 (4.3)	
Levofloxacin	1328	16 (10.1)		1096	16 (11.3)		706	16 (8.6)		551	16 (8.7)	
Meropenem	1328	32 (0)		1096	32 (0)		706	32 (0)		551	32 (0)	
Piperacillin–tazobactam	1328	256 (0.5)		1096	256 (0.4)		706	256 (0.9)		551	256 (0.7)	
Tigecycline ^h,i,j^	1284	2 (94.1)		1069	2 (89.4)		689	2 (93.2)		545	2 (83.7)	
**MBL-positive (N = 1877)**												
Aztreonam-avibactam ^b^	398	0.5 (99.5)		398	0.5 (99.5)		188	1 (99.5)		188	1 (99.5)	
Aztreonam	426	256 (15.7)		426	256 (15.7)		190	256 (15.8)		190	256 (15.8)	
Amikacin	430	128 (45.1)		430	128 (35.4)		193	128 (59.1)		193	128 (50.3)	
Cefepime	430	64 (0)		430	64 (0.5)		193	64 (3.6)		193	64 (5.2)	
Ceftazidime	430	256 (0)		430	256 (0)		193	256 (0.5)		193	256 (0.5)	
Ceftazidime–avibactam	426	256 (1.2)		426	256 (1.2)		190	256 (2.1)		190	256 (2.1)	
Ceftriaxone	44	32 (0)		44	32 (0)		52	32 (0)		52	32 (0)	
Ciprofloxacin	386	8 (9.3)		386	8 (13.2)		141	8 (7.8)		141	8 (10.6)	
Colistin ^e,f^	372	2 (NA)		372	2 (92.2)		179	8 (NA)		179	8 (89.4)	
Gentamicin	386	32 (28.5)		386	32 (25.4)		141	32 (36.2)		141	32 (33.3)	
Imipenem ^g^	426	16 (0.5)		426	16 (NA)		190	16 (2.1)		190	16 (NA)	
Levofloxacin	430	16 (15.1)		430	16 (24.2)		193	16 (11.4)		193	16 (18.1)	
Meropenem	430	32 (2.3)		430	32 (19.3)		193	32 (5.7)		193	32 (30.1)	
Piperacillin–tazobactam	430	128 (0.7)		430	128 (0.7)		193	256 (0.5)		193	256 (0.5)	
Tigecycline ^h,i,j^	389	2 (95.1)		389	2 (95.7)		183	2 (91.8)		183	2 (87.0)	
^a^ Includes *Enterobacter cloacae*, *Enterobacter hormaechi*, *Enterobacter kobei*, *Enterobacter ludwigii*, *Enterobacter asburiae*, *Escherichia coli*, *Klebsiella pneumoniae*, *Klebsiella oxytoca*, *Klebsiella aerogenes*, *Citrobacter koseri*, *Citrobacter freundii*, *Morganella morganii*, *Serratia marcescens*, *Proteus mirabilis*, *Citrobacter amalonaticus*, *Citrobacter braakii*, *Citrobacter farmer*, *Citrobacter* spp., *Enterobacter bugandensis*, *Enterobacter xiangfangensis*, *Klebsiella variicola*, *Proteus hauseri*, *Proteus vulgaris*, *Providencia alcalifaciens*, *Providencia rettgeri*, *Providencia* spp., *Providencia stuartii*, *Raoultella ornithinolytica*. ^b^ Not all drugs in the panel were tested every year. ^c^ Data include percentage isolates susceptible at increased exposure. ^d^ No breakpoints available from CLSI and EUCAST. Values expressed are indicative of the cumulative percentage of isolates inhibited at ≤8 mg/L for comparison purposes. ^e^ Susceptible category for colistin not available for CLSI breakpoints (only intermediate and resistant isolates are available). ^f^ Data for colistin do not include isolates of *Morganella morganii*, *Proteus hauseri*, *Proteus mirabilis*, *Proteus vulgaris*, *Providencia alcalifaciens*, *Providencia rettgeri*, *Providencia* spp., *Providencia stuartii*, and *Serratia marcescens* because of their intrinsic resistance. ^g^ Data for imipenem not available per EUCAST. ^h^ Data for tigecycline do not include isolates of *Morganella morganii*, *Proteus hauseri*, *Proteus mirabilis*, *Proteus vulgaris*, *Providencia alcalifaciens*, *Providencia rettgeri*, *Providencia* spp., and *Providencia stuartii* due to their intrinsic resistance. ^i^ Data for tigecycline were calculated based on FDA approved breakpoints for CLSI. ^j^ EUCAST data for susceptibility to tigecycline are limited to *E. coli* and *C. koseri*; denominator (n): MDR: BSI = 3665, IAI = 2731; ESBL: BSI = 1711, IAI = 1428; CRE: BSI = 66, IAI = 43; MBL-positive: BSI = 47, IAI = 23. BSI, bloodstream infections; IAI, intra-abdominal infection; MIC, minimum inhibitory concentration; N, total number of isolates; n, number of isolates from infection sources; NA, not available.												

## Data Availability

Data are available in a publicly accessible repository that does not issue DOIs. Publicly available datasets were analyzed in this study. This data can be found here: https://atlas-surveillance.com (accessed on 21 April 2023).

## Supplementary Materials

The following supporting information can be downloaded at: https://www.mdpi.com/article/10.3390/antibiotics12111591/s1, Table S1: Species distribution of Enterobacterales isolates collected globally stratified by wards and infection sources, 2016–2020; Table S2: Percentage frequency distribution at aztreonam-avibactam and aztreonam MICs (µg/mL) for all Enterobacterales collected globally across wards, 2016–2020; Table S3: Percentage frequency distribution at aztreonam-avibactam and aztreonam MICs (µg/mL) for all Enterobacterales collected globally across infection sources, 2016–2020; Table S4: In vitro activity of ATM-AVI and comparator agents tested against multidrug-resistant (MDR) Enterobacterales isolates across regions stratified by wards from 2016 to 2020; Table S5: In vitro activity of ATM-AVI and comparator agents tested against multidrug-resistant (MDR) Enterobacterales isolates across regions stratified by infection sources from 2016 to 2020; Table S6: In vitro activity of ATM-AVI and comparator agents tested against ESBL-producing Enterobacterales isolates across regions stratified by wards from 2016 to 2020; Table S7: In vitro activity of ATM-AVI and comparator agents tested against ESBL-producing Enterobacterales isolates across regions stratified by infection sources from 2016 to 2020; Table S8: In vitro activity of ATM-AVI and comparator agents tested against carbapenem-resistant Enterobacterales (CRE) isolates across regions stratified by wards from 2016 to 2020; Table S9: In vitro activity of ATM-AVI and comparator agents tested against carbapenem-resistant Enterobacterales (CRE) isolates across regions stratified by infection sources from 2016 to 2020; Table S10: In vitro activity of ATM-AVI and comparator agents tested against MBL-positive Enterobacterales isolates across regions stratified by wards from 2016 to 2020; Table S11: In vitro activity of ATM-AVI and comparator agents tested against MBL-positive Enterobacterales isolates across regions stratified by infection sources from 2016 to 2020.
