# Supplementary material for: Antimicrobial Activities of Aztreonam-Avibactam and Comparator Agents against Enterobacterales Analyzed by ICU and Non-ICU Wards, Infection Sources, and Geographic Regions: ATLAS Program 2016–2020
Source: Antibiotics (Basel). 2023 Nov 3;12(11):1591. doi: 10.3390/antibiotics12111591 (PMC10668788; doi:10.3390/antibiotics12111591)
Supplement: Supplementary file 1 [file antibiotics-12-01591-s001.zip › antibiotics-2646985-supplementary.pdf]

antibiotics

MDPI

Table S1. Species distribution of Enterobacterales isolates collected globally stratified by wards and infection sources, 2016-2020.

		Wards n (%)			Infection sources n (%)				
	n[a,b]	ICU n (%)	Non-ICU n (%)	n[a,c]	RTI n (%)	UTI n (%)	SSTI n (%)	BSI n (%)	IAI n (%)
**Enterobacterales (N=116602)[d]**									
Citrobacter amalonaticus	104	19 (18.3)	78 (75.0)	109	7 (6.4)	23 (21.1)	32 (29.4)	12 (11.0)	35 (32.1)
Citrobacter braakii	328	53 (16.2)	247 (75.3)	339	53 (15.6)	62 (18.3)	87 (25.7)	42 (12.4)	95 (28)
Citrobacter koseri	2552	499 (19.6)	1663 (65.2)	2797	592 (21.2)	1027 (36.7)	644 (23.0)	268 (9.6)	215 (7.7)
Citrobacter farmeri	57	7 (12.3)	48 (84.2)	62	2 (3.2)	23 (37.1)	12 (19.4)	2 (3.2)	23 (37.1)
Citrobacter freundii	3343	635 (19)	2315 (69.2)	3603	610 (16.9)	1164 (32.3)	742 (20.6)	388 (10.8)	681 (18.9)
Citrobacter spp.	37	10 (27.0)	22 (59.5)	43	9 (20.9)	10 (23.3)	9 (20.9)	6 (14.0)	9 (20.9)
Enterobacter asburiae	926	259 (28.0)	571 (61.7)	981	296 (30.2)	150 (15.3)	160 (16.3)	204 (20.8)	150 (15.3)
Enterobacter bugandensis	290	86 (29.7)	173 (59.7)	313	99 (31.6)	29 (9.3)	62 (19.8)	73 (23.3)	49 (15.7)
Enterobacter cloacae	10554	2675 (25.3)	6778 (64.2)	11295	2779 (24.6)	1798 (15.9)	3031 (26.8)	1947 (17.2)	1535 (13.6)
Enterobacter hormaechi	49	10 (20.4)	35 (71.4)	53	17 (32.1)	7 (13.2)	9 (17.0)	8 (15.1)	12 (22.6)
Enterobacter kobei	409	101 (24.7)	258 (63.1)	435	113 (26.0)	75 (17.2)	71 (16.3)	92 (21.1)	75 (17.2)
Enterobacter ludwigii	129	33 (25.6)	86 (66.7)	137	30 (21.9)	10 (7.3)	31 (22.6)	19 (13.9)	44 (32.1)
Enterobacter xiangfangensis	173	51 (29.5)	111 (64.2)	179	54 (30.2)	24 (13.4)	54 (30.2)	26 (14.5)	21 (11.7)
Escherichia coli	33446	5911 (17.7)	22353 (66.8)	35544	4181 (11.8)	9052 (25.5)	5958 (16.8)	8945 (25.2)	7199 (20.3)
Klebsiella aerogenes	4190	1214 (29.0)	2469 (58.9)	4501	1541 (34.2)	990 (22.0)	735 (16.3)	670 (14.9)	473 (10.5)
Klebsiella pneumoniae	29601	8368 (28.3)	17868 (60.4)	31320	9190 (29.3)	7063 (22.6)	4347 (13.9)	6817 (21.8)	3677 (11.7)
Klebsiella oxytoca	4928	1265 (25.7)	3083 (62.6)	5286	1503 (28.4)	979 (18.5)	1016 (19.2)	938 (17.7)	785 (14.9)
Klebsiella variicola	1706	389 (22.8)	1096 (64.2)	1802	503 (27.9)	348 (19.3)	203 (11.3)	440 (24.4)	293 (16.3)
Morganella morganii	2676	447 (16.7)	1942 (72.57)	2845	377 (13.3)	848 (29.8)	938 (33.0)	331 (11.6)	351 (12.3)
Proteus hauseri	377	49 (13.0)	284 (75.3)	402	38 (9.5)	129 (32.1)	170 (42.3)	25 (6.2)	40 (10.0)
Proteus mirabilis	3919	677 (17.3)	2716 (69.3)	4194	580 (13.8)	1334 (31.8)	1307 (31.2)	604 (14.4)	369 (8.8)
Proteus vulgaris	1404	206	1014	1479	179	464	551	95	190

		(14.7)	(72.2)		(12.1)	(31.4)	(37.3)	(6.4)	(12.8)
Providencia alcalifaciens	26	3 (11.5)	17 (65.4)	27	1 (3.7)	13 (48.1)	6 (22.2)	1 (3.7)	6 (22.2)
Providencia rettgeri	938	130 (13.9)	653 (69.6)	1003	105 (10.5)	481 (48.0)	263 (26.2)	90 (9.0)	64 (6.4)
Providencia stuartii	1104	200 (18.1)	726 (65.8)	1165	195 (16.7)	440 (37.8)	326 (28.0)	163 (14.0)	41 (3.5)
Providencia spp	31	7 (22.6)	21 (67.7)	36	7 (19.4)	11 (30.6)	12 (33.3)	2 (5.6)	4 (11.1)
Raoultella ornithinolytica	89	19 (21.3)	63 (70.8)	98	41 (41.8)	16 (16.3)	21 (21.4)	8 (8.2)	12 (12.2)
Serratia marcescens	5811	1770 (30.5)	3364 (57.9)	6199	2473 (39.9)	684 (11.0)	1243 (20.1)	1234 (19.9)	425 (6.9)

[a] Does not include isolates from wards/infection sources for which information was not specified or available. [b] The number of isolates mentioned correspond to ICU, non-ICU and other sources (clinic/office, emergency room, nursing home/rehab). [c] The number of isolates mentioned correspond to RTI, UTI, SSTI, BSI, IAI, and other infection sources (nervous system, the head, ears, eyes, nose, and throat [HEENT], and instruments). [d] Includes *Enterobacter cloacae, Enterobacter hormaechi, Enterobacter kobei, Enterobacter ludwigii, Enterobacter asburiae, Escherichia coli, Klebsiella pneumoniae, Klebsiella oxytoca, Klebsiella aerogenes, Citrobacter koseri, Citrobacter freundii, Morganella morganii, Serratia marcescens, Proteus mirabilis, Citrobacter amalonaticus, Citrobacter braakii, Citrobacter farmer, Citrobacter* spp*, Enterobacter bugandensis, Enterobacter xiangfangensis, Klebsiella variicola, Proteus hauseri, Proteus vulgaris, Providencia alcalifaciens, Providencia rettgeri, Providencia* spp*, Providencia stuartii, Raoultella ornithinolytica*. % indicates n (from specific ward or infection source)/total n from wards or infection sources. BSI, blood stream infections; IAI, intra-abdominal infections; ICU, intensive care unit; N, total number of isolates; n, number of isolates from wards/infection sources; NA, not available; RTI, respiratory tract infections; SSTI, skin and soft tissue infection; UTI, urinary tract infections.

Table S2. Percentage frequency distribution at MIC (μg/mL) for all Enterobacterales collected globally across wards, 2016-2020.

			Cumulative percentage of isolates at each MIC (μg/mL) (absolute n at MIC)														
**Wards**	**Anti-micro-bial**	**n**	**≤0.015**	**0.03**	**0.06**	**0.12**	**0.25**	**0.5**	**1**	**2**	**4**	**8**	**16**	**32**	**64**	**128**	**≥256**
**Enteroba-cterales[a]**																	
**ICU**	ATM	20,799	5.4 (1114)	15.4 (2085)	37.3 (4563)	53.8 (3421)	59.7 (1232)	62.0 (472)	63.6 (333)	64.8 (259)	66.3 (304)	68.6 (480)	72.4 (798)	79.0 (1365)	85.9 (1440)	**94.6** (1813)	100 (1120)
	ATM-AVI	20,200	15.7 (3177)	43.6 (5625)	72.6 (5869)	86.6 (2824)	**94.0** (1497)	97.3 (656)	98.5 (249)	99.3 (155)	99.7 (94)	99.9 (24)	99.9 (17)	100 (3)	100 (6)	100 (4)	
**Non-ICU**	ATM	58,531	8.0 (4686)	19.2 (6530)	43.8 (14432)	61.5 (10349)	67.4 (3464)	69.6 (1257)	71.2 (929)	72.6 (830)	74.3 (995)	76.8 (1451)	80.2 (1989)	86.0 (3448)	**91.6** (3241)	96.7 (3017)	100 (1913)
	ATM-AVI	56,533	21.8 (12294)	51.3 (16730)	79.3 (15783)	**91.1** (6710)	95.9 (2672)	98.0 (1203)	99.0 (546)	99.5 (325)	99.9 (184)	99.9 (45)	100 (21)	100 (7)	100 (9)	100 (4)	

NOTE: MIC_{90} values are in bold, and the boxes have been shaded grey. [a] Includes Enterobacter cloacae, Enterobacter hormaechi, Enterobacter kobei, Enterobacter ludwigii, Enterobacter asburiae, Escherichia coli, Klebsiella pneumoniae, Klebsiella oxytoca, Klebsiella aerogenes, Citrobacter koseri, Citrobacter freundii, Morganella morganii, Serratia marcescens, Proteus mirabilis, Citrobacter amalonaticus, Citrobacter braakii, Citrobacter farmer, Citrobacter spp, Enterobacter bugandensis, Enterobacter xiangfangensis, Klebsiella variicola, Proteus hauseri, Proteus vulgaris, Providencia alcalifaciens, Providencia rettgeri, Providencia spp, Providencia stuartii, and Raoultella ornithinolytica. ATM, aztreonam; ATM-AVI, aztreonam/avibactam; ICU, intensive care unit; MIC, minimum inhibitory concentration; n, number of isolates from wards.

Table S3. *In vitro* activity of ATM-AVI and comparator agents tested against multi-drug resistant (MDR) Enterobacterales isolates across regions stratified by wards from 2016-2020.

	ICU				Non-ICU			
	CLSI		EUCAST		CLSI		EUCAST	
	n[a]	MIC$_{90}$ (μg/mL) (% S)	n[a]	MIC$_{90}$ (mg/L) (% S[b])	n[a]	MIC$_{90}$ (μg/mL) (% S)	n[a]	MIC$_{90}$ (mg/L) (% S[b])
**Africa-Middle East (CLSI/EUCAST, N=3627/3935)**								
Aztreonam-avibactam[c]	783	0.25 (100)	840	0.25 (100)	2255	0.25 (99.8)	2446	0.25 (99.8)
Aztreonam	783	128 (21.5)	840	128 (25.7)	2255	128 (26.7)	2446	128 (31.6)
Amikacin	805	16 (91.2)	869	16 (86.1)	2298	8 (94.6)	2501	8 (90.4)
Cefepime	805	64 (24.1)	869	64 (33.3)	2298	64 (27.7)	2501	64 (37.9)
Ceftazidime	805	128 (20.5)	869	128 (24.3)	2298	128 (27.6)	2501	128 (32.0)
Ceftazidime-avibactam	783	1 (92.5)	840	1 (93.0)	2255	1 (94.9)	2446	1 (95.3)
Ceftriaxone	184	64 (6.0)	203	64 (11.3)	587	32 (7.2)	651	32 (14.1)
Ciprofloxacin	621	8 (22.5)	666	8 (36.2)	1711	8 (19.7)	1850	8 (30.4)
Colistin[d,e]	726	1 (NA)	776	1 (96.3)	2015	1 (NA)	2186	1 (97.4)
Gentamicin	621	32 (49.3)	666	32 (51.1)	1711	32 (50.0)	1850	32 (51.1)
Imipenem[f]	783	8 (75.9)	840	8 (NA)	2255	4 (80.7)	2446	4 (NA)
Levofloxacin	805	16 (39.6)	869	16 (55.6)	2298	16 (30.8)	2501	16 (44.9)
Meropenem	805	16 (83.4)	869	16 (89.8)	2298	1 (91.0)	2501	0.5 (95.2)
Piperacillin/tazobactam	805	128 (46.8)	869	128 (48.7)	2298	128 (54.9)	2501	128 (56.4)
Tigecycline[g,h,i]	765	1 (96.9)	826	1 (96.7)	2071	1 (97.4)	2258	1 (96.3)
**Asia-Pacific (CLSI/EUCAST, N=8781/9441)**								
Aztreonam-avibactam[c]	1591	1 (98.5)	1668	1 (98.6)	4405	0.5 (99.6)	4723	0.5 (99.6)
Aztreonam	1975	256 (16.6)	2072	256 (19.9)	5440	256 (27.2)	5845	256 (31.1)
Amikacin	2029	128 (72.8)	2135	128 (70.5)	5605	64 (89)	6044	32 (86.2)
Cefepime	2029	64 (21.3)	2135	64 (29.2)	5605	64 (33.4)	6044	64 (42.5)
Ceftazidime	2029	256 (16)	2135	256 (18.7)	5605	256 (29.6)	6044	256 (33.3)
Ceftazidime-avibactam	1975	128 (81.0)	2072	128 (81.8)	5440	4 (91.6)	5845	2 (92.2)
Ceftriaxone	430	64 (5.4)	467	64 (12.4)	1726	32 (6.5)	1891	32 (11.4)
Ciprofloxacin	1599	8 (15.4)	1668	8 (22.9)	3879	8 (20.0)	4153	8 (29.1)
Colistin[d,e]	1787	1 (NA)	1876	1 (92.6)	4891	1 (NA)	5273	1 (94.7)
Gentamicin	1599	32 (45.2)	1668	32 (46)	3879	32 (55.1)	4153	32 (55.7)
Imipenem[f]	1975	16 (60.7)	2072	16 (0)	5440	16 (76.1)	5845	16 (NA)
Levofloxacin	2029	16 (22.4)	2135	16 (32.3)	5605	16 (26.0)	6044	16 (36.4)
Meropenem	2029	32 (67)	2135	32 (71.8)	5605	16 (84.4)	6044	16 (87.7)
Piperacillin/tazobactam	2029	128 (38.6)	2135	128 (40.0)	5605	128 (54.6)	6044	128 (56.3)
Tigecycline[g,h,i]	1880	2 (94.6)	1981	2 (94.2)	5130	2 (96.3)	5549	2 (93.5)
**Europe (CLSI/EUCAST, N=14785/16435)**								
Aztreonam-avibactam[c]	3807	0.5 (99.9)	4088	0.5 (99.9)	8026	0.5 (99.9)	8837	0.5 (99.9)
Aztreonam	3807	256 (22.4)	4088	256 (26.7)	8026	128 (29.3)	8837	128 (34.6)
Amikacin	4126	32 (87.4)	4473	32 (82.4)	8606	16 (91.5)	9583	16 (87.4)
Cefepime	4126	64 (31.0)	4473	64 (39.9)	8606	64 (37.4)	9583	64 (47.2)
Ceftazidime	4126	256 (23.8)	4473	256 (27.6)	8606	256 (30.1)	9583	256 (35)

Ceftazidime-avibactam	3807	2 (93.6)	4088	2 (94.1)	8026	2 (96.2)	8837	1 (96.5)
Ceftriaxone	1429	64 (9.5)	1595	64 (15.1)	3111	64 (10.9)	3586	64 (18.3)
Ciprofloxacin	2697	8 (29.6)	2878	8 (37.0)	5495	8 (28.1)	5997	8 (36.2)
Colistin[d,e]	3495	1 (NA)	3753	1 (92.3)	7197	1 (NA)	7937	1 (95.7)
Gentamicin	2697	32 (54.8)	2878	32 (55.9)	5495	32 (61.6)	5997	32 (62.4)
Imipenem[f]	3807	16 (72.0)	4088	16 (NA)	8026	8 (76.6)	8837	8 (NA)
Levofloxacin	4126	16 (34.1)	4473	16 (45.3)	8606	16 (33.6)	9583	16 (43.8)
Meropenem	4126	16 (77.3)	4473	16 (85.8)	8606	8 (85.1)	9583	4 (91.7)
Piperacillin/tazobactam	4126	256 (30.3)	4473	256 (32.8)	8606	256 (41.6)	9583	256 (43.8)
Tigecycline[g,h,i]	3872	2 (96.4)	4198	2 (96.8)	7850	1 (96.6)	8757	1 (96.7)
**Latin America (CLSI/EUCAST, N=6640/7083)**								
Aztreonam-avibactam[c]	1638	0.25 (100)	1715	0.25 (100)	3451	0.25 (99.9)	3640	0.25 (99.9)
Aztreonam	1638	256 (18.0)	1715	256 (20.4)	3451	128 (24.2)	3640	128 (27.4)
Amikacin	1736	32 (85.8)	1825	32 (79.1)	3675	16 (91.4)	3923	16 (85.9)
Cefepime	1736	64 (22.6)	1825	64 (30.3)	3675	64 (26.4)	3923	64 (34.2)
Ceftazidime	1736	256 (19.1)	1825	256 (21.3)	3675	128 (26.3)	3923	128 (29.2)
Ceftazidime-avibactam	1638	2 (92.9)	1715	2 (93.2)	3451	2 (95.4)	3640	1 (95.7)
Ceftriaxone	579	64 (5.5)	619	64 (8.9)	1387	64 (6.4)	1514	64 (9.8)
Ciprofloxacin	1157	8 (19.4)	1206	8 (27.3)	2288	8 (18.4)	2409	8 (25.5)
Colistin[d,e]	1504	1 (NA)	1582	1 (92.5)	3143	1 (NA)	3314	1 (94.8)
Gentamicin	1157	32 (45.8)	1206	32 (46.1)	2288	32 (50.2)	2409	32 (50.6)
Imipenem[f]	1638	16 (69.6)	1715	16 (NA)	3451	8 (77.6)	3640	8 (NA)
Levofloxacin	1736	16 (29.5)	1825	16 (41.9)	3675	16 (25.5)	3923	16 (35.7)
Meropenem	1736	32 (73.3)	1825	32 (81.8)	3675	16 (83.3)	3923	16 (89.9)
Piperacillin/tazobactam	1736	256 (37.4)	1825	256 (38.9)	3675	256 (48.7)	3923	128 (49.6)
Tigecycline[g,h,i]	1647	2 (96.7)	1739	2 (98.5)	3414	1 (97.4)	3649	1 (96.8)
**North America (CLSI/EUCAST, N=2472/2806)**								
Aztreonam-avibactam[c]	470	0.5 (99.8)	517	0.5 (99.8)	1427	0.5 (99.7)	1612	0.5 (99.7)
Aztreonam	470	128 (25.3)	517	128 (31.1)	1427	128 (38.5)	1612	128 (44.5)
Amikacin	506	8 (95.9)	555	8 (90.5)	1507	8 (97.8)	1720	8 (94.4)
Cefepime	506	64 (43.7)	555	64 (56.6)	1507	64 (54.7)	1720	64 (64.8)
Ceftazidime	506	128 (29.5)	555	128 (34.4)	1507	128 (38.3)	1720	128 (43.7)
Ceftazidime-avibactam	470	1 (98.9)	517	1 (99.0)	1428	1 (98.5)	1613	1 (98.8)
Ceftriaxone	108	64 (12.0)	121	64 (17.4)	243	64 (11.5)	308	64 (21.4)
Ciprofloxacin	398	8 (36.9)	434	8 (46.8)	1264	8 (36.4)	1412	8 (43.9)
Colistin[d,e]	435	1 (NA)	475	1 (96.6)	1259	1 (NA)	1432	1 (95.2)
Gentamicin	398	32 (71.9)	434	32 (71.2)	1264	32 (70.2)	1412	32 (70.4)
Imipenem[f]	470	2 (85.7)	517	2 (NA)	1428	4 (82.1)	1613	2 (NA)
Levofloxacin	506	16 (42.9)	555	16 (51.9)	1507	16 (40.8)	1720	16 (49.4)
Meropenem	506	1 (90.9)	555	0.5 (96.6)	1507	0.5 (92.6)	1720	0.25 (96.9)
Piperacillin/tazobactam	506	128 (41.5)	555	128 (44.9)	1507	128 (53.2)	1720	128 (55.4)
Tigecycline[g,h,i]	483	1 (95.5)	526	1 (97.6)	1356	1 (96.8)	1561	1 (97.2)

[a] Not all drugs in the panel were tested every year. [b] Data includes percentage isolates susceptible at increased exposure. [c] No breakpoints available from CLSI and EUCAST. Values expressed are indicative of the cumulative percentage of isolates inhibited at ≤8 mg/L for comparison purposes. [d] Susceptible category for colistin not available for CLSI breakpoints (only intermediate and resistant isolates are available). [e] Data for colistin do not include isolates of *Morganella morganii*, *Proteus hauseri*, *Proteus mirabilis*, *Proteus vulgaris*, *Providencia alcalifaciens*, *Providencia rettgeri*, *Providencia* spp,

Providencia stuartii, and *Serratia marcescens* because of their intrinsic resistance. [f] Data for imipenem not available per EUCAST. [g] Data for tigecycline do not include isolates of *Morganella morganii*, *Proteus hauseri*, *Proteus mirabilis*, *Proteus vulgaris*, *Providencia alcalifaciens*, *Providencia rettgeri*, *Providencia* spp, and *Providencia stuartii* due to their intrinsic resistance. [h] Data for tigecycline was calculated based on FDA approved breakpoints for CLSI. [i] EUCAST data for susceptibility to tigecycline are limited to *E. coli* and *C. koseri*; denominator (n): AfME: ICU= 239, non-ICU= 995; APAC: ICU= 694, non-ICU= 2885; Europe: ICU= 1104, non-ICU= 3343; LATAM: ICU= 469, non-ICU= 1627; North America: ICU= 207, non-ICU= 774. ICU, intensive care unit; MIC, minimum inhibitory concentration; N, total number of isolates; n, number of isolates from wards; NA, not available.

Table S4. *In vitro* activity of ATM-AVI and comparator agents tested against multi-drug resistant (MDR) Enterobacterales isolates across regions stratified by infection sources from 2016-2020.

	RTI				UTI				SSTI			
	CLSI		EUCAST		CLSI		EUCAST		CLSI		EUCAST	
	n[a]	MIC_{90} (µg/mL)/ (% S)	n[a]	MIC_{90} (mg/L) (% S[b])	n[a]	MIC_{90} (µg/mL) (% S)	n[a]	MIC_{90} (mg/L) (% S[b])	n[a]	MIC_{90} (µg/mL) (% S)	n[a]	MIC_{90} (mg/L) (% S[b])
**Africa-Middle East (CLSI/EUCAST, N=3627/3935)**												
Aztreonam-avibactam[c]	565	0.25 (100)	613	0.25 (100)	987	0.25 (99.8)	1047	0.25 (99.8)	835	0.25 (99.9)	919	0.25 (99.9)
Aztreonam	565	128 (21.2)	613	128 (26.3)	987	128 (27.9)	1047	128 (31.5)	835	128 (27.2)	919	128 (33.0)
Amikacin	576	16 (91.8)	626	16 (88.5)	1010	8 (95.5)	1072	8 (91.0)	852	16 (94.0)	946	16 (89.0)
Cefepime	576	64 (21.9)	626	64 (32.4)	1010	64 (27.7)	1072	64 (36.9)	852	64 (28.9)	946	64 (39.2)
Ceftazidime	576	128 (22.9)	626	128 (28.3)	1010	128 (28.8)	1072	128 (31.7)	852	128 (27.9)	946	128 (32.6)
Ceftazidime-avibactam	565	1 (95.6)	613	1 (95.9)	987	1 (93.4)	1047	1 (93.8)	835	1 (95.0)	919	1 (95.4)
Ceftriaxone	191	32 (7.9)	207	32 (11.6)	222	32 (5.4)	244	32 (12.7)	251	32 (5.6)	287	32 (14.3)
Ciprofloxacin	385	8 (19.5)	419	8 (32.0)	788	8 (19.0)	828	8 (27.2)	601	8 (19.5)	659	8 (32.3)
Colistin[d,e]	507	1 (NA)	549	1 (97.1)	887	1 (NA)	948	1 (97.2)	717	1 (NA)	786	1 (96.7)
Gentamicin	385	32 (51.2)	419	32 (51.6)	788	32 (48)	828	32 (48.9)	601	32 (47.8)	659	32 (49.2)
Imipenem[f]	565	4 (77.9)	613	4 (NA)	987	4 (82.3)	1047	4 (NA)	835	4 (79.0)	919	4 (NA)
Levofloxacin	576	16 (35.1)	626	16 (53.5)	1010	16 (30.8)	1072	16 (40.4)	852	16 (29.8)	946	16 (46.5)
Meropenem	576	8 (86.5)	626	4 (91.4)	1010	1 (91.2)	1072	0.5 (95.2)	852	1 (91.3)	946	0.5 (95.4)
Piperacillin/tazobactam	576	128 (48.8)	626	128 (49.7)	1010	128 (55.2)	1072	128 (56.7)	852	128 (57.2)	946	128 (58.9)
Tigecycline[g,h,i]	527	2 (95.5)	573	2 (94.9)	917	1 (98.4)	981	1 (97.2)	740	1 (97.3)	821	1 (94.7)
**Asia-Pacific (CLSI/EUCAST, N=8781/9441)**												
Aztreonam-avibactam[c]	1661	0.5 (99.2)	1763	0.5 (99.2)	1879	0.5 (99.4)	2014	0.5 (99.5)	1021	0.5 (99.3)	1100	0.5 (99.4)
Aztreonam	2052	256 (18.0)	2178	256 (21.8)	2299	256 (30.0)	2466	256 (33.4)	1203	256 (29.8)	1298	256 (34.0)
Amikacin	2127	128 (80.2)	2264	128 (78)	2354	128 (85.1)	2540	128 (82.1)	1239	64 (87.4)	1336	64 (84.4)
Cefepime	2127	64 (25.6)	2264	64 (34.5)	2354	64 (32.2)	2540	64 (41.8)	1239	64 (34.5)	1336	64 (43.6)
Ceftazidime	2127	256 (18.4)	2264	256 (21.3)	2354	256 (31.7)	2540	256 (35.2)	1239	256 (28.1)	1336	256 (32.0)
Ceftazidime-avibactam	2052	128 (88.4)	2178	128 (89)	2299	128 (88.6)	2466	128 (89.4)	1203	128 (89.4)	1298	8 (90.2)
Ceftriaxone	684	32 (5.4)	742	32 (9.8)	634	32 (6.0)	722	32 (13.6)	472	32 (8.7)	506	32 (14.4)
Ciprofloxacin	1443	8 (17.4)	1522	8 (26.6)	1720	8 (16.1)	1818	8 (23.2)	767	8 (21.9)	830	8 (32.5)
Colistin[d,e]	1870	1 (NA)	1987	1 (93.0)	2015	1 (NA)	2165	1 (96.1)	1015	1 (NA)	1102	1 (93.9)

Gentamicin	1443	32 (52.1)	1522	32 (52.5)	1720	32 (49.7)	1818	32 (50.2)	767	32 (51.0)	830	32 (51.8)
Imipenem[f]	2052	16 (67.2)	2178	16 (NA)	2299	16 (73.6)	2466	16 (NA)	1203	16 (69.6)	1298	16 (NA)
Levofloxacin	2127	16 (23.5)	2264	16 (36.0)	2354	16 (21.9)	2540	16 (30.2)	1239	16 (28.3)	1336	16 (39.6)
Meropenem	2127	32 (73.9)	2264	32 (78.8)	2354	32 (82.4)	2540	32 (86.0)	1239	32 (81.9)	1336	32 (86.2)
Piperacillin/tazobactam	2127	256 (37.9)	2264	256 (38.9)	2354	128 (56.5)	2540	128 (58.2)	1239	128 (54.5)	1336	128 (56.4)
Tigecycline[g,h,i]	2014	2 (94.0)	2146	2 (92.4)	2085	2 (96.9)	2256	1 (93.5)	1061	2 (95.2)	1150	2 (95.3)
**Europe (CLSI/EUCAST, N=14785/16435)**												
Aztreonam-avibactam[c]	3234	0.5 (99.8)	3496	0.5 (99.9)	3065	0.5 (99.8)	3360	0.25 (99.9)	2563	0.5 (99.9)	2780	0.5 (99.9)
Aztreonam	3234	256 (22.6)	3496	256 (27.3)	3065	128 (27.3)	3360	128 (32.4)	2563	128 (31.5)	2780	128 (35.9)
Amikacin	3473	32 (88.1)	3809	32 (83.4)	3297	16 (90.8)	3692	16 (86.9)	2731	16 (91.6)	2971	16 (87.7)
Cefepime	3473	64 (32.6)	3809	64 (41.5)	3297	64 (33.1)	3692	64 (43.0)	2731	64 (39.0)	2971	64 (48.8)
Ceftazidime	3473	256 (24.2)	3809	256 (28.4)	3297	256 (27.6)	3692	256 (32.4)	2731	256 (32.0)	2971	256 (36)
Ceftazidime-avibactam	3234	2 (94.3)	3496	2 (94.8)	3065	1 (95.5)	3360	1 (95.9)	2563	1 (96.5)	2780	1 (96.7)
Ceftriaxone	1388	64 (8.3)	1582	64 (15.4)	1333	64 (8.3)	1545	64 (14.8)	1085	64 (11.7)	1211	32 (20.1)
Ciprofloxacin	2085	8 (29.8)	2227	8 (36.9)	1964	8 (23.0)	2147	8 (31.1)	1646	8 (30.7)	1760	8 (37.1)
Colistin[d,e]	2986	1 (NA)	3224	1 (92.7)	2679	1 (NA)	2932	1 (95.2)	2207	1 (NA)	2400	1 (95.1)
Gentamicin	2085	32 (57.4)	2227	32 (58.2)	1964	32 (58)	2147	32 (58.9)	1646	32 (60.5)	1760	32 (60.5)
Imipenem[f]	3234	16 (73.7)	3496	8 (NA)	3065	8 (76.7)	3360	4 (NA)	2563	8 (73.2)	2780	8 (NA)
Levofloxacin	3473	16 (34.9)	3809	16 (45.9)	3297	16 (29.2)	3692	16 (40.2)	2731	16 (34.4)	2971	16 (44.6)
Meropenem	3473	16 (80.0)	3809	16 (88.1)	3297	8 (86.3)	3692	4 (92.3)	2731	8 (83.6)	2971	8 (91.8)
Piperacillin/tazobactam	3473	256 (30.4)	3809	256 (32.3)	3297	256 (44.0)	3692	128 (46.3)	2731	256 (40.8)	2971	256 (42.6)
Tigecycline[g,h,i]	3282	2 (96.4)	3597	2 (96.5)	2928	1 (96.7)	3281	1 (97.4)	2400	1 (96.5)	2614	1 (96.6)
**Latin America (CLSI/EUCAST, N=6640/7083)**												
Aztreonam-avibactam[c]	950	0.25 (100)	986	0.25 (100)	1632	0.25 (100)	1758	0.25 (100)	1119	0.5 (99.9)	1171	0.25 (99.9)
Aztreonam	950	256 (17.3)	986	256 (18.7)	1632	128 (29.0)	1758	128 (33.2)	1119	256 (25.2)	1171	256 (28.0)
Amikacin	1005	32 (89.3)	1047	32 (83.5)	1706	16 (90.9)	1844	16 (85.5)	1188	16 (90.9)	1258	16 (84.7)
Cefepime	1005	64 (22.7)	1047	64 (29)	1706	64 (28.7)	1844	64 (37.9)	1188	64 (27.5)	1258	64 (35.5)
Ceftazidime	1005	128 (17.8)	1047	128 (19.3)	1706	128 (31.4)	1844	128 (35.4)	1188	256 (25.8)	1258	128 (28.4)
Ceftazidime-avibactam	950	2 (95.5)	986	2 (95.6)	1632	1 (95.3)	1758	1 (95.7)	1119	2 (92.4)	1171	2 (92.7)
Ceftriaxone	334	64 (4.8)	350	64 (7.4)	575	64 (6.3)	633	64 (11.9)	481	32 (7.9)	534	64 (14.4)
Ciprofloxacin	671	8 (17.1)	697	8 (23.1)	1131	8 (17.2)	1211	8 (24.0)	707	8 (18.3)	724	8 (25.6)
Colistin[d,e]	870	2 (NA)	903	1 (91.9)	1467	1 (NA)	1577	1 (95.6)	974	1 (NA)	1025	1 (94.2)
Gentamicin	671	32 (45.3)	697	32 (45.9)	1131	32 (48.8)	1211	32 (49.1)	707	32 (44.4)	724	32 (44.2)
Imipenem[f]	950	16 (73.1)	986	16 (NA)	1632	8 (78.1)	1758	8 (NA)	1119	16 (73.1)	1171	16 (NA)
Levofloxacin	1005	16 (26.8)	1047	16 (38.2)	1706	16 (22.6)	1844	16 (32.2)	1188	16 (26.4)	1258	16 (37.4)
Meropenem	1005	32 (77.7)	1047	32 (84.1)	1706	16 (84.2)	1844	8 (90.2)	1188	16 (82.4)	1258	16 (89.2)
Piperacillin/tazobactam	1005	256 (39.9)	1047	256 (40.1)	1706	256 (49.9)	1844	128 (51.7)	1188	256 (49.0)	1258	128 (49.9)
Tigecycline[g,h,i]	955	2 (96.2)	999	2 (98.6)	1557	1 (98.1)	1683	1 (98.1)	1060	1 (97.6)	1129	1 (96.4)

**North America (CLSI/EUCAST, N=2472/2806)**												
Aztreonam-avibactam[c]	558	0.5 (99.8)	611	0.5 (99.7)	564	0.5 (99.8)	631	0.5 (99.8)	350	1 (99.1)	394	1 (99.2)
Aztreonam	558	128 (26.9)	611	128 (32.4)	564	128 (39.2)	631	128 (44.1)	350	128 (41.1)	394	128 (46.5)
Amikacin	591	16 (95.1)	647	16 (89.2)	588	8 (97.3)	667	8 (94.6)	371	8 (99.5)	416	8 (94.5)
Cefepime	591	64 (49.1)	647	64 (59.5)	588	64 (53.6)	667	64 (63.6)	371	64 (53.6)	416	64 (67.8)
Ceftazidime	591	128 (29.4)	647	128 (34.5)	588	128 (39.3)	667	128 (43.3)	371	128 (41)	416	128 (46.4)
Ceftazidime-avibactam	558	1 (98.6)	611	1 (98.7)	565	1 (98.4)	632	1 (98.7)	350	1 (97.4)	394	1 (97.7)
Ceftriaxone	112	64 (9.8)	129	64 (18.6)	89	64 (9.0)	113	64 (15.0)	74	32 (16.2)	88	32 (30.7)
Ciprofloxacin	479	8 (38.0)	518	8 (45.8)	499	8 (36.9)	554	8 (44.4)	297	8 (39.4)	328	8 (47.9)
Colistin[d,e]	501	1 (NA)	549	1 (95.6)	482	1 (NA)	551	1 (95.6)	282	1 (NA)	321	1 (94.1)
Gentamicin	479	32 (67.0)	518	32 (65.8)	499	32 (68.9)	554	32 (69.5)	297	32 (71.7)	328	32 (71.3)
Imipenem[f]	558	4 (81.9)	611	4 (NA)	565	4 (81.2)	632	2 (NA)	350	4 (72.6)	394	4 (NA)
Levofloxacin	591	16 (41.5)	647	16 (51.6)	588	16 (41.7)	667	16 (50.8)	371	16 (45.0)	416	16 (54.6)
Meropenem	591	2 (89.9)	647	1 (95.2)	588	0.5 (93.9)	667	0.25 (97.5)	371	0.5 (90.8)	416	0.5 (97.1)
Piperacillin/tazobactam	591	128 (41.6)	647	128 (44.4)	588	128 (57.1)	667	128 (58.9)	371	128 (56.1)	416	128 (57.5)
Tigecycline[g,h,i]	559	1 (95.9)	612	1 (98.1)	508	1 (96.1)	589	1 (95.2)	305	2 (94.1)	346	1 (96.1)

[a] Not all drugs in the panel were tested every year. [b] Data includes percentage isolates susceptible at increased exposure. [c] No breakpoints available from CLSI and EUCAST. Values expressed are indicative of the cumulative percentage of isolates inhibited at ≤8 mg/L for comparison purposes. [d] Susceptible category for colistin not available for CLSI breakpoints (only intermediate and resistant isolates are available). [e] Data for colistin do not include isolates of *Morganella morganii*, *Proteus hauseri*, *Proteus mirabilis*, *Proteus vulgaris*, *Providencia alcalifaciens*, *Providencia rettgeri*, *Providencia* spp, *Providencia stuartii*, and *Serratia marcescens* because of their intrinsic resistance. [f] Data for imipenem not available per EUCAST. [g] Data for tigecycline do not include isolates of *Morganella morganii*, *Proteus hauseri*, *Proteus mirabilis*, *Proteus vulgaris*, *Providencia alcalifaciens*, *Providencia rettgeri*, *Providencia* spp, and *Providencia stuartii* due to their intrinsic resistance. [h] Data for tigecycline was calculated based on FDA approved breakpoints for CLSI. [i] EUCAST data for susceptibility to tigecycline are limited to *E. coli* and *C. Koseri*; denominator (n): AfME: RTI= 136, UTI= 498, SSTI= 357; APAC: RTI= 528, UTI= 1361, SSTI= 548; Europe: RTI= 855, UTI= 1171, SSTI= 935; LATAM: RTI= 208, UTI= 790, SSTI= 504; North America: RTI= 213, UTI= 293, SSTI= 154. MIC, minimum inhibitory concentration; N, total number of isolates; n, number of isolates from infection sources; NA, not available; RTI, respiratory tract infection; SSTI, skin and soft tissue infection; UTI, urinary tract infection.

Table S4. *Cont.*

	BSI				IAI			
	CLSI		EUCAST		CLSI		EUCAST	
	**n[a]**	**MIC_{90} (µg/mL) (% S)**	**n[a]**	**MIC_{90} (mg/L) (% S[b])**	**n[a]**	**MIC_{90} (µg/mL) (% S)**	**n[a]**	**MIC_{90} (mg/L) (% S[b])**
**Africa-Middle East (CLSI/EUCAST, N=3627/3935)**								
Aztreonam-avibactam[c]	750	0.25 (99.7)	802	0.25 (99.8)	399	0.25 (99.8)	436	0.25 (99.8)
Aztreonam	750	128 (20.7)	802	128 (25.4)	399	128 (30.6)	436	128 (35.1)
Amikacin	762	16 (92.8)	820	16 (88.4)	413	8 (95.4)	454	8 (91.2)
Cefepime	762	64 (23.4)	820	64 (32.3)	413	64 (34.6)	454	64 (44.5)

Ceftazidime	762	128 (19.3)	820	128 (23.7)	413	128 (31.2)	454	128 (35.2)
Ceftazidime-avibactam	750	1 (92.0)	802	1 (92.5)	399	1 (96.0)	436	1 (96.3)
Ceftriaxone	113	32 (8.0)	124	64 (13.7)	101	32 (12.9)	112	64 (18.8)
Ciprofloxacin	649	8 (22.0)	696	8 (33.5)	312	8 (23.7)	342	8 (37.7)
Colistin[d,e]	709	1 (NA)	751	1 (97.1)	365	1 (NA)	400	1 (97.5)
Gentamicin	649	32 (49.3)	696	32 (50.7)	312	32 (56.4)	342	32 (56.4)
Imipenem[f]	750	8 (79.7)	802	8 (NA)	399	4 (79.7)	436	4 (NA)
Levofloxacin	762	16 (37.1)	820	16 (51.5)	413	16 (32.9)	454	16 (49.3)
Meropenem	762	8 (86.8)	820	8 (91.1)	413	2 (88.6)	454	2 (93.4)
Piperacillin/tazobactam	762	128 (50.9)	820	128 (52.7)	413	128 (53.8)	454	128 (55.1)
Tigecycline[g,h,i]	729	1 (97.8)	780	1 (97.3)	383	1 (96.1)	423	1 (96.7)
**Asia-Pacific (CLSI/EUCAST, N=8781/9441)**								
Aztreonam-avibactam[c]	1338	0.5 (99.5)	1421	0.5 (99.5)	967	0.5 (99.6)	1037	0.5 (99.6)
Aztreonam	1698	256 (22.1)	1811	256 (26.4)	1230	128 (25.8)	1322	128 (29.9)
Amikacin	1766	128 (83.4)	1908	128 (81.0)	1277	16 (90.5)	1375	16 (87.4)
Cefepime	1766	64 (24.9)	1908	64 (33.9)	1277	64 (36.4)	1375	64 (45.5)
Ceftazidime	1766	256 (26.9)	1908	256 (30.4)	1277	256 (29.4)	1375	256 (33.5)
Ceftazidime-avibactam	1698	128 (87.2)	1811	128 (88.0)	1230	2 (92.6)	1322	2 (93.1)
Ceftriaxone	289	64 (5.2)	343	64 (11.4)	442	32 (6.3)	475	32 (12.4)
Ciprofloxacin	1477	8 (16.6)	1565	8 (24.7)	835	8 (25.3)	900	8 (34.1)
Colistin[d,e]	1574	1 (NA)	1679	1 (94.5)	1142	1 (NA)	1239	1 (93.6)
Gentamicin	1477	32 (50.6)	1565	32 (51.4)	835	32 (59.5)	900	32 (60.6)
Imipenem[f]	1698	16 (73.0)	1811	16 (NA)	1230	16 (78.9)	1322	8 (NA)
Levofloxacin	1766	16 (22.4)	1908	16 (32.2)	1277	16 (31.6)	1375	16 (41.2)
Meropenem	1766	32 (78.8)	1908	32 (81.9)	1277	16 (85.8)	1375	16 (88.9)
Piperacillin/tazobactam	1766	128 (54.0)	1908	128 (56.0)	1277	128 (56.0)	1375	128 (57.8)
Tigecycline[g,h,i]	1668	2 (96.8)	1805	2 (94.4)	1199	1 (96.9)	1302	1 (94.1)
**Europe (CLSI/EUCAST, N=14785/16435)**								
Aztreonam-avibactam[c]	2842	0.5 (99.9)	3137	0.25 (99.9)	1864	0.5 (100)	2081	0.5 (100)
Aztreonam	2842	128 (30.0)	3137	128 (35.9)	1864	128 (27.1)	2081	128 (32.9)
Amikacin	3140	16 (90.9)	3540	16 (86.0)	2056	16 (91.9)	2308	16 (87.4)
Cefepime	3140	64 (34.8)	3540	64 (44.2)	2056	64 (39.4)	2308	64 (50.3)
Ceftazidime	3140	256 (30.2)	3540	128 (35.5)	2056	256 (30.4)	2308	256 (36)
Ceftazidime-avibactam	2842	2 (95.9)	3137	2 (96.2)	1864	2 (95.7)	2081	2 (96.2)
Ceftriaxone	689	64 (11.9)	836	64 (16.9)	783	64 (13.5)	904	64 (21.5)
Ciprofloxacin	2451	8 (26.9)	2704	8 (35.8)	1273	8 (33.6)	1404	8 (41.7)
Colistin[d,e]	2624	1 (NA)	2902	1 (94.9)	1751	1 (NA)	1968	1 (95.6)
Gentamicin	2451	32 (56.5)	2704	32 (58.5)	1273	32 (67.1)	1404	32 (67.9)
Imipenem[f]	2842	8 (76.2)	3137	8 (NA)	1864	8 (78.3)	2081	8 (NA)
Levofloxacin	3140	16 (30.7)	3540	16 (41.2)	2056	16 (38.8)	2308	16 (47.8)
Meropenem	3140	16 (81.1)	3540	16 (88.3)	2056	16 (83)	2308	16 (90)
Piperacillin/tazobactam	3140	128 (39.6)	3540	128 (42.7)	2056	256 (37.5)	2308	256 (40.7)
Tigecycline[g,h,i]	2956	2 (96.2)	3342	2 (97.0)	1952	1 (96.1)	2202	1 (96.2)

**Latin America (CLSI/EUCAST, N=6640/7083)**								
Aztreonam-avibactam[c]	1478	0.25 (99.9)	1562	0.25 (99.9)	1074	0.25 (100)	1128	0.25 (100)
Aztreonam	1478	256 (19.8)	1562	256 (23.3)	1074	256 (22.3)	1128	256 (25.5)
Amikacin	1548	32 (87.7)	1656	32 (81.6)	1170	16 (92.8)	1251	16 (86.9)
Cefepime	1548	64 (23.1)	1656	64 (31.4)	1170	64 (26.8)	1251	64 (34.5)
Ceftazidime	1548	128 (22.2)	1656	128 (25.5)	1170	128 (27.0)	1251	128 (29.7)
Ceftazidime-avibactam	1478	2 (93.9)	1562	2 (94.2)	1074	2 (96.1)	1128	2 (96.3)
Ceftriaxone	394	64 (5.1)	434	64 (7.4)	505	64 (7.3)	554	64 (10.3)
Ciprofloxacin	1154	8 (19.0)	1222	8 (27.7)	665	8 (23.0)	697	8 (30.6)
Colistin[d,e]	1359	1 (NA)	1443	1 (93.0)	1020	1 (NA)	1073	1 (96.2)
Gentamicin	1154	32 (51.0)	1222	32 (51.8)	665	32 (55.9)	697	32 (56.0)
Imipenem[f]	1478	16 (72.7)	1562	16 (NA)	1074	8 (81.1)	1128	8 (NA)
Levofloxacin	1548	16 (30.0)	1656	16 (42.8)	1170	16 (26.8)	1251	16 (35.9)
Meropenem	1548	16 (76.6)	1656	16 (85.0)	1170	16 (83.8)	1251	8 (90.3)
Piperacillin/tazobactam	1548	128 (44.2)	1656	128 (45.8)	1170	256 (47.3)	1251	256 (48.4)
Tigecycline[g,h,i]	1477	1 (97.6)	1585	1 (97.5)	1121	1 (97.4)	1201	1 (96.5)
**North America (CLSI/EUCAST, N=2472/2806)**								
Aztreonam-avibactam[c]	596	0.5 (99.8)	678	0.25 (99.9)	270	0.5 (100)	312	0.5 (100)
Aztreonam	596	128 (38.9)	678	128 (45.9)	270	128 (37.8)	312	128 (44.9)
Amikacin	620	8 (97.3)	727	8 (94.1)	296	8 (97.3)	340	8 (95.6)
Cefepime	620	64 (50.8)	727	64 (59.7)	296	64 (56.4)	340	32 (68.2)
Ceftazidime	620	128 (39.4)	727	128 (44.7)	296	128 (38.2)	340	128 (45.6)
Ceftazidime-avibactam	596	1 (99.8)	678	1 (99.9)	270	1 (98.9)	312	1 (99.0)
Ceftriaxone	44	64 (13.6)	75	64 (20.0)	76	64 (13.2)	86	64 (17.4)
Ciprofloxacin	576	8 (29.2)	652	8 (39.4)	220	8 (46.4)	254	8 (55.1)
Colistin[d,e]	555	1 (NA)	628	1 (97.0)	258	0.5 (NA)	296	0.5 (96.6)
Gentamicin	576	32 (68.4)	652	32 (69.8)	220	32 (76.4)	254	32 (76.8)
Imipenem[f]	596	2 (89.1)	678	2 (NA)	270	2 (85.6)	312	2 (NA)
Levofloxacin	620	16 (36.1)	727	16 (45.8)	296	16 (46.3)	340	16 (52.1)
Meropenem	620	0.25 (96.1)	727	0.12 (98.4)	296	2 (88.5)	340	0.5 (95.6)
Piperacillin/tazobactam	620	128 (60.0)	727	128 (62.3)	296	128 (45.6)	340	128 (50.3)
Tigecycline[g,h,i]	584	1 (98.3)	684	1 (98.5)	285	1 (97.5)	325	1 (96.7)

[a] Not all drugs in the panel were tested every year. [b] Data includes percentage isolates susceptible at increased exposure. [c] No breakpoints available from CLSI and EUCAST. Values expressed are indicative of the cumulative percentage of isolates inhibited at ≤8 mg/L for comparison purposes. [d] Susceptible category for colistin not available for CLSI breakpoints (only intermediate and resistant isolates are available). [e] Data for colistin do not include isolates of *Morganella morganii*, *Proteus hauseri*, *Proteus mirabilis*, *Proteus vulgaris*, *Providencia alcalifaciens*, *Providencia rettgeri*, *Providencia* spp, *Providencia stuartii*, and *Serratia marcescens* because of their intrinsic resistance. [f] Data for imipenem not available per EUCAST. [g] Data for tigecycline do not include isolates of *Morganella morganii*, *Proteus hauseri*, *Proteus mirabilis*, *Proteus vulgaris*, *Providencia alcalifaciens*, *Providencia rettgeri*, *Providencia* spp, and *Providencia stuartii* due to their intrinsic resistance. [h] Data for tigecycline was calculated based on FDA approved breakpoints for CLSI. [i] EUCAST data for susceptibility to tigecycline are limited to *E. coli* and *C. Koseri*; denominator (n): AfME: BSI= 293, IAI= 211; APAC: BSI= 1027, IAI=731; Europe: BSI= 1377, IAI= 986; LATAM: BSI= 570, IAI=624; North America: BSI= 398, IAI=179. BSI,

bloodstream infections; IAI, intra-abdominal infection; MIC, minimum inhibitory concentration; N, total number of isolates; n, number of isolates from infection sources; NA, not available.

Table S5. *In vitro* activity of ATM-AVI and comparator agents tested against ESBL-producing Enterobacterales isolates across regions stratified by wards from 2016-2020.

	ICU		Non-ICU	
**Africa-Middle East (N=2176)**	**n[b]**	**MIC_{90} (µg/mL) (% S, CLSI/%S, EUCAST[a])**	**n[b]**	**MIC_{90} (µg/mL) (% S, CLSI/%S, EUCAST[a])**
Aztreonam-avibactam[c]	476	0.25 (100)	1273	0.25 (99.9)
Aztreonam	476	128 (6.9/6.9)	1273	128 (8.2/8.2)
Amikacin	503	32 (89.9/83.7)	1369	16 (94.0/88.9)
Cefepime	503	64 (7.0/11.5)	1369	64 (7.2/13.7)
Ceftazidime	503	128 (9.9/9.9)	1369	128 (13.1/13.1)
Ceftazidime-avibactam	476	2 (90.6/90.6)	1273	1 (92.9/92.9)
Ceftriaxone	168	64 (1.2/2.4)	565	64 (3.0/3.2)
Ciprofloxacin	335	8 (16.4/26.9)	804	8 (12.1/21.1)
Colistin[d,e]	463	1 (NA/98.1)	1236	1 (NA/98.7)
Gentamicin	335	32 (39.1/38.8)	804	32 (46.1/44.7)
Imipenem[f]	476	16 (74.8/NA)	1273	4 (84.1/NA)
Levofloxacin	503	16 (36.2/50.3)	1369	16 (28.6/41.1)
Meropenem	503	16 (79.5/85.5)	1369	2 (89.0/93.4)
Piperacillin/tazobactam	503	128 (46.7/46.7)	1369	128 (54.1/54.1)
Tigecycline[g,h,i]	493	1 (96.6/98.5)	1332	1 (97.4/97.2)
**Asia-Pacific (N=4106)**				
Aztreonam-avibactam[c]	845	1 (98.6)	2020	0.5 (99.9)
Aztreonam	998	256 (5.1/5.1)	2306	256 (10.9/10.9)
Amikacin	1025	128 (64.7/61.0)	2472	64 (87.7/83.3)
Cefepime	1025	64 (8.3/11.4)	2472	64 (13.0/19.9)
Ceftazidime	1025	256 (8.6/8.6)	2472	256 (18.9/18.9)
Ceftazidime-avibactam	998	256 (71.6/71.6)	2306	128 (88.0/88.0)
Ceftriaxone	304	32 (4.0/5.6)	1317	64 (4.2/5.9)
Ciprofloxacin	721	8 (6.4/9.9)	1155	8 (9.8/13.8)
Colistin[d,e]	958	1 (NA/94.2)	2234	1 (NA/96.1)
Gentamicin	721	32 (36.5/35.5)	1155	32 (49.4/48.1)
Imipenem[f]	998	16 (52.6/NA)	2306	16 (77.8/NA)
Levofloxacin	1025	16 (14.8/21.9)	2472	16 (19.7/27.4)
Meropenem	1025	32 (55.9/60.3)	2472	32 (81.5/84.2)
Piperacillin/tazobactam	1025	128 (32.6/32.6)	2472	128 (54.7/54.7)
Tigecycline[g,h,i]	992	2 (95.6/96.3)	2407	2 (97.0/95.9)
**Europe (N=8807)**				
Aztreonam-avibactam[c]	2082	0.25 (99.9)	4226	0.25 (99.9)
Aztreonam	2082	256 (7.3/7.3)	4226	256 (10.0/10.0)
Amikacin	2453	64 (86.3/80.3)	5014	16 (91.0/85.7)
Cefepime	2453	64 (9.6/13.8)	5014	64 (13.1/18.7)
Ceftazidime	2453	256 (12.8/12.8)	5014	256 (17.2/17.2)
Ceftazidime-avibactam	2082	4 (91.9/91.9)	4226	2 (94.8/94.8)
Ceftriaxone	1256	64 (3.3/4.6)	2879	64 (4.3/6.0)
Ciprofloxacin	1197	8 (9.8/15.1)	2135	8 (12.0/17.1)
Colistin[d,e]	2020	2 (NA/91.5)	4055	1 (NA/95.2)

Gentamicin	1197	32 (41.9/41.3)	2135	32 (50.1/49.5)
Imipenem[f]	2082	16 (70.0/NA)	4226	8 (78.4/NA)
Levofloxacin	2453	16 (21.9/32.4)	5014	16 (24.3/31.9)
Meropenem	2453	16 (74.9/83.3)	5014	8 (83.5/90.0)
Piperacillin/tazobactam	2453	256 (36.0/36.0)	5014	256 (47.5/47.5)
Tigecycline[g,h,i]	2397	2 (96.5/98.2)	4855	1 (96.7/98.4)
**Latin America (N=4115)**				
Aztreonam-avibactam[c]	995	0.25 (100)	2076	0.25 (100)
Aztreonam	995	256 (6.2/6.2)	2076	256 (7.4/7.4)
Amikacin	1077	32 (84.9/77.8)	2346	16 (92.2/86.5)
Cefepime	1077	64 (7.8/13.1)	2346	64 (7.8/13.0)
Ceftazidime	1077	256 (11.7/11.7)	2346	256 (15.4/15.4)
Ceftazidime-avibactam	995	4 (91.1/91.1)	2076	2 (94.7/94.7)
Ceftriaxone	481	64 (4.0/4.2)	1267	64 (1.7/3.0)
Ciprofloxacin	596	8 (8.6/13.8)	1079	8 (8.3/13.0)
Colistin[d,e]	970	1 (NA/93.0)	2016	1 (NA/96.1)
Gentamicin	596	32 (34.6/33.2)	1079	32 (38.7/38.2)
Imipenem[f]	995	16 (71.7/NA)	2076	8 (83.0/NA)
Levofloxacin	1077	16 (21.7/34.1)	2346	16 (18.8/28.3)
Meropenem	1077	16 (73.4/81.1)	2346	8 (84.9/90.8)
Piperacillin/tazobactam	1077	256 (40.9/40.9)	2346	256 (51.2/51.2)
Tigecycline[g,h,i]	1058	1 (97.3/99.0)	2298	1 (97.6/97.7)
**North America (N=1099)**				
Aztreonam-avibactam[c]	218	0.25 (100)	535	0.25 (99.8)
Aztreonam	218	128 (9.2/9.2)	535	128 (13.3/13.3)
Amikacin	254	16 (95.3/89.4)	636	8 (95.8/90.7)
Cefepime	254	64 (20.5/29.5)	636	64 (19.0/26.9)
Ceftazidime	254	128 (16.5/16.5)	636	128 (19.5/19.5)
Ceftazidime-avibactam	218	1 (98.6/98.6)	535	1 (97.6/97.6)
Ceftriaxone	113	64 (7.1/9.7)	280	64 (8.6/10.4)
Ciprofloxacin	141	8 (14.9/24.1)	356	8 (10.7/15.7)
Colistin[d,e]	211	0.5 (NA/96.2)	513	0.5 (NA/98.1)
Gentamicin	141	32 (62.4/59.6)	356	32 (57.0/56.7)
Imipenem[f]	218	2 (87.2/NA)	535	2 (87.7/NA)
Levofloxacin	254	16 (28.4/37.8)	636	16 (25.2/32.9)
Meropenem	254	0.5 (91.3/95.7)	636	0.5 (91.8/95.9)
Piperacillin/tazobactam	254	128 (53.5/53.5)	636	128 (59.4/59.4)
Tigecycline[g,h,i]	247	1 (96.4/97.4)	615	1 (98.4/96.4)

[a] Data includes percentage isolates susceptible at increased exposure. [b] Not all drugs in the panel were tested every year. [c] No breakpoints available from CLSI and EUCAST. Values expressed are indicative of the cumulative percentage of isolates inhibited at ≤8 mg/L for comparison purposes. [d] Susceptible category for colistin not available for CLSI breakpoints (only intermediate and resistant isolates are available). [e] Data for colistin do not include isolates of *Morganella morganii*, *Proteus hauseri*, *Proteus mirabilis*, *Proteus vulgaris*, *Providencia alcalifaciens*, *Providencia rettgeri*, *Providencia* spp, *Providencia stuartii*, and *Serratia marcescens* because of their intrinsic resistance. [f] Data for imipenem not available per EUCAST. [g] Data for tigecycline do not include isolates of *Morganella morganii*, *Proteus hauseri*, *Proteus mirabilis*, *Proteus vulgaris*, *Providencia alcalifaciens*, *Providencia rettgeri*, *Providencia* spp, and *Providencia stuartii* due to their intrinsic resistance. [h] Data for tigecycline was calculated based on FDA approved breakpoints for CLSI. [i] EUCAST data for susceptibility to tigecycline are limited to *E. coli* and *C. koseri;* denominator (n): AfME: ICU=129, non-ICU=536; APAC: ICU=327, non-ICU=1261; Europe: ICU=608, non-ICU=1724; LATAM: ICU=286, non-ICU=1071; North America:

ICU=113, non-ICU=331. ICU, intensive care unit; MIC, minimum inhibitory concentration; N, total number of isolates; n, number of isolates from wards; NA, not available.

Table S6. *In vitro* activity of ATM-AVI and comparator agents tested against ESBL-producing Enterobacterales isolates across regions stratified by infection sources from 2016-2020.

	MIC90 (µg/mL) (% S, CLSI/%S, EUCAST[a])									
	RTI		UTI		SSTI		BSI		IAI	
**Africa-Middle East (N=2176)**	**n[b]**		**n[b]**		**n[b]**		**n[b]**		**n[b]**	
Aztreonam-avibactam[c]	373	0.25 (100)	523	0.25 (100)	469	0.25 (100)	438	0.25 (100)	215	0.25 (99.5)
Aztreonam	373	128 (8.9/8.9)	523	128 (4.8/4.8)	469	128 (9.8/9.8)	438	128 (4.6/4.6)	215	128 (9.3/9.3)
Amikacin	390	16 (91.5/87.4)	565	16 (94.7/88.9)	510	16 (93.9/88.4)	467	16 (91.7/86.9)	237	16 (93.7/89)
Cefepime	390	64 (5.9/12.1)	565	64 (5/11.7)	510	64 (10.0/16.9)	467	64 (5.6/10.3)	237	64 (10.6/16.5)
Ceftazidime	390	128 (14.9/14.9)	565	256 (9.6/9.6)	510	128 (15.5/15.5)	467	256 (8.6/8.6)	237	128 (13.1/13.1)
Ceftazidime-avibactam	373	1 (94.1/94.1)	523	2 (91.4/91.4)	469	1 (93.4/93.4)	438	128 (88.8/88.8)	215	1 (94/94)
Ceftriaxone	177	32 (4.0/4.0)	204	64 (2.0/2.0)	249	64 (3.6/4.4)	118	64 (0.9/1.7)	91	64 (1.1/2.2)
Ciprofloxacin	213	8 (13.2/21.6)	361	8 (12.2/20.2)	261	8 (11.9/25.7)	349	8 (16.3/23.8)	146	8 (13.0/25.3)
Colistin[d,e]	356	1 (NA/98.9)	510	1 (NA/98.6)	457	1 (NA/97.6)	430	1 (NA/98.8)	209	1 (0/99.5)
Gentamicin	213	32 (44.6/42.7)	361	32 (42.1/41.6)	261	32 (47.5/46.4)	349	32 (43.6/42.1)	146	32 (50/48.6)
Imipenem[f]	373	4 (79.9/NA)	523	4 (84.9/90.8)	469	4 (84.9/NA)	438	8 (79.9/85.4)	215	8 (79.1/NA)
Levofloxacin	390	16 (31.5/49.2)	565	16 (28.9/35.6)	510	16 (28.2/42.6)	467	16 (36.2/49.9)	237	16 (28.3/44.3)
Meropenem	390	16 (84.9/89.2)	565	2 (88.9/93.5)	510	2 (89.8/93.7)	467	16 (83.3/86.9)	237	8 (84.8/90.3)
Piperacillin/tazobactam	390	128 (50.0/50.0)	565	128 (52.4/52.4)	510	128 (57.5/57.5)	467	128 (49.3/49.3)	237	128 (54.9/54.9)
Tigecycline[g,h,i]	374	2 (95.5/96.5)	553	1 (98.2/98.0)	499	1 (97.4/96.8)	461	1 (98.1/98.7)	231	1 (95.7/98.0)
**Asia-Pacific (N=4106)**										
Aztreonam-avibactam[c]	872	0.5 (99.7)	875	0.5 (99.1)	528	0.5 (99.4)	601	0.25 (99.7)	424	0.5 (100)
Aztreonam	964	256 (7.3/7.3)	1052	256 (12.0/12.0)	596	256 (11.6/11.6)	728	256 (6.3/6.3)	485	256 (13.4/13.4)
Amikacin	1023	128 (77.8/74.2)	1105	128 (81.4/76.6)	619	128 (84.2/79.3)	819	128 (79.4/75.6)	526	64 (88.4/84.2)
Cefepime	1023	64 (11.1/16.5)	1105	64 (12.2/20.1)	619	64 (12.4/18.3)	819	64 (8.4/14.0)	526	64 (15.4/21.1)
Ceftazidime	1023	256 (10.6/10.6)	1105	256 (20.0/20.0)	619	256 (15.8/15.8)	819	256 (17.2/17.2)	526	256 (23.2/23.2)
Ceftazidime-avibactam	964	128 (82.7/82.7)	1052	128 (84.8/84.8)	596	128 (83.9/83.9)	728	256 (79.3/79.3)	485	128 (88.5/88.5)
Ceftriaxone	535	32 (4.3/5.8)	468	64 (4.3/6.4)	365	32 (4.9/7.7)	270	64 (2.6/4.4)	294	64 (4.8/5.1)
Ciprofloxacin	488	8 (6.6/11.1)	637	8 (8.0/11.2)	254	8 (10.6/14.6)	549	8 (9.8/12.9)	232	8 (12.5/15.5)
Colistin[d,e]	939	1 (NA/94.6)	1005	1 (NA/96.5)	573	1 (NA/96.2)	705	1 (NA/95.2)	474	1 (NA/95.6)
Gentamicin	488	32 (39.6/38.3)	637	32 (45.4/44.4)	254	32 (41.3/40.2)	549	32 (45.9/44.3)	232	32 (51.3/50.4)
Imipenem[f]	964	16 (65.6/NA)	1052	16 (72.6/NA)	596	16 (73.0/NA)	728	16 (67.9/NA)	485	16 (79.8/NA)
Levofloxacin	1023	16 (17.7/28)	1105	16 (16.4/20.4)	619	16 (20.2/29.4)	819	16 (18.0/24.9)	526	16 (22.4/29.7)
Meropenem	1023	32 (69.4/73.7)	1105	32 (76.9/79.5)	619	32 (76.4/80.6)	819	32 (73.0/75.3)	526	32 (82.5/85.6)
Piperacillin/tazobactam	1023	256 (36.5/36.5)	1105	128 (53.6/53.6)	619	256 (50.4/50.4)	819	128 (53.7/53.7)	526	128 (60.3/60.3)

Tigecycline[g,h,i]	1005	2 (95.2/97.5)	1061	2 (97.6/95.1)	597	2 (94.6/96.6)	797	2 (97.7/96.3)	518	1 (97.7/96.8)
**Europe (N=8807)**										
Aztreonam-avibactam[c]	1819	0.25 (99.9)	1758	0.25 (99.8)	1320	0.25 (99.9)	1330	0.25 (100)	975	0.5 (99.9)
Aztreonam	1819	256 (8.4/8.4)	1758	256 (10.1/10.1)	1320	256 (11.5/11.5)	1330	256 (6.5/6.5)	975	256 (9.2/9.2)
Amikacin	2148	32 (87.2/81.1)	2154	16 (91.2/86.6)	1491	16 (90.9/86.2)	1735	16 (91.4/85)	1174	16 (90.7/85.0)
Cefepime	2148	64 (11.7/16.2)	2154	64 (12.0/18.0)	1491	64 (14.8/20.5)	1735	64 (7.2/12.2)	1174	64 (15.3/20.8)
Ceftazidime	2148	256 (13.6/13.6)	2154	256 (17.2/17.2)	1491	256 (19.1/19.1)	1735	256 (15.5/15.5)	1174	256 (17.2/17.2)
Ceftazidime-avibactam	1819	2 (92.7/92.7)	1758	2 (94.6/94.6)	1320	2 (95.5/95.5)	1330	2 (94.4/94.4)	975	2 (94.5/94.5)
Ceftriaxone	1263	64 (3.6/5.3)	1320	64 (3.3/4.6)	917	64 (4.7/6.8)	731	64 (2.7/3.8)	656	64 (4.6/6.3)
Ciprofloxacin	885	8 (9.3/14.8)	834	8 (11.8/16.4)	574	8 (11.9/16.0)	1004	8 (10.3/14.7)	518	8 (13.9/19.9)
Colistin[d,e]	1764	1 (NA/92.4)	1700	1 (NA/95.1)	1238	1 (NA/94.6)	1292	1 (NA/93.7)	955	1 (NA/94.8)
Gentamicin	885	32 (43.7/43.2)	834	32 (48.9/48)	574	32 (44.6/44.3)	1004	32 (44.4/44)	518	32 (56.8/55.8)
Imipenem[f]	1819	16 (72.4/NA)	1758	8 (81.1/NA)	1320	8 (76.4/NA)	1330	16 (75/NA)	975	8 (77.6/NA)
Levofloxacin	2148	16 (23.5/33.1)	2154	16 (22.5/30.8)	1491	16 (23.3/32.5)	1735	16 (18.7/26.5)	1174	16 (27.8/34.6)
Meropenem	2148	16 (77.9/85.6)	2154	8 (86.2/91.2)	1491	8 (81.2/90.5)	1735	16 (79.5/86.6)	1174	16 (81.0/88.1)
Piperacillin/tazobactam	2148	256 (36.2/36.2)	2154	256 (51.3/51.3)	1491	256 (46.1/46.1)	1735	128 (44.7/44.7)	1174	256 (46.4/46.4)
Tigecycline[g,h,i]	2101	2 (96.4/98.3)	2097	2 (97.1/98.5)	1412	1 (96.4/98.2)	1702	2 (96.0/98.7)	1155	1 (96.4/97.3)
**Latin America (N=4115)**										
Aztreonam-avibactam[c]	586	0.25 (100)	933	0.25 (100)	678	0.5 (99.9)	827	0.25 (100)	679	0.25 (100)
Aztreonam	586	256 (5.5/5.5)	933	256 (8.5/8.5)	678	256 (10.2/10.2)	827	256 (4.8/4.8)	679	256 (8.1/8.1)
Amikacin	637	16 (90.3/83.7)	999	16 (91.2/85.3)	751	16 (91/84.6)	906	32 (86.5/80.9)	798	16 (92.6/86.3)
Cefepime	637	64 (8.2/14.1)	999	64 (7.9/13.5)	751	64 (9.1/14.4)	906	64 (5.4/10.3)	798	64 (9.7/14.9)
Ceftazidime	637	256 (11.8/11.8)	999	256 (18.2/18.2)	751	256 (14.5/14.5)	906	256 (11.7/11.7)	798	256 (16.9/16.9)
Ceftazidime-avibactam	586	2 (94.0/94.0)	933	2 (95.4/95.4)	678	16 (90.0/90.0)	827	4 (92.0/92.0)	679	2 (95.7/95.7)
Ceftriaxone	289	64 (2.8/2.8)	497	64 (2.4/4.2)	425	64 (2.4/5.7)	329	64 (2.1/2.4)	471	64 (2.3/2.3)
Ciprofloxacin	348	8 (5.8/9.5)	502	8 (8.6/11.8)	326	8 (8.0/14.4)	577	8 (9.4/15.4)	327	8 (9.8/14.4)
Colistin[d,e]	573	1 (NA/92.8)	900	1 (NA/96.0)	642	0.5 (NA/96.9)	808	1 (NA/94.8)	670	1 (NA/95.7)
Gentamicin	348	32 (37.9/37.4)	502	32 (40.0/39.0)	326	32 (31.3/30.4)	577	32 (38.7/37.8)	327	32 (42.5/41.3)
Imipenem[f]	586	16 (76.1/NA)	933	16 (81.7/NA)	678	16 (79.1/NA)	827	16 (77.0/NA)	679	8 (83.8/NA)
Levofloxacin	637	16 (20.1/32.3)	999	16 (16.2/23.6)	751	16 (19.7/30.2)	906	16 (21.2/34.6)	798	16 (19.9/26.7)
Meropenem	637	16 (79.3/85.6)	999	16 (83.4/89.3)	751	16 (82.0/88.3)	906	16 (77.4/84.6)	798	8 (85.6/91.1)
Piperacillin/tazobactam	637	128 (43.3/43.3)	999	256 (51.0/51.0)	751	256 (51.1/51.1)	906	128 (44.9/44.9)	798	256 (52.0/52.0)
Tigecycline[g,h,i]	628	1 (96.7/98.6)	970	1 (98.3/98.4)	720	1 (97.8/97.1)	893	1 (97.8/99.1)	789	1 (97.7/97.2)
**North America (N=1099)**										
Aztreonam-avibactam[c]	225	0.25 (99.6)	220	0.25 (100)	123	0.5 (100)	237	0.25 (100)	104	0.5 (100)
Aztreonam	225	128 (9.8/9.8)	220	128 (17.3/17.3)	123	128 (17.1/17.1)	237	128 (9.3/9.3)	104	128 (14.4/14.4)
Amikacin	265	16 (92.5/84.2)	270	8 (97.0/92.2)	137	8 (99.3/92.7)	300	8 (97.3/93.3)	120	8 (95.0/93.3)
Cefepime	265	64 (21.9/28.3)	270	64 (22.6/31.5)	137	64 (25.6/38.7)	300	64 (10.0/16.3)	120	64 (25.0/33.3)

Ceftazidime	265	128 (14.3/14.3)	270	128 (24.8/24.8)	137	128 (23.4/23.4)	300	128 (18.3/18.3)	120	128 (23.3/23.3)
Ceftazidime-avibactam	225	2 (97.3/97.3)	220	1 (98.2/98.2)	123	1 (97.6/97.6)	237	1 (100/100)	104	2 (97.1/97.1)
Ceftriaxone	126	64 (10.3/11.9)	132	64 (7.6/11.4)	72	64 (16.7/19.4)	82	64 (1.2/3.7)	69	64 (8.7/11.6)
Ciprofloxacin	139	8 (12.2/19.4)	138	8 (13.8/20.3)	65	8 (10.8/16.9)	218	8 (10.1/17.9)	51	8 (15.7/19.6)
Colistin[d,e]	220	0.5 (NA/96.4)	205	0.5 (NA/97.6)	114	0.5 (NA/97.4)	234	0.5 (NA/98.3)	103	0.5 (NA/99.0)
Gentamicin	139	32 (54.7/52.5)	138	32 (65.2/64.5)	65	32 (53.9/53.9)	218	32 (57.3/56.9)	51	32 (54.9/54.9)
Imipenem[f]	225	4 (84.9/NA)	220	2 (88.2/NA)	123	2 (88.6/NA)	237	1 (92.4/95.8)	104	2 (84.6/NA)
Levofloxacin	265	16 (28.3/39.3)	270	16 (27.8/35.6)	137	16 (32.1/42.3)	300	16 (20.7/28.0)	120	16 (25.8/27.5)
Meropenem	265	2 (89.4/93.6)	270	0.25 (94.4/98.2)	137	0.25 (94.2/98.5)	300	0.12 (95.3/98.0)	120	4 (87.5/92.5)
Piperacillin/tazobactam	265	128 (49.4/49.4)	270	128 (66.7/66.7)	137	128 (61.3/61.3)	300	128 (68.0/68.0)	120	256 (53.3/53.3)
Tigecycline[g,h,i]	260	1 (98.1/99.0)	256	1 (98.1/94.6)	128	1 (98.4/100)	297	1 (98.0/98.4)	119	1 (98.3/94.4)

[a] Not all drugs in the panel were tested every year. [b] Data includes percentage isolates susceptible at increased exposure. [c] No breakpoints available from CLSI and EUCAST. Values expressed are indicative of the cumulative percentage of isolates inhibited at ≤8 mg/L for comparison purposes. [d] Susceptible category for colistin not available for CLSI breakpoints (only intermediate and resistant isolates are available). [e] Data for colistin do not include isolates of *Morganella morganii, Proteus hauseri, Proteus mirabilis, Proteus vulgaris, Providencia alcalifaciens, Providencia rettgeri, Providencia* spp, *Providencia stuartii*, and *Serratia marcescens* because of their intrinsic resistance. [f] Data for imipenem not available per EUCAST. [g] Data for tigecycline do not include isolates of *Morganella morganii, Proteus hauseri, Proteus mirabilis, Proteus vulgaris, Providencia alcalifaciens, Providencia rettgeri, Providencia* spp, and *Providencia stuartii* due to their intrinsic resistance. [h] Data for tigecycline was calculated based on FDA approved breakpoints for CLSI. [i] EUCAST data for susceptibility to tigecycline are limited to *E. coli* and *C. koseri;* denominator (n): AfME: RTI=85, UTI=246, SSTI=218, BSI=152, IAI=101; APAC: RTI= 240, UTI=628, SSTI=290, BSI=435, IAI=312; Europe: RTI=457, UTI=732, SSTI=509, BSI=623, IAI=509; LATAM: RTI=141, UTI= 425, SSTI=350, BSI=315, IAI=435; North America: RTI= 100, UTI=148, SSTI=65, BSI=186, IAI=71. BSI, bloodstream infections; IAI, intra-abdominal infection; MIC, minimum inhibitory concentration; N, total number of isolates; n, number of isolates from infection sources; NA, not available; RTI, respiratory tract infection; SSTI, skin and soft tissue infection; UTI, urinary tract infection.

Table S7. *In vitro* activity of ATM-AVI and comparator agents tested against carbapenem-resistant Enterobacterales (CRE) isolates across regions stratified by wards from 2016-2020.

	**ICU**				**Non-ICU**			
	**CLSI**		**EUCAST**		**CLSI**		**EUCAST**	
	**n[a]**	**MIC_{90} (µg/mL) (% S)**	**n[a]**	**MIC_{90} (mg/L)/ (% S[b])**	**n[a]**	**MIC_{90} (µg/mL)/ (% S)**	**n[a]**	**MIC_{90} (mg/L)/ (% S[b])**
**Africa-Middle East (CLSI/EUCAST, N=337/252)**								
Aztreonam-avibactam[c]	105	0.5 (100)	83	0.5 (100)	172	0.5 (100)	118	0.5 (100)
Aztreonam	105	256 (13.3)	83	256 (9.6)	172	256 (16.9)	118	256 (16.1)
Amikacin	115	128 (59.1)	89	128 (44.9)	176	128 (62.5)	121	128 (41.3)
Cefepime	115	64 (1.7)	89	64 (3.4)	176	64 (5.7)	121	64 (3.3)
Ceftazidime	115	256 (3.5)	89	256 (4.5)	176	256 (4.6)	121	256 (4.1)
Ceftazidime-avibactam	105	256 (46.7)	83	256 (49.4)	172	256 (42.4)	118	256 (40.7)
Ceftriaxone	21	64 (0)	14	64 (0)	32	64 (0)	26	64 (3.9)
Ciprofloxacin	94	8 (8.5)	75	8 (10.7)	144	8 (4.9)	95	8 (10.5)
Colistin[d,e]	95	2 (NA)	79	2 (92.4)	156	1 (NA)	111	1 (96.4)

Gentamicin	94	32 (28.7)	75	32 (29.3)	144	32 (36.8)	95	32 (33.7)
Imipenem[f]	105	16 (1.0)	83	16 (13.3)	172	16 (2.3)	118	16 (8.5)
Levofloxacin	115	16 (15.7)	89	16 (16.9)	176	16 (11.4)	121	16 (12.4)
Meropenem	115	32 (0)	89	32 (0)	176	32 (0)	121	32 (0)
Piperacillin/tazobactam	115	256 (0)	89	256 (0)	176	256 (0)	121	256 (0)
Tigecycline[g,h,i]	112	2 (92.0)	88	2 (100)	162	2 (94.4)	114	2 (100)
**Asia-Pacific (CLSI/EUCAST, N=1691/1531)**								
Aztreonam-avibactam[c]	499	2 (96.8)	461	2 (97.4)	594	2 (98.7)	530	2 (98.7)
Aztreonam	626	256 (6.6)	577	256 (5.9)	785	256 (9.6)	709	256 (8.2)
Amikacin	653	128 (28.6)	602	128 (22.3)	831	128 (48.9)	744	128 (40.9)
Cefepime	653	64 (0.2)	602	64 (0.2)	831	64 (0.8)	744	64 (0.9)
Ceftazidime	653	256 (1.4)	602	256 (1.0)	831	256 (2.2)	744	256 (1.9)
Ceftazidime-avibactam	626	256 (42.2)	577	256 (42.3)	785	256 (46.2)	709	256 (45.4)
Ceftriaxone	86	64 (0)	71	64 (0)	184	64 (0)	145	64 (0)
Ciprofloxacin	567	8 (2.5)	531	8 (3.0)	647	8 (3.6)	599	8 (5.0)
Colistin[d,e]	570	4 (NA)	532	4 (89.9)	735	2 (NA)	668	2 (91.2)
Gentamicin	567	32 (16.4)	531	32 (15.1)	647	32 (28.3)	599	32 (26.0)
Imipenem[f]	626	16 (0.6)	577	16 (NA)	785	16 (2.9)	709	16 (5.1)
Levofloxacin	653	16 (4.4)	602	16 (5.8)	831	16 (6.6)	744	16 (8.1)
Meropenem	653	32 (0)	602	32 (0)	831	32 (0)	744	32 (0)
Piperacillin/tazobactam	653	256 (1.2)	602	256 (0.3)	831	256 (0.8)	744	256 (0.3)
Tigecycline[g,h,i]	612	2 (93.3)	569	2 (89.9)	805	2 (93.2)	724	2 (81.5)
**Europe (CLSI/EUCAST, N=2241/1649)**								
Aztreonam-avibactam[c]	701	0.5 (99.7)	550	0.5 (99.6)	971	0.5 (99.7)	692	0.5 (99.7)
Aztreonam	701	256 (10.7)	550	256 (8.7)	971	256 (9.1)	692	256 (7.1)
Amikacin	824	128 (56.9)	637	128 (33.3)	1120	128 (62.9)	801	128 (40.3)
Cefepime	824	64 (3.6)	637	64 (4.1)	1120	64 (3.0)	801	64 (2.6)
Ceftazidime	824	256 (5.0)	637	256 (4.2)	1120	256 (3.8)	801	256 (2.4)
Ceftazidime-avibactam	701	256 (68.5)	550	256 (67.5)	971	256 (72.7)	692	256 (72.0)
Ceftriaxone	306	64 (1.0)	226	64 (0)	461	64 (0.9)	315	64 (0.6)
Ciprofloxacin	518	8 (4.3)	411	8 (4.1)	659	8 (5.2)	486	8 (3.5)
Colistin[d,e]	657	16 (NA)	517	16 (74.5)	917	16 (NA)	667	16 (77.2)
Gentamicin	518	32 (34.2)	411	32 (28.7)	659	32 (37.9)	486	32 (33.7)
Imipenem[f]	701	16 (1.6)	550	16 (7.1)	971	16 (3.4)	692	16 (5.9)
Levofloxacin	824	16 (7.2)	637	16 (5.0)	1120	16 (6.3)	801	16 (5.4)
Meropenem	824	32 (0)	637	32 (0)	1120	32 (0)	801	32 (0)
Piperacillin/tazobactam	824	256 (0.4)	637	256 (0.5)	1120	256 (1.3)	801	256 (0.5)
Tigecycline[g,h,i]	797	2 (93.9)	617	2 (90.0)	1076	2 (93.4)	782	2 (81.0)
**Latin America (CLSI/EUCAST, N=1133/864)**								
Aztreonam-avibactam[c]	379	0.5 (100)	292	0.5 (100)	461	0.5 (99.8)	332	0.5 (99.7)
Aztreonam	379	256 (6.9)	292	256 (5.1)	461	256 (9.8)	332	256 (8.7)
Amikacin	425	128 (66.4)	333	128 (48.7)	540	128 (69.8)	398	128 (51.3)
Cefepime	425	64 (2.1)	333	64 (1.2)	540	64 (2.6)	398	64 (1.3)
Ceftazidime	425	256 (3.3)	333	256 (1.5)	540	256 (6.3)	398	256 (3.3)
Ceftazidime-avibactam	379	256 (72.3)	292	256 (73.0)	461	256 (69.2)	332	256 (66.3)

Ceftriaxone	154	64 (0)	127	64 (0)	201	64 (0.5)	152	64 (0.7)
Ciprofloxacin	271	8 (13.7)	206	8 (11.7)	339	8 (10.0)	246	8 (7.3)
Colistin[d,e]	350	16 (NA)	273	16 (75.5)	427	16 (NA)	308	16 (77.0)
Gentamicin	271	32 (36.2)	206	32 (34.0)	339	32 (43.1)	246	32 (37.8)
Imipenem[f]	379	16 (1.3)	292	16 (2.4)	461	16 (2)	332	16 (1.8)
Levofloxacin	425	16 (14.6)	333	16 (16.5)	540	16 (15.7)	398	16 (18.1)
Meropenem	425	32 (0)	333	32 (0)	540	32 (0)	398	32 (0)
Piperacillin/tazobactam	425	256 (0.7)	333	256 (0.6)	540	256 (0.9)	398	256 (0.5)
Tigecycline[g,h,i]	411	2 (94.4)	327	2 (87.5)	522	2 (95.6)	386	2 (85.0)
**North America (CLSI/EUCAST, N=174/92)**								
Aztreonam-avibactam[c]	31	0.5 (100)	15	0.5 (100)	90	1 (97.8)	48	1 (97.9)
Aztreonam	31	256 (6.5)	15	256 (0)	90	256 (12.2)	48	256 (12.5)
Amikacin	41	32 (82.9)	20	64 (70.0)	104	64 (81.7)	56	128 (60.7)
Cefepime	41	64 (9.8)	20	64 (10.0)	104	64 (7.7)	56	64 (7.1)
Ceftazidime	41	256 (7.3)	20	256 (10.0)	104	256 (12.5)	56	256 (10.7)
Ceftazidime-avibactam	31	256 (83.9)	15	128 (86.7)	90	256 (82.2)	48	256 (77.1)
Ceftriaxone	13	64 (7.7)	8	NA[j] (12.5)	26	64 (7.7)	13	64 (7.7)
Ciprofloxacin	28	8 (17.9)	12	8 (16.7)	78	8 (15.4)	43	8 (14.0)
Colistin[d,e]	29	8 (NA)	13	8 (84.6)	82	1 (NA)	42	1 (90.5)
Gentamicin	28	32 (60.7)	12	32 (50.0)	78	32 (44.9)	43	32 (37.2)
Imipenem[f]	31	16 (6.5)	15	16 (NA)	90	16 (8.9)	48	16 (14.6)
Levofloxacin	41	16 (24.4)	20	16 (0)	104	16 (19.2)	56	16 (14.3)
Meropenem	41	32 (0)	20	32 (20.0)	104	32 (0)	56	32 (0)
Piperacillin/tazobactam	41	256 (4.9)	20	256 (0)	104	256 (2.9)	56	256 (3.6)
Tigecycline[g,h,i]	41	2 (95.1)	20	2 (NA)	103	2 (90.3)	56	2 (0)

[a] Data includes percentage isolates susceptible at increased exposure. [b] Not all drugs in the panel were tested every year. [c] No breakpoints available from CLSI and EUCAST. Values expressed are indicative of the cumulative percentage of isolates inhibited at ≤8 mg/L for comparison purposes. [d] Susceptible category for colistin not available for CLSI breakpoints (only intermediate and resistant isolates are available). [e] Data for colistin do not include isolates of *Morganella morganii*, *Proteus hauseri*, *Proteus mirabilis*, *Proteus vulgaris*, *Providencia alcalifaciens*, *Providencia rettgeri*, *Providencia* spp, *Providencia stuartii*, and *Serratia marcescens* because of their intrinsic resistance. [f] Data for imipenem not available per EUCAST. [g] Data for tigecycline do not include isolates of *Morganella morganii*, *Proteus hauseri*, *Proteus mirabilis*, *Proteus vulgaris*, *Providencia alcalifaciens*, *Providencia rettgeri*, *Providencia* spp, and *Providencia stuartii* due to their intrinsic resistance. [h] Data for tigecycline was calculated based on FDA approved breakpoints for CLSI. [i] EUCAST data for susceptibility to tigecycline are limited to *E. coli* and *C. Koseri*; denominator (n): AfME: ICU= 2, non-ICU= 3; APAC: ICU= 79, non-ICU= 135; Europe: ICU= 10, non-ICU= 21; LATAM: ICU= 8, non-ICU= 20; North America: ICU= NA, non-ICU= 2. [j] The MIC_{90} has been presented as NA considering the n<10. ICU, intensive care unit; MIC, minimum inhibitory concentration; N, total number of isolates; n, number of isolates from wards; NA, not available.

Table S8. *In vitro* activity of ATM-AVI and comparator agents tested against carbapenem-resistant Enterobacterales (CRE) isolates across regions stratified by infection sources from 2016-2020.

	RTI				UTI				SSTI			
	CLSI		EUCAST		CLSI		EUCAST		CLSI		EUCAST	
	n[a]	MIC_{90} (µg/mL) (% S)	n[a]	MIC_{90} (mg/L) (% S[b])	n[a]	MIC_{90} (µg/mL) (% S)	n[a]	MIC_{90} (mg/L) (% S[b])	n[a]	MIC_{90} (µg/mL) (% S)	n[a]	MIC_{90} (mg/L) (% S[b])
**Africa-Middle East (CLSI/EUCAST, N=337/252)**												
Aztreonam-avibactam[c]	63	0.5 (100)	50	0.5 (100)	74	1 (100)	49	1 (100)	59	0.5 (98.3)	44	0.5 (97.7)
Aztreonam	63	256 (17.5)	50	256 (14.0)	74	256 (23.0)	49	256 (26.5)	59	256 (10.2)	44	256 (11.4)
Amikacin	68	128 (60.3)	54	128 (44.4)	76	128 (75.0)	51	128 (60.8)	63	128 (52.4)	44	128 (40.9)
Cefepime	68	64 (5.9)	54	64 (3.7)	76	64 (1.3)	51	64 (3.9)	63	64 (3.2)	44	64 (2.3)
Ceftazidime	68	256 (10.3)	54	256 (9.3)	76	256 (1.3)	51	256 (2.0)	63	256 (1.6)	44	256 (0)
Ceftazidime-avibactam	63	256 (65.1)	50	256 (60.0)	74	256 (23.0)	49	256 (18.4)	59	256 (35.6)	44	256 (40.9)
Ceftriaxone	16	64 (0)	12	64 (0)	8	NA[d] (0)	5	NA[d] (0)	15	64 (0)	10	32 (0)
Ciprofloxacin	52	8 (7.7)	42	8 (4.8)	68	8 (2.9)	46	8 (6.5)	48	8 (4.2)	34	8 (11.8)
Colistin[e,f]	60	1 (NA)	49	1 (93.9)	59	8 (NA)	41	16 (85.4)	54	1 (NA)	40	2 (92.5)
Gentamicin	52	32 (38.5)	42	32 (38.1)	68	32 (26.5)	46	32 (26.1)	48	32 (33.3)	34	32 (26.5)
Imipenem[g]	63	16 (4.8)	50	16 (18)	74	16 (1.4)	49	16 (6.1)	59	16 (1.7)	44	16 (9.1)
Levofloxacin	68	16 (13.2)	54	16 (11.1)	76	16 (15.8)	51	16 (19.6)	63	16 (9.5)	44	16 (13.6)
Meropenem	68	32 (0)	54	32 (0)	76	32 (0)	51	32 (0)	63	32 (0)	44	32 (0)
Piperacillin/tazobactam	68	256 (0)	54	256 (0)	76	128 (0)	51	128 (0)	63	256 (0)	44	256 (0)
Tigecycline[h,i,j]	67	4 (89.6)	54	2 (NA)	65	2 (95.4)	45	2 (100)	58	2 (93.1)	40	4 (100)
**Asia-Pacific (CLSI/EUCAST, N=1691/1531)**												
Aztreonam-avibactam[c]	375	2 (97.9)	335	2 (97.9)	303	4 (97.7)	271	4 (97.4)	160	2 (97.5)	140	2 (98.6)
Aztreonam	503	256 (5.2)	451	256 (3.3)	380	256 (11.3)	342	256 (9.1)	192	256 (9.9)	172	256 (8.1)
Amikacin	536	128 (39.9)	479	128 (32.4)	398	128 (34.4)	356	128 (26.1)	206	128 (46.1)	184	128 (36.4)
Cefepime	536	64 (0.6)	479	64 (0.8)	398	64 (1.3)	356	64 (0.6)	206	64 (0.5)	184	64 (0.5)
Ceftazidime	536	256 (2.1)	479	256 (1.3)	398	256 (1.5)	356	256 (0.8)	206	256 (2.9)	184	256 (2.2)
Ceftazidime-avibactam	503	256 (56.1)	451	256 (56.8)	380	256 (35.5)	342	256 (33.9)	192	256 (38.5)	172	256 (37.8)
Ceftriaxone	122	64 (0)	98	64 (0)	74	64 (0)	56	64 (0)	45	64 (0)	36	64 (0)
Ciprofloxacin	414	8 (2.7)	381	8 (3.2)	324	8 (2.8)	300	8 (2.7)	161	8 (5.0)	148	8 (0)
Colistin[e,f]	470	4 (NA)	428	2 (90.4)	338	2 (NA)	307	1 (92.5)	180	2 (NA)	164	2 (92.1)
Gentamicin	414	32 (22.7)	381	32 (20.0)	324	32 (15.1)	300	32 (14.0)	161	32 (26.1)	148	32 (88.4)
Imipenem[g]	503	16 (1.0)	451	16 (2.7)	380	16 (2.1)	342	16 (6.1)	192	16 (0.5)	172	16 (23.0)
Levofloxacin	536	16 (5.4)	479	16 (6.9)	398	16 (4.8)	356	16 (4.8)	206	16 (7.8)	184	16 (9.2)
Meropenem	536	32 (0)	479	32 (0)	398	32 (0)	356	32 (0)	206	32 (0)	184	32 (0)
Piperacillin/tazobactam	536	256 (1.3)	479	256 (0)	398	256 (1.3)	356	256 (0)	206	256 (1.5)	184	256 (0.5)
Tigecycline[h,i,j]	528	2 (92.6)	477	2 (76.7)	362	2 (93.7)	325	2 (86.6)	195	4 (89.2)	177	4 (88.5)

**Europe (CLSI/EUCAST, N=2241/1649)**												
Aztreonam-avibactam[c]	530	0.5 (99.6)	397	0.5 (99.5)	345	0.5 (99.7)	253	0.5 (99.6)	331	0.5 (99.7)	215	1 (99.5)
Aztreonam	530	256 (9.3)	397	256 (6.8)	345	256 (14.8)	253	256 (13.0)	331	256 (12.1)	215	256 (9.8)
Amikacin	607	128 (57.5)	456	128 (38.2)	394	128 (61.4)	286	128 (37.8)	381	128 (66.7)	244	128 (42.2)
Cefepime	607	64 (3.6)	456	64 (2.9)	394	64 (3.1)	286	64 (2.5)	381	64 (4.2)	244	64 (3.7)
Ceftazidime	607	256 (5.3)	456	256 (3.5)	394	256 (4.3)	286	256 (2.8)	381	256 (6)	244	256 (3.7)
Ceftazidime-avibactam	530	256 (69.3)	397	256 (67.0)	345	256 (63.5)	253	256 (61.3)	331	256 (76.1)	215	256 (74.0)
Ceftriaxone	254	64 (1.2)	187	64 (0.5)	151	64 (1.3)	102	64 (1.0)	168	64 (1.2)	99	64 (1.0)
Ciprofloxacin	353	8 (4.3)	269	8 (4.1)	243	8 (4.9)	184	8 (2.2)	213	8 (5.6)	145	8 (4.8)
Colistin[e,f]	505	16 (NA)	381	16 (73.8)	316	16 (NA)	235	16 (72.3)	307	16 (NA)	199	16 (75.9)
Gentamicin	353	32 (35.4)	269	32 (31.6)	243	32 (37.5)	184	32 (30.4)	213	32 (41.8)	145	32 (40.0)
Imipenem[g]	530	16 (2.1)	397	16 (6.3)	345	16 (3.8)	253	16 (8.7)	331	16 (4.5)	215	16 (6.5)
Levofloxacin	607	16 (6.4)	456	16 (4.8)	394	16 (6.9)	286	16 (7.7)	381	16 (8.9)	244	16 (5.7)
Meropenem	607	32 (0)	456	32 (0)	394	32 (0)	286	32 (0)	381	32 (0)	244	32 (0)
Piperacillin/tazobactam	607	256 (1.2)	456	256 (0.7)	394	256 (1.3)	286	256 (1.1)	381	256 (1.1)	244	256 (0)
Tigecycline[h,i,j]	594	2 (93.9)	448	2 (71.4)	368	2 (93.8)	270	2 (90.0)	362	2 (92.5)	231	2 (62.5)
**Latin America (CLSI/EUCAST, N=1133/864)**												
Aztreonam-avibactam[c]	180	0.5 (100)	143	0.5 (100)	214	0.5 (100)	160	0.5 (100)	167	0.5 (100)	119	0.5 (100)
Aztreonam	180	256 (5.0)	143	256 (4.2)	214	256 (15.4)	160	256 (13.8)	167	256 (13.2)	119	256 (12.6)
Amikacin	207	128 (71.0)	168	128 (58.3)	241	128 (63.9)	181	128 (49.7)	187	128 (65.2)	136	128 (44.1)
Cefepime	207	64 (3.4)	168	64 (1.8)	241	64 (2.9)	181	64 (1.1)	187	64 (2.7)	136	64 (2.9)
Ceftazidime	207	256 (1.9)	168	256 (1.2)	241	256 (4.6)	181	256 (2.8)	187	256 (8.0)	136	256 (4.4)
Ceftazidime-avibactam	180	128 (78.9)	143	128 (78.3)	214	256 (67.8)	160	256 (67.5)	167	256 (53.9)	119	256 (47.1)
Ceftriaxone	63	64 (0)	53	64 (0)	94	64 (0)	69	64 (0)	70	64 (1.4)	51	64 (2.0)
Ciprofloxacin	144	8 (4.2)	115	8 (4.4)	147	8 (8.2)	112	8 (5.4)	117	8 (11.1)	85	8 (12.9)
Colistin[e,f]	167	16 (NA)	133	16 (72.9)	191	16 (NA)	145	16 (79.3)	153	8 (NA)	108	16 (78.7)
Gentamicin	144	32 (39.6)	115	32 (34.8)	147	32 (29.3)	112	32 (24.1)	117	32 (33.3)	85	32 (25.9)
Imipenem[g]	180	16 (1.1)	143	16 (1.4)	214	16 (2.8)	160	16 (1.9)	167	16 (3.6)	119	16 (3.4)
Levofloxacin	207	16 (7.7)	168	16 (10.1)	241	16 (10.8)	181	16 (14.9)	187	16 (18.7)	136	16 (27.9)
Meropenem	207	32 (0)	168	32 (0)	241	32 (0)	181	32 (0)	187	32 (0)	136	32 (0)
Piperacillin/tazobactam	207	256 (0.5)	168	256 (0.6)	241	256 (0.4)	181	256 (0.6)	187	256 (1.6)	136	256 (0.7)
Tigecycline[h,i,j]	202	2 (92.6)	165	2 (75.0)	223	2 (98.2)	169	2 (75.0)	177	2 (97.7)	129	2 (90.0)
**North America (CLSI/EUCAST, N=174/92)**												
Aztreonam-avibactam[c]	52	1 (100)	30	1 (100)	31	1 (100)	16	1 (100)	30	4 (96.7)	10	4 (100)
Aztreonam	52	256 (11.5)	30	256 (10.0)	31	256 (25.8)	16	256 (31.3)	30	256 (20.0)	10	256 (20.0)
Amikacin	57	64 (77.2)	33	128 (57.6)	33	64 (69.7)	18	64 (61.1)	33	16 (90.9)	12	16 (75.0)
Cefepime	57	64 (14.0)	33	64 (12.1)	33	64 (9.1)	18	64 (11.1)	33	64 (3.0)	12	64 (0)
Ceftazidime	57	256 (14.0)	33	256 (15.2)	33	256 (15.2)	18	256 (22.2)	33	256 (15.2)	12	256 (16.7)

Ceftazidime-avibactam	52	128 (86.5)	30	128 (83.3)	31	256 (80.7)	16	256 (75.0)	30	256 (76.7)	10	256 (80.0)
Ceftriaxone	10	64 (10.0)	7	64 (14.3)	4	64 (50.0)	2	64 (50.0)	5	64 (0)	2	64 (0)
Ciprofloxacin	47	8 (12.8)	26	8 (7.7)	29	8 (0)	16	8 (6.3)	28	8 (21.4)	10	8 (30.0)
Colistin[e,f]	46	8 (NA)	24	8 (87.5)	27	2 (NA)	15	2 (93.3)	28	1 (NA)	8	1 (100)
Gentamicin	47	32 (46.8)	26	32 (34.6)	29	32 (44.8)	16	32 (37.5)	28	32 (64.3)	10	32 (50.0)
Imipenem[g]	52	16 (7.7)	30	16 (10.0)	31	16 (19.4)	16	16 (25.0)	30	16 (6.7)	10	16 (20.0)
Levofloxacin	57	16 (15.8)	33	16 (15.2)	33	16 (6.1)	18	16 (11.1)	33	16 (21.2)	12	16 (25.0)
Meropenem	57	32 (0)	33	32 (0)	33	32 (0)	18	32 (0)	33	32 (0)	12	32 (0)
Piperacillin/tazobactam	57	256 (7.0)	33	256 (12.1)	33	128 (12.1)	18	128 (11.1)	33	128 (0)	12	128 (0)
Tigecycline[h,i,j]	57	2 (94.7)	33	2 (100)	31	2 (90.3)	18	4 (NA)	33	4 (84.9)	12	2 (0)

[a] Not all drugs in the panel were tested every year. [b] Data includes percentage isolates susceptible at increased exposure. [c] No breakpoints available from CLSI and EUCAST. Values expressed are indicative of the cumulative percentage of isolates inhibited at ≤8 mg/L for comparison purposes. [d] The MIC_{90} has been presented as NA considering the n<10. [e] Susceptible category for colistin not available for CLSI breakpoints (only intermediate and resistant isolates are available). [f] Data for colistin do not include isolates of *Morganella morganii*, *Proteus hauseri*, *Proteus mirabilis*, *Proteus vulgaris*, *Providencia alcalifaciens*, *Providencia rettgeri*, *Providencia* spp, *Providencia stuartii*, and *Serratia marcescens* because of their intrinsic resistance. [g] Data for imipenem not available per EUCAST. [h] Data for tigecycline do not include isolates of *Morganella morganii*, *Proteus hauseri*, *Proteus mirabilis*, *Proteus vulgaris*, *Providencia alcalifaciens*, *Providencia rettgeri*, *Providencia* spp, and *Providencia stuartii* due to their intrinsic resistance. [i] Data for tigecycline was calculated based on FDA approved breakpoints for CLSI. [j] EUCAST data for susceptibility to tigecycline are limited to *E. coli* and *C. Koseri*; denominator (n): AfME: RTI= NA, UTI= 6, SSTI= 4; APAC: RTI= 43, UTI= 82, SSTI= 26; Europe: RTI= 7, UTI= 10, SSTI= 8; LATAM: RTI= 4, UTI= 4, SSTI= 10; North America: RTI= 1, UTI= NA, SSTI= 1. MIC, minimum inhibitory concentration; N, total number of isolates; n, number of isolates from infection sources; NA, not available; RTI, respiratory tract infection; SSTI, skin and soft tissue infection; UTI, urinary tract infection.

Table S8. *Cont.*

	**BSI**				**IAI**			
	**CLSI**		**EUCAST**		**CLSI**		**EUCAST**	
	**n[a]**	**MIC_{90} (µg/mL) (% S)**	**n[a]**	**MIC_{90} (mg/L) (% S[b])**	**n[a]**	**MIC_{90} (µg/mL) (% S)**	**n[a]**	**MIC_{90} (mg/L) (% S[b])**
**Africa-Middle East (CLSI/EUCAST, N=337/252)**								
Aztreonam-avibactam[c]	83	0.25 (100)	68	0.25 (100)	39	2 (100)	28	2 (100)
Aztreonam	83	256 (8.4)	68	256 (4.4)	39	256 (18.0)	28	256 (14.3)
Amikacin	88	128 (58.0)	73	128 (37.0)	41	128 (63.4)	30	128 (43.3)
Cefepime	88	64 (2.3)	73	64 (2.7)	41	64 (9.8)	30	64 (6.7)
Ceftazidime	88	256 (1.1)	73	256 (1.4)	41	256 (7.3)	30	256 (10.0)
Ceftazidime-avibactam	83	256 (32.5)	68	256 (32.4)	39	256 (66.7)	28	256 (67.9)
Ceftriaxone	15	64 (0)	14	64 (0)	10	64 (0)	9	NA[d] (11.1)
Ciprofloxacin	73	8 (9.6)	59	8 (15.3)	31	8 (3.2)	21	8 (9.5)
Colistin[e,f]	78	1 (NA)	66	1 (97.0)	34	1 (NA)	26	1 (100)
Gentamicin	73	32 (27.4)	59	32 (25.4)	31	32 (41.9)	21	32 (42.9)
Imipenem[g]	83	16 (1.2)	68	16 (7.4)	39	16 (0)	28	16 (7.1)
Levofloxacin	88	16 (19.3)	73	16 (23.3)	41	16 (2.4)	30	16 (6.7)
Meropenem	88	32 (0)	73	32 (0)	41	32 (0)	30	32 (0)
Piperacillin/tazobactam	88	256 (1.1)	73	256 (1.4)	41	256 (0)	30	256 (0)

Tigecycline[h,i,j]	86	2 (96.5)	71	2 (100)	37	2 (91.9)	29	4 (100)
**Asia-Pacific (CLSI/EUCAST, N=1691/1531)**								
Aztreonam-avibactam[c]	263	2 (98.5)	250	2 (99.2)	121	2 (99.2)	109	2 (99.1)
Aztreonam	344	256 (8.1)	327	256 (7.7)	163	256 (10.4)	147	256 (10.2)
Amikacin	365	128 (37.3)	346	128 (31.2)	172	128 (44.8)	153	128 (36.6)
Cefepime	365	64 (0)	346	64 (0)	172	64 (0.6)	153	64 (0.7)
Ceftazidime	365	256 (1.6)	346	256 (1.7)	172	256 (1.7)	153	256 (2.0)
Ceftazidime-avibactam	344	256 (39.0)	327	256 (38.8)	163	256 (49.1)	147	256 (48.3)
Ceftriaxone	35	64 (0)	33	64 (0)	36	64 (0)	29	64 (0)
Ciprofloxacin	330	8 (4.2)	313	8 (5.4)	136	8 (2.2)	124	8 (4.8)
Colistin[e,f]	320	4 (NA)	305	4 (89.5)	151	4 (NA)	139	8 (87.8)
Gentamicin	330	32 (26.1)	313	32 (23.3)	136	32 (25.7)	124	32 (25.8)
Imipenem[g]	344	16 (1.7)	327	16 (4.6)	163	16 (5.5)	147	16 (6.1)
Levofloxacin	365	16 (6.6)	346	16 (9.3)	172	16 (5.8)	153	16 (7.2)
Meropenem	365	32 (0)	346	32 (0)	172	32 (0)	153	32 (0)
Piperacillin/tazobactam	365	128 (0.3)	346	128 (0)	172	256 (1.7)	153	256 (2.0)
Tigecycline[h,i,j]	352	2 (95.7)	335	2 (88.9)	165	2 (93.3)	148	2 (84.6)
**Europe (CLSI/EUCAST, N=2241/1649)**								
Aztreonam-avibactam[c]	426	0.5 (99.5)	333	0.5 (99.7)	235	1 (100)	181	1 (100)
Aztreonam	426	256 (9.6)	333	256 (8.7)	235	256 (3.8)	181	256 (3.3)
Amikacin	533	128 (61)	417	128 (35.7)	302	64 (64.9)	231	128 (37.2)
Cefepime	533	64 (2.6)	417	64 (4.1)	302	64 (2.0)	231	64 (2.2)
Ceftazidime	533	256 (3.8)	417	256 (3.8)	302	256 (1.0)	231	256 (0.9)
Ceftazidime-avibactam	426	256 (74.7)	333	256 (76.9)	235	256 (73.6)	181	256 (74.0)
Ceftriaxone	176	64 (0)	136	64 (0.7)	151	64 (0)	115	64 (0)
Ciprofloxacin	357	8 (3.6)	281	8 (3.9)	151	8 (4.0)	116	8 (1.7)
Colistin[e,f]	398	16 (NA)	317	16 (76.3)	233	16 (NA)	181	16 (78.5)
Gentamicin	357	32 (34.5)	281	32 (29.2)	151	32 (37.8)	116	32 (32.8)
Imipenem[g]	426	16 (1.9)	333	16 (6.0)	235	16 (2.6)	181	16 (4.4)
Levofloxacin	533	16 (4.5)	417	16 (4.6)	302	16 (5.3)	231	16 (4.3)
Meropenem	533	32 (0)	417	32 (0)	302	32 (0)	231	32 (0)
Piperacillin/tazobactam	533	256 (0.8)	417	256 (0.5)	302	256 (0.3)	231	256 (0.4)
Tigecycline[h,i,j]	512	2 (92.0)	407	2 (100)	300	2 (92.7)	231	2 (85.7)
**Latin America (CLSI/EUCAST, N=1133/864)**								
Aztreonam-avibactam[c]	290	0.5 (99.7)	220	0.5 (99.6)	136	0.5 (100)	96	0.5 (100)
Aztreonam	290	256 (7.2)	220	256 (5.9)	136	256 (5.9)	96	256 (3.1)
Amikacin	321	64 (67.9)	248	128 (49.6)	164	64 (76.2)	122	64 (53.3)
Cefepime	321	64 (1.6)	248	64 (0.8)	164	64 (1.2)	122	64 (0.8)
Ceftazidime	321	256 (6.9)	248	256 (4.8)	164	256 (4.3)	122	256 (0.8)
Ceftazidime-avibactam	290	256 (71.7)	220	256 (71.4)	136	256 (72.1)	96	256 (70.8)
Ceftriaxone	90	64 (0)	70	64 (0)	74	64 (0)	65	64 (0)
Ciprofloxacin	231	8 (15.2)	178	8 (12.9)	90	8 (20.0)	57	8 (14.0)
Colistin[e,f]	262	16 (NA)	200	16 (78.5)	131	16 (0)	95	16 (74.7)
Gentamicin	231	32 (45.9)	178	32 (44.9)	90	32 (48.9)	57	32 (43.9)
Imipenem[g]	290	16 (1.4)	220	16 (4.1)	136	16 (1.5)	96	16 (1.0)

Levofloxacin	321	16 (19.6)	248	16 (21.8)	164	16 (17.1)	122	16 (19.7)
Meropenem	321	32 (0)	248	32 (0)	164	32 (0)	122	32 (0)
Piperacillin/tazobactam	321	256 (0.3)	248	256 (0.4)	164	256 (1.2)	122	256 (0)
Tigecycline[h,i,j]	313	2 (94.9)	244	2 (88.9)	160	2 (95.0)	122	2 (87.5)
**North America (CLSI/EUCAST, N=174/92)**								
Aztreonam-avibactam[c]	18	2 (94.5)	11	0.5 (90.9)	18	4 (100)	11	2 (100)
Aztreonam	18	256 (0)	11	256 (0)	18	256 (0)	11	256 (0)
Amikacin	21	32 (81.0)	12	64 (66.7)	27	128 (81.5)	15	128 (66.7)
Cefepime	21	64 (14.3)	12	64 (8.3)	27	64 (3.7)	15	64 (0)
Ceftazidime	21	256 (4.8)	12	256 (0)	27	256 (0)	15	256 (0)
Ceftazidime-avibactam	18	4 (94.4)	11	4 (90.9)	18	256 (83.3)	11	256 (81.8)
Ceftriaxone	4	NA[d] (0)	1	NA[d] (0)	15	64 (0)	9	NA[d] (0)
Ciprofloxacin	17	8 (23.5)	11	8 (18.2)	12	8 (25.0)	6	NA[d] (16.7)
Colistin[e,f]	18	2 (NA)	11	0.5 (90.9)	17	1 (NA)	10	8 (90)
Gentamicin	17	32 (47.1)	11	32 (45.5)	12	32 (41.7)	6	NA[d] (33.3)
Imipenem[g]	18	16 (5.6)	11	16 (9.1)	18	16 (0)	11	16 (0)
Levofloxacin	21	16 (28.6)	12	16 (16.7)	27	16 (22.2)	15	16 (6.7)
Meropenem	21	32 (0)	12	32 (0)	27	32 (0)	15	32 (0)
Piperacillin/tazobactam	21	128 (0)	12	128 (0)	27	256 (0)	15	256 (0)
Tigecycline[h,i,j]	21	2 (95.2)	12	2 (NA)	27	4 (88.9)	15	1 (0)

[a] Not all drugs in the panel were tested every year. [b] Data includes percentage isolates susceptible at increased exposure. [c] No breakpoints available from CLSI and EUCAST. Values expressed are indicative of the cumulative percentage of isolates inhibited at ≤8 mg/L for comparison purposes. [d] The MIC_{90} has been presented as NA considering the n<10. [e] Susceptible category for colistin not available for CLSI breakpoints (only intermediate and resistant isolates are available). [f] Data for colistin do not include isolates of *Morganella morganii*, *Proteus hauseri*, *Proteus mirabilis*, *Proteus vulgaris*, *Providencia alcalifaciens*, *Providencia rettgeri*, *Providencia* spp, *Providencia stuartii*, and *Serratia marcescens* because of their intrinsic resistance. [g] Data for imipenem not available per EUCAST. [h] Data for tigecycline do not include isolates of *Morganella morganii*, *Proteus hauseri*, *Proteus mirabilis*, *Proteus vulgaris*, *Providencia alcalifaciens*, *Providencia rettgeri*, *Providencia* spp, and *Providencia stuartii* due to their intrinsic resistance. [i] Data for tigecycline was calculated based on FDA approved breakpoints for CLSI. [j] EUCAST data for susceptibility to tigecycline are limited to *E. coli* and *C. Koseri*; denominator (n): AfME: BSI= 1, IAI= 1; APAC: BSI= 54, IAI=26; Europe: BSI= 2, IAI= 7; LATAM: BSI= 9, IAI=8; North America: BSI= NA, IAI=1. BSI, bloodstream infections; IAI, intra-abdominal infection; MIC, minimum inhibitory concentration; N, total number of isolates; n, number of isolates from infection sources; NA, not available.

Table S9. *In vitro* activity of ATM-AVI and comparator agents tested against MBL-positive Enterobacterales isolates across regions stratified by wards from 2016-2020.

	ICU		Non-ICU	
**Africa-Middle East (N= 190)**	**n[b]**	**MIC_{90} (μg/mL) (% S, CLSI/%S, EUCAST[a])**	**n[b]**	**MIC_{90} (μg/mL) (% S, CLSI/%S, EUCAST[a])**
Aztreonam-avibactam[c]	58	0.5 (100)	104	0.25 (100)
Aztreonam	58	256 (22.4/22.4)	104	128 (21.2/21.2)
Amikacin	58	128 (56.9/43.1)	104	128 (60.6/42.3)
Cefepime	58	64 (3.5/3.5)	104	64 (2.9/5.8)
Ceftazidime	58	256 (1.7/1.7)	104	256 (0/0)
Ceftazidime-avibactam	58	256 (3.5/3.5)	104	256 (1.9/1.9)
Ceftriaxone	4	NA[d] (0/0)	14	32 (0/0)
Ciprofloxacin	54	8 (16.7/16.7)	90	8 (6.7/15.6)

Colistin[e,f]	49	1 (NA/95.9)	88	1 (NA/96.6)
Gentamicin	54	32 (33.3/31.5)	90	32 (36.7/35.6)
Imipenem[g]	58	16 (1.7/10.3)	104	16 (1/NA)
Levofloxacin	58	16 (24.1/34.5)	104	16 (17.3/22.1)
Meropenem	58	32 (1.7/29.3)	104	32 (4.8/35.6)
Piperacillin/tazobactam	58	128 (1.7/1.7)	104	128 (1.0/1.0)
Tigecycline[h,i,j]	55	2 (94.6/100)	89	2 (96.6/100)
**Asia-Pacific (N= 735)**				
Aztreonam-avibactam[c]	285	4 (96.8)	302	4 (99.3)
Aztreonam	317	256 (8.8/8.8)	324	256 (14.5/14.5)
Amikacin	317	128 (27.8/20.8)	325	128 (51.7/43.7)
Cefepime	317	64 (0/0.6)	325	64 (0.3/1.2)
Ceftazidime	317	256 (0/0)	325	256 (0/0)
Ceftazidime-avibactam	317	256 (0.6/0.6)	324	256 (0.3/0.3)
Ceftriaxone	23	32 (0/0)	63	32 (0/0)
Ciprofloxacin	294	8 (3.7/5.4)	262	8 (3.1/6.1)
Colistin[e,f]	282	2 (NA/90.8)	305	1 (NA/93.4)
Gentamicin	294	32 (19.7/19.1)	262	32 (32.8/31.3)
Imipenem[g]	317	16 (1.6/NA)	324	16 (1.5/7.7)
Levofloxacin	317	16 (6.0/10.4)	325	16 (5.9/10.8)
Meropenem	317	32 (2.2/10.1)	325	32 (1.2/12.0)
Piperacillin/tazobactam	317	128 (1.9/1.9)	325	256 (2.2/2.2)
Tigecycline[h,i,j]	289	2 (94.8/92.5)	311	2 (92.9/92.0)
**Europe (N= 604)**				
Aztreonam-avibactam[c]	235	0.5 (100)	310	0.5 (99.7)
Aztreonam	235	256 (17.9/17.9)	310	256 (20.3/20.3)
Amikacin	235	128 (46.8/33.2)	311	128 (53.7/41.2)
Cefepime	235	64 (0.4/0.4)	311	64 (2.9/4.5)
Ceftazidime	235	256 (0/0)	311	256 (0/0)
Ceftazidime-avibactam	235	256 (4.3/4.3)	310	256 (6.5/6.5)
Ceftriaxone	43	32 (0/0)	93	32 (0/0)
Ciprofloxacin	192	8 (6.8/10.4)	218	8 (6.4/11.9)
Colistin[e,f]	201	4 (NA/88.6)	266	8 (NA/88.4)
Gentamicin	192	32 (28.7/25.5)	218	32 (34.9/32.6)
Imipenem[g]	235	16 (1.7/11.9)	310	16 (1.3/NA)
Levofloxacin	235	16 (11.9/13.6)	311	16 (10.3/17.0)
Meropenem	235	32 (4.7/26.4)	311	32 (8.7/37.3)
Piperacillin/tazobactam	235	256 (0.4/0.4)	311	256 (0.3/0.3)
Tigecycline[h,i,j]	210	2 (94.8/83.3)	271	2 (91.1/94.4)
**Latin America (N= 326)**				
Aztreonam-avibactam[c]	110	0.25 (100)	148	0.5 (100)
Aztreonam	110	256 (23.6/23.6)	148	128 (27.0/27.0)
Amikacin	113	128 (40.7/33.6)	155	128 (61.9/51.0)
Cefepime	113	64 (0/0)	155	64 (0/3.9)
Ceftazidime	113	256 (0/0)	155	256 (0/0)
Ceftazidime-avibactam	110	256 (0.9/0.9)	148	256 (0.7/0.7)
Ceftriaxone	19	64 (0/0)	24	64 (0/0)
Ciprofloxacin	94	8 (14.9/16.0)	131	8 (10.7/13.0)

Colistin[e,f]	94	2 (NA/90.4)	130	1 (NA/91.5)
Gentamicin	94	32 (17.0/14.9)	131	32 (31.3/30.5)
Imipenem[g]	110	16 (0/NA)	148	16 (0.7/NA)
Levofloxacin	113	16 (26.6/42.5)	155	16 (20.0/35.5)
Meropenem	113	32 (3.5/31.0)	155	32 (3.2/26.5)
Piperacillin/tazobactam	113	256 (0.9/0.9)	155	256 (0.7/0.7)
Tigecycline[h,i,j]	98	2 (94.9/90.9)	139	2 (94.2/93.3)
**North America (N=22)**				
Aztreonam-avibactam[c]	5	NA[d] (100)	14	0.5 (100)
Aztreonam	5	NA[d] (40.0/40.0)	14	128 (14.3/14.3)
Amikacin	5	NA[d] (80.0/60.0)	14	128 (50.0/50.0)
Cefepime	5	NA[d] (0/0)	14	64 (0/0)
Ceftazidime	5	NA[d] (0/0)	14	256 (0/0)
Ceftazidime-avibactam	5	NA[d] (0/0)	14	256 (0/0)
Ceftriaxone	NA	NA	1	NA[d] (0/0)
Ciprofloxacin	5	NA[d] (40.0/60.0)	13	8 (7.7/15.4)
Colistin[e,f]	5	NA[d] (NA/80.0)	13	0.5 (NA/92.3)
Gentamicin	5	NA[d] (60.0/60.0)	13	32 (0/0)
Imipenem[g]	5	NA[d] (0/20.0)	14	16 (0/7.1)
Levofloxacin	5	NA[d] (60.0/80.0)	14	16 (14.3/14.3)
Meropenem	5	NA[d] (0/60.0)	14	32 (0/35.7)
Piperacillin/tazobactam	5	NA[d] (0/0)	14	128 (0/0)
Tigecycline[h,i,j]	5	NA[d] (100/NA)	14	1 (92.9/0)

[a] Data includes percentage isolates susceptible at increased exposure. [b] Not all drugs in the panel were tested every year. [c] No breakpoints available from CLSI and EUCAST. Values expressed are indicative of the cumulative percentage of isolates inhibited at ≤8 mg/L for comparison purposes. [d] The MIC_{90} has been presented as NA considering the n<10. [e] Susceptible category for colistin not available for CLSI breakpoints (only intermediate and resistant isolates are available). [f] Data for colistin do not include isolates of *Morganella morganii, Proteus hauseri, Proteus mirabilis, Proteus vulgaris, Providencia alcalifaciens, Providencia rettgeri, Providencia* spp, *Providencia stuartii*, and *Serratia marcescens*. [g] Data for imipenem not available per EUCAST. [h] Data for tigecycline do not include isolates of *Morganella morganii, Proteus hauseri, Proteus mirabilis, Proteus vulgaris, Providencia alcalifaciens, Providencia rettgeri, Providencia* spp, and *Providencia stuartii*. [i] Data for tigecycline was calculated based on FDA approved breakpoints for CLSI. [j] EUCAST data for susceptibility to tigecycline are limited to *E. coli* and *C. koseri;* denominator (n): AfME: ICU=3, non-ICU=9; APAC: ICU=67, non-ICU=75; Europe: ICU=6, non-ICU=18; LATAM: ICU=11, non-ICU=15; North America: ICU=NA, non-ICU=1. ICU, intensive care unit; MIC, minimum inhibitory concentration; N, total number of isolates; n, number of isolates from wards; NA, not available.

Table S10. *In vitro* activity of ATM-AVI and comparator agents tested against MBL-positive Enterobacterales isolates across regions stratified by infection sources from 2016-2020.

	MIC_{90} (µg/mL) (% S, CLSI/%S, EUCAST[a])									
	RTI		UTI		SSTI		BSI		IAI	
**Africa-Middle East (N= 190)**	**n[b]**		**n[b]**		**n[b]**		**n[b]**		**n[b]**	
Aztreonam-avibactam[c]	24	0.25 (100)	59	1 (100)	38	0.5 (100)	54	0.25 (100)	15	0.25 (100)
Aztreonam	24	256 (20.8/20.8)	59	256 (28.8/28.8)	38	256 (13.2/13.2)	54	128 (11.1/11.1)	15	128 (40.0/40.0)
Amikacin	24	128 (54.2/33.3)	59	128 (71.2/55.9)	38	128 (57.9/42.1)	54	128 (50.0/37.0)	15	128 (66.7/53.3)

Cefepime	24	64 (0/0)	59	64 (3.4/8.5)	38	64 (0/0)	54	64 (0/1.9)	15	64 (20.0/20.0)
Ceftazidime	24	256 (0/0)	59	256 (0/0)	38	256 (0/0)	54	256 (0/0)	15	256 (6.7/6.7)
Ceftazidime-avibactam	24	256 (8.3/8.3)	59	256 (0/0)	38	256 (2.6/2.6)	54	256 (0/0)	15	256 (6.7/6.7)
Ceftriaxone	3	NA[d] (0/0)	4	NA[d] (0/0)	6	NA[d] (0/0)	6	NA[d] (0/0)	4	NA[d] (0/0)
Ciprofloxacin	21	8 (14.3/14.3)	55	8 (5.5/12.7)	32	8 (3.1/9.4)	48	8 (12.5/16.7)	11	8 (18.2/27.3)
Colistin[e,f]	22	1 (NA/90.9)	44	4 (NA/88.6)	34	1 (NA/100)	49	1 (NA/98.0)	12	1 (NA/100)
Gentamicin	21	32 (47.6/47.6)	55	32 (27.3/27.3)	32	32 (34.4/34.4)	48	32 (27.1/22.9)	11	32 (45.5/45.5)
Imipenem[g]	24	16 (4.2/8.3)	59	16 (1.7/6.8)	38	16 (0/NA)	54	16 (0/7.4)	15	16 (0/26.7)
Levofloxacin	24	16 (16.7/20.8)	59	16 (18.6/27.1)	38	16 (10.5/18.4)	54	16 (25.9/38.9)	15	16 (26.7/26.7)
Meropenem	24	32 (4.2/16.7)	59	32 (3.4/32.2)	38	32 (2.6/39.5)	54	32 (3.7/20.4)	15	32 (6.7/46.7)
Piperacillin/tazobactam	24	256 (4.2/4.2)	59	128 (1.7/1.7)	38	256 (0/0)	54	128 (1.9/1.9)	15	256 (0/0)
Tigecycline[h,i,j]	23	2 (95.7/NA)	48	2 (97.9/100)	34	2 (94.1/100)	52	1 (98.1/100)	12	4 (83.3/100)
**Asia-Pacific (N= 735)**										
Aztreonam-avibactam[c]	172	2 (98.8)	190	4 (97.4)	100	2 (99)	148	4 (98.7)	54	2 (98.2)
Aztreonam	185	256 (12.4/12.4)	205	256 (14.2/14.2)	109	256 (11.0/11.0)	176	256 (10.8/10.8)	56	256 (10.7/10.7)
Amikacin	185	128 (42.2/34.6)	207	128 (32.9/27.1)	109	128 (50.5/41.3)	176	128 (32.4/24.4)	56	128 (55.4/48.2)
Cefepime	185	64 (0/0.5)	207	64 (0.5/1.9)	109	64 (0/0.9)	176	64 (0/0.6)	56	64 (0/0)
Ceftazidime	185	256 (0/0)	207	256 (0/0)	109	256 (0/0)	176	256 (0/0)	56	256 (0/0)
Ceftazidime-avibactam	185	256 (0/0)	205	256 (1.0/1.0)	109	256 (0/0)	176	256 (0.6/0.6)	56	256 (0/0)
Ceftriaxone	39	32 (0/0)	28	32 (0/0)	23	32 (0/0)	5	32 (0/0)	7	32 (0/0)
Ciprofloxacin	146	8 (2.1/4.8)	179	8 (1.7/2.8)	86	8 (4.7/5.8)	171	8 (5.9/8.8)	49	8 (2.0/6.1)
Colistin[e,f]	174	4 (NA/89.7)	182	1 (NA/94.5)	99	1 (NA/93.9)	159	1 (NA/93.7)	55	1 (NA/90.9)
Gentamicin	146	32 (26.0/26.0)	179	32 (16.8/16.2)	86	32 (29.1/29.1)	171	32 (26.3/24.0)	49	32 (34.7/32.7)
Imipenem[g]	185	16 (1.6/8.1)	205	16 (1.0/6.3)	109	16 (1.8/NA)	176	16 (0.6/NA)	56	16 (5.4/8.9)
Levofloxacin	185	16 (5.4/11.4)	207	16 (3.4/6.3)	109	16 (8.3/12.8)	176	16 (10.2/15.3)	56	16 (3.6/8.9)
Meropenem	185	32 (2.7/15.7)	207	32 (1.9/10.6)	109	32 (1.8/14.7)	176	32 (0.6/5.7)	56	32 (1.8/12.5)
Piperacillin/tazobactam	185	256 (3.2/3.2)	207	256 (1.5/1.5)	109	256 (2.8/2.8)	176	128 (0.6/0.6)	56	128 (1.8/1.8)
Tigecycline[h,i,j]	178	2 (92.7/93.1)	186	2 (94.6/90.9)	100	4 (90.0/100)	164	2 (97.6/94.3)	56	2 (92.9/81.8)
**Europe (N= 604)**										
Aztreonam-avibactam[c]	179	0.5 (100)	138	0.5 (99.3)	96	0.5 (100)	111	0.5 (100)	78	1 (100)
Aztreonam	179	256 (15.6/15.6)	138	256 (27.5/27.5)	96	256 (27.1/27.1)	111	256 (20.7/20.7)	78	256 (12.8/12.8)
Amikacin	179	128 (46.4/35.2)	139	128 (47.5/39.6)	97	128 (54.6/35.1)	111	128 (55.9/41.4)	78	128 (61.5/48.7)
Cefepime	179	64 (0/0.6)	139	64 (2.2/3.6)	97	64 (3.1/5.2)	111	64 (0/0)	78	64 (5.1/6.4)
Ceftazidime	179	256 (0/0)	139	256 (0/0)	97	256 (0/0)	111	256 (0/0)	78	256 (0/0)
Ceftazidime-avibactam	179	256 (3.9/3.9)	138	256 (2.9/2.9)	96	256 (14.6/14.6)	111	256 (3.6/3.6)	78	256 (3.9/3.9)
Ceftriaxone	34	32 (0/0)	33	32 (0/0)	37	32 (0/0)	17	32 (0/0)	33	32 (0/0)
Ciprofloxacin	145	8 (6.2/9.0)	106	8 (8.5/13.2)	60	8 (3.3/6.7)	94	8 (6.4/13.8)	45	8 (4.4/6.7)
Colistin[e,f]	160	4 (NA/89.4)	115	16 (NA/82.6)	79	1 (NA/93.7)	88	4 (NA/87.5)	75	16 (NA/86.7)

Gentamicin	145	32 (29.7/27.6)	106	32 (34.0/30.2)	60	32 (38.3/35.0)	94	32 (31.9/27.7)	45	32 (31.1/26.7)
Imipenem[g]	179	16 (1.7/13.4)	138	16 (0/NA)	96	16 (3.1/15.6)	111	16 (0.9/9.0)	78	16 (1.3/NA)
Levofloxacin	179	16 (12.3/14.5)	139	16 (12.2/21.6)	97	16 (8.3/11.3)	111	16 (9.9/18.0)	78	16 (6.4/10.3)
Meropenem	179	32 (6.2/28.5)	139	32 (7.2/28.8)	97	32 (8.3/42.3)	111	32 (5.4/33.3)	78	32 (10.3/39.7)
Piperacillin/tazobactam	179	256 (0/0)	139	256 (1.4/1.4)	97	256 (0/0)	111	256 (0/0)	78	256 (0/0)
Tigecycline[h,i,j]	166	2 (95.8/75.0)	118	2 (93.2/100)	81	2 (92.6/85.7)	92	2 (92.4/100)	75	4 (88.0/100)
**Latin America (N= 326)**										
Aztreonam-avibactam[c]	41	0.25 (100)	75	0.25 (100)	77	0.5 (100)	85	0.25 (100)	38	0.25 (100)
Aztreonam	41	128 (22.0/22.0)	75	128 (41.3/41.3)	77	256 (23.4/23.4)	85	128 (22.4/22.4)	38	256 (21.1/21.1)
Amikacin	41	128 (53.7/36.6)	76	128 (52.6/42.1)	78	128 (52.6/38.5)	89	128 (53.9/48.3)	41	128 (61.0/58.5)
Cefepime	41	64 (0/2.4)	76	64 (0/4.0)	78	64 (0/1.3)	89	64 (0/0)	41	64 (0/4.9)
Ceftazidime	41	256 (0/0)	76	256 (0/0)	78	256 (0/0)	89	256 (0/0)	41	256 (0/0)
Ceftazidime-avibactam	41	256 (0/0)	75	256 (1.3/1.3)	77	256 (1.3/1.3)	85	256 (0/0)	38	256 (0/0)
Ceftriaxone	2	NA[d] (0/0)	9	NA[d] (0/0)	11	32 (0/0)	16	64 (0/0)	8	NA[d] (0/0)
Ciprofloxacin	39	8 (10.3/15.4)	67	8 (9.0/10.5)	67	8 (10.5/13.4)	73	8 (19.2/20.6)	33	8 (18.2/18.2)
Colistin[e,f]	35	1 (NA/94.3)	57	4 (NA/89.5)	67	1 (NA/94.0)	76	2 (NA/90.8)	34	4 (NA/88.2)
Gentamicin	39	32 (20.5/18.0)	67	32 (17.9/16.4)	67	32 (26.9/25.4)	73	32 (30.1/27.4)	33	32 (45.5/42.4)
Imipenem[g]	41	16 (0/7.3)	75	16 (1.3/NA)	77	16 (0/9.1)	85	16 (0/5.9)	38	16 (0/NA)
Levofloxacin	41	16 (24.4/43.9)	76	16 (14.5/30.3)	78	16 (21.8/43.6)	89	16 (24.7/40.5)	41	16 (26.8/43.9)
Meropenem	41	32 (7.3/26.8)	76	32 (4.0/31.6)	78	32 (1.3/20.5)	89	32 (1.1/28.1)	41	32 (2.4/29.3)
Piperacillin/tazobactam	41	128 (2.4/2.4)	76	128 (1.3/1.3)	78	256 (0/0)	89	256 (1.1/1.1)	41	256 (0/0)
Tigecycline[h,i,j]	36	2 (91.7/100)	58	2 (96.6/80.0)	69	2 (97.1/90.9)	81	2 (91.4/100)	37	2 (100/100)
**North America (N=22)**										
Aztreonam-avibactam[c]	7	NA[d] (100)	6	NA[d] (100)	5	NA[d] (100)	NA	NA	3	NA[d] (100)
Aztreonam	7	NA[d] (14.3/14.3)	6	NA[d] (50.0/50.0)	5	NA[d] (60.0/60.0)	NA	NA	3	NA[d] (0/0)
Amikacin	7	NA[d] (57.1/57.1)	6	NA[d] (66.7/66.7)	5	NA[d] (100/80.0)	NA	NA	3	NA[d] (0/0)
Cefepime	7	NA[d] (0/0)	6	NA[d] (0/0)	5	NA[d] (0/0)	NA	NA	3	NA[d] (0/0)
Ceftazidime	7	NA[d] (0/0)	6	NA[d] (0/0)	5	NA[d] (0/0)	NA	NA	3	NA[d] (0/0)
Ceftazidime-avibactam	7	NA[d] (0/0)	6	NA[d] (0/0)	5	NA[d] (0/0)	NA	NA	3	NA[d] (0/0)
Ceftriaxone	NA	NA	NA	NA	1	NA[d] (0/0)	NA	NA	NA	NA
Ciprofloxacin	7	NA[d] (14.3/28.6)	6	NA[d] (0/0)	4	NA[d] (50.0/100)	NA	NA	3	NA[d] (0/0)
Colistin[e,f]	7	NA[d] (NA/71.4)	5	NA[d] (NA/100)	5	NA[d] (NA/100)	NA	NA	3	NA[d] (NA/100)
Gentamicin	7	NA[d] (0/0)	6	NA[d] (0/0)	4	NA[d] (100/100)	NA	NA	3	NA[d] (0/0)
Imipenem[g]	7	NA[d] (0/0)	6	NA[d] (0/16.7)	5	NA[d] (0/40.0)	NA	NA	3	NA[d] (0/0)
Levofloxacin	7	NA[d] (28.6/42.9)	6	NA[d] (0/16.7)	5	NA[d] (60.0/80.0)	NA	NA	3	NA[d] (0/0)
Meropenem	7	NA[d] (0/28.6)	6	NA[d] (0/33.3)	5	NA[d] (0/100)	NA	NA	3	NA[d] (0/33.3)

Piperacillin/tazobactam	7	NA[d] (0/0)	6	NA[d] (0/0)	5	NA[d] (0/0)	NA	NA	3	NA[d] (0/0)
Tigecycline[h,i,j]	7	NA[d] (100/NA)	6	NA[d] (83.3/NA)	5	NA[d] (100/NA)	NA	NA	3	NA[d] (100/0)

[a] Not all drugs in the panel were tested every year. [b] Data includes percentage isolates susceptible at increased exposure. [c] No breakpoints available from CLSI and EUCAST. Values expressed are indicative of the cumulative percentage of isolates inhibited at ≤8 mg/L for comparison purposes. [d] The MIC_{90} has been presented as NA considering the n<10. [e] Susceptible category for colistin not available for CLSI breakpoints (only intermediate and resistant isolates are available). [f] Data for colistin do not include isolates of *Morganella morganii*, *Proteus hauseri*, *Proteus mirabilis*, *Proteus vulgaris*, *Providencia alcalifaciens*, *Providencia rettgeri*, *Providencia* spp, *Providencia stuartii*, and *Serratia marcescens*. [g] Data for imipenem not available per EUCAST. [h] Data for tigecycline do not include isolates of *Morganella morganii*, *Proteus hauseri*, *Proteus mirabilis*, *Proteus vulgaris*, *Providencia alcalifaciens*, *Providencia rettgeri*, *Providencia* spp, and *Providencia stuartii*. [i] Data for tigecycline was calculated based on FDA approved breakpoints for CLSI. [j] EUCAST data for susceptibility to tigecycline are limited to *E. coli* and *C. koseri;* denominator (n): AfME: RTI=NA, UTI=7, SSTI=8, BSI=2, IAI=2; APAC: RTI=29, UTI=66, SSTI=18, BSI=35, IAI=11; Europe: RTI=4, UTI=7, SSTI=7, BSI=2, IAI=5; LATAM: RTI=3, UTI=5, SSTI=11, BSI=8, IAI=4; North America: RTI=NA, UTI=NA, SSTI=NA, BSI=NA, IAI=1. BSI, bloodstream infections; IAI, intra-abdominal infection; MIC, minimum inhibitory concentration; N, total number of isolates; n, number of isolates from infection sources; NA, not available; RTI, respiratory tract infection; SSTI, skin and soft tissue infection; UTI, urinary tract infection.
